# Online Medical Control for EMS: A Lecture and Case-Based Teaching Module

**DOI:** 10.15766/mep_2374-8265.10902

**Published:** 2020-05-15

**Authors:** Frank W. Tift, Jose V. Nable

**Affiliations:** 1 Clinical Assistant Professor, Department of Emergency Medicine, University of Tennessee College of Medicine—Chattanooga; Associate Program Director—EMS Fellowship, Department of Emergency Medicine, University of Tennessee College of Medicine—Chattanooga; 2 Assistant Professor, Department of Emergency Medicine, MedStar Georgetown University Hospital

**Keywords:** EMS, Prehospital, Online Medical Control, OLMC, Direct Medical Oversight, Paramedic, EMT, Emergency Medicine, Case-Based Learning, Clinical Skills Assessment/OSCEs, Clinical/Procedural Skills Training

## Abstract

**Introduction:**

The provision of real-time medical direction to emergency medical services (EMS) providers is a core skill for the emergency physician, yet it is one with a wide variability of training received within residency.

**Methods:**

We developed a complete training module for providing online medical control to EMS providers, including two lectures, multiple case-based scenarios for practice via two-way radio, a survey of participants’ self-perceived knowledge and comfort in this area, and a postmodule knowledge test. Participants completed the survey both before and after the module. The module was given during the regularly scheduled didactic conference series. There were 22 participants, some of whom were attendings and medical students.

**Results:**

The survey responses showed a statistically significant improvement after completion of the module for all questions, including improved self-perceived comfort with providing online medical control. Additionally, all participants passed the postmodule knowledge test with a mean score of 95%.

**Discussion:**

This module was well received and showed significant results in improving the participants’ self-perceived and tested knowledge of EMS as well as their comfort with providing online medical control. The module offers an excellent baseline training experience for use by other residencies or agency medical directors.

## Educational Objectives

By the end of this module, learners will be able to:

1. List the differences between the four training levels of prehospital providers.
2. List the differences between the most common types of emergency medical services (EMS) agencies.
3. Discuss the role of the physician medical director within the EMS system.
4. Demonstrate proper application and understanding of the online medical control process, including the use of a handheld radio with proper technique and etiquette.
5. Provide orders to simulated EMS units safely and appropriately.

## Introduction

The provision of real-time medical direction to emergency medical services (EMS) providers, either over radio or by phone, is a core skill of the emergency medicine physician.^1^ However, there is a wide variety of systems for providing online medical control (OLMC) such that some physicians do so regularly and others not at all. This variety of OLMC exposure could be partially due to an overall decrease in the use of OLMC by EMS services in favor of standing order protocols in the wake of literature showing faster scene times and decreased errors when using protocols.^2^ Despite this trend, systems still recognize the need for OLMC in certain situations,^3^ and there are even calls from EMS providers for a better and more collaborative relationship between EMS providers and physicians with specific EMS training.^4^

Published literature on the teaching of EMS topics to resident physicians is limited. Examples of proposed objectives for an EMS rotation as well as model curricula for an EMS rotation can be found.^5,6^ These examples do include the topic of OLMC, but the degree to which it is addressed can be variable. This skill in particular has the potential to be a daily part of an emergency physician's practice, and it is one that can have potential legal repercussions if done poorly.^7^ Legal requirements for who may give OLMC vary greatly from state to state. Some states make no specific mention of OLMC requirements, while others may require short courses, paperwork, and licensure verification. For example, Pennsylvania includes requirements for a medical command physician within its state code.^8^ Ultimately, it is the responsibility of an EMS agency's medical director to ensure the proper training and quality of physicians giving OLMC to the prehospital providers under his or her supervision.^9^ Therefore, the development of training modules specific to the provision of OLMC for use by residency programs and agency medical directors would be prudent.

Other courses do exist, particularly from states like Pennsylvania where more stringent requirements are in place, and some include case-based discussion or examples. However, we were unable to find any courses that included scenarios meant to be given in a live simulation environment. A search of *MedEdPORTAL* did not reveal any such publications. The following search terms were used: *EMS, prehospital, prehospital medicine, OLMC, online medical control, online medical command, medical command, medical control, direct oversight,* and *paramedic.* To address this need, we developed a training module for the provision of OLMC. Given that students react well to case-based learning and feel that it enhances their educational experience,^10^ this module includes not only lecture-based information but also case-based scenarios utilizing a two-way radio to allow more realistic practice of OLMC skills. The goal was to enhance the learners’ knowledge of EMS systems as well as of the different provider levels and what each can do, which, along with simulated practice giving online orders, would improve learners’ ability to provide orders to prehospital providers over the radio.

## Methods

### Development

We developed a module to be given as a portion of our standard weekly didactic sessions, and the study data were collected during the first use of this module. The institution was an academic, level 1 trauma center in an urban/suburban area with a 3-year emergency medicine residency and an approximate annual emergency department volume of 55,000 visits per year. It received 334 radio requests for physician orders in 2017. These requests were expected to be answered by a senior resident, and thus, our primary target audience was PGY 2 emergency medicine residents who were being credentialed to answer the radio. However, all residents, attendings, and medical students were invited to attend either for early exposure or for a review of the topics. At this point in the development of this process, participation in the module and successful completion of the posttest were the requirements for credentialing. We developed eight scenarios (Appendix A) based on our personal experience, common order requests, and related topics of importance (patient refusals, trauma triage criteria, etc.). We chose to write eight scenarios due to the number of rising residents as well as the time allotted. The lectures (Appendices B and C) were written by the primary author. Topics included the different levels of EMS providers and their capabilities, different types of EMS agencies, structure of the 911 system, role of the EMS medical director and how he or she supervises the system, how OLMC fits into that supervision, how to provide OLMC, and legal issues surrounding OLMC. Both the lectures and the scenarios were presented in a 2.5-hour block of the standard weekly resident didactics. No prior knowledge was required for any learners, though some prior reading was recommended within the scenarios themselves (Appendix A). The PGY 2 residents also had some prior exposure to OLMC during their intern year in that they were often present when the radio was answered by a senior resident or attending. The survey (Appendix D) and knowledge test (Appendix E) were written by the primary author and reviewed by the second author for appropriateness.

### Equipment/Environment

The module consisted of two lectures (Appendices B and C), a set of eight scenarios (Appendix A), a self-assessment survey covering EMS knowledge and comfort providing OLMC (Appendix D), a multiple-choice knowledge test (Appendix E), and lecture outlines to be used as optional handouts (Appendix F). Lecture presentations could be presented in any lecture hall/room that had appropriate audiovisual equipment for learners to view a computer-based lecture and that could comfortably seat all present. No specific prereading was required, though some prior reading was recommended within the scenarios themselves (Appendix A), which could be provided beforehand. Participants were also ideally familiar with local EMS protocol prior to the session. While the scenarios were designed to be presented immediately following the lectures, they could be offered at a different time or location, and they did not require any particular facilities save a space suitable for group discussion and arranged so that the presenter could step out of the room in order to be physically separated from the learners (i.e., unable to see or hear them). We presented the scenarios immediately following the lectures as planned and using the same lecture hall in which the lectures were given. This lecture hall was a standard one with rows of seating facing a large TV, which projected the lecture. We designed the scenarios to utilize a two-way radio of any type so that the presenter could step out of the room, assume the role of a prehospital provider requesting orders, and communicate with the learners only by radio during the scenario as a means of simulating a true request for orders. Similar radios were necessary to run the scenarios, but they could be of any brand or model such that two people could communicate from separate rooms over a single channel. We also recommend using equipment to record the conversation so that it can be reviewed during the debrief.

### Personnel

We targeted the content to emergency medicine residents; however, attendings and medical students were also welcome to participate. This module could also be used by a community EMS medical director to credential his or her colleagues to provide OLMC for his or her EMS agency. Because we prepared eight scenarios, we designed the module to have no more than eight learners who required credentialing in order to allow each to directly participate (i.e., use the radio) in one scenario, though more could be present who did not require specific credentialing. However, we would limit the total number to a group of 20-25 to facilitate discussion of each scenario. Should more than eight participants require credentialing, another facilitator could be used in order to split the group. Ideally, small groups of four people would be optimal and allow learners to directly participate (i.e., use the radio) in two scenarios. No more than eight participants requiring credentialing should be allowed per facilitator. Experienced senior residents or EMS fellows could be used as facilitators to help with personnel needs and to provide EMS teaching experience to those with particular interest in EMS. In our session, the primary author was the presenter. He was board certified in EMS. We recommend that future presenters and any facilitators already be familiar with EMS topics and have experience with providing OLMC, but this recommendation is not a requirement. Preparation via review of the material should be sufficient for any emergency physician to act as presenter or facilitator.

### Implementation

The lectures and scenarios were presented in a 2.5-hour block during the standard weekly resident didactic session. Immediately at the beginning of the module, all in attendance were asked to complete a short survey in which learners offered a self-assessment of basic EMS knowledge and comfort with providing OLMC (Appendix D). Once all learners had completed the survey, the lectures were presented beginning with the Intro to EMS (Appendix B) and followed by Medical Oversight of EMS (Appendix C). After the lectures, the scenarios (Appendix A) were used as a low-fidelity simulation of requests for orders from a prehospital provider. One radio was given to a single resident who would run the scenario. The facilitator then exited the room and assumed the role of the prehospital provider requesting orders based on the scenario notes provided and utilizing the other radio to communicate with the resident in the room. At the end of each scenario, the facilitator returned to the room to debrief the scenario and discuss any important topics that the scenario addressed. A different resident was selected to hold the radio and participate in the next scenario, and the process continued until all scenarios had been presented. At the end of the module, all in attendance were asked to complete the same survey again (Appendix D) as well as a 20-question test covering basic EMS knowledge (Appendix E).

### Assessment

All residents, students, and attendings present completed the same survey (Appendix D) at both the beginning and the end of the module. All learners also completed a short test covering basic EMS knowledge (Appendix E) at the end of the module. All knowledge necessary to answer the test questions was covered in the two lectures. The surveys and tests were collected at the end of the module, and these data were compiled into a spreadsheet to compare pre- and postmodule survey results as well as to report overall scores on the posttest. Additionally, each of the scenarios included basic evaluation objectives to discuss with residents during the debrief for that particular scenario. The statistical analysis of the data for this study used a paired *t* test to assess for significance in the change between pre- and postmodule survey data. The statistical software utilized was SPSS Version 24 (IBM, Somers, New York). The Institutional Review Board at the University of Tennessee College of Medicine—Chattanooga reviewed this study and deemed it to qualify for exemption.

## Results

There were 22 participants in the module, including some from every training level (Table 1). All participants completed the survey, in which questions were answered on a 5-point Likert scale (1 = *strongly disagree,* 5 = *strongly agree*), both before and after the module. All participants also completed the posttest. There was a significant improvement in the mean survey response for all questions after completion of the module (Table 2). There was a corresponding increase in the median value for each question as well. Of particular note, the participants’ self-perceived comfort with providing OLMC increased from a mean rating of 2.4 to 3.9 (difference: 1.5; 95% confidence interval, 0.8-2.0; *p* < .01), with a corresponding increase in the median response value from 2 to 4. All participants also passed the posttest with high scores, resulting in a mean test score of 95%.

**Table 1.**
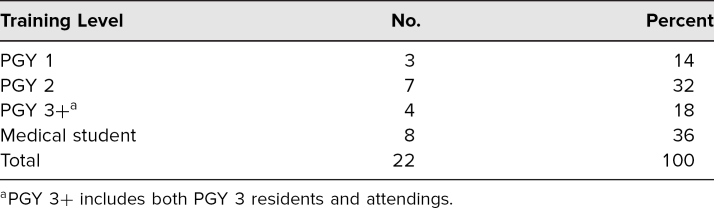
Participant Types

**Table 2.**
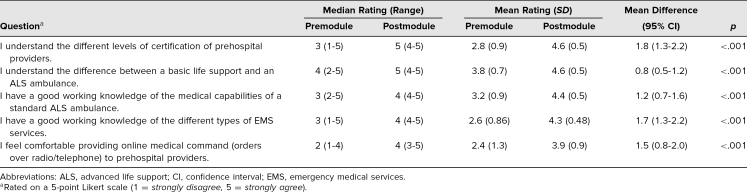
Overall Comparison of Median and Mean Values (*N* = 22)

We also completed subgroup analyses comparing the change in survey responses within each of the training levels present. We present the mean and median data for the PGY 2 residents (Table 3) as they were our primary target population and one of two subgroups with a larger number present. Note that the data from the PGY 2 residents are very similar to those of the overall cohort. Additionally, the range of scores on the posttest for the PGY 2 residents was 95%-100%.

**Table 3.**
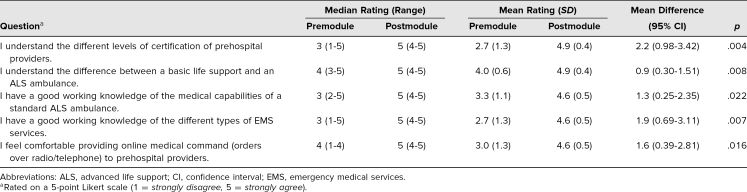
Comparison of Median and Mean Values for PGY 2 Residents (*N* = 7)

## Discussion

As a niche topic within emergency medicine, EMS can be easily overlooked in the competition for didactic time with the breadth of the primary specialty. However, this one topic is a daily part of practice for most emergency physicians if only in the interaction with prehospital providers who transport patients to the emergency department. For many others, the provision of OLMC is a common event, and thus, training in this skill is essential. At our institution, this module was well received by residents, many of whom recognized the deficiency of their knowledge and skills in this area as potentially embarrassing when interacting with prehospital providers upon arrival at the hospital.

The results showed a significant increase in participants’ self-assessment of EMS knowledge and comfort with providing OLMC. Additionally, all participants passed the posttest with very high scores, an outcome we expect would have been quite different if participants had taken the test prior to attending the module. These results are encouraging and suggest that the module had a significant positive impact on the participants’ knowledge and training. Of note, the subgroup analysis of PGY 2 residents was very similar to the overall data. However, the subgroup analysis of medical students showed lower premodule ratings in several categories that improved to similar postmodule ratings, except for perceived comfort with providing OLMC. This finding suggests that the module provides excellent teaching and knowledge improvement even for those with minimal background experience, whereas perceived comfort with providing OLMC could be more dependent on actively participating in the scenarios, experience in emergency medicine, or both. One challenge to presenting the scenarios that we noted is to ensure that the resident is given the appropriate preparatory information for the scenario, most importantly in which level of ED he or she is meant to be working. If this information is forgotten or neglected, the resident will inevitably assume the role of a provider at his or her primary institution, which may greatly affect the outcome of the scenario. We encourage others using our scenarios to modify them to match appropriate facilities in their area that meet the needs of the scenario.

There are two primary limitations to this study. First, the number of participants was relatively small, which was due to studying the module at a single institution. Additionally, while the residents were expected to maintain a certain attendance percentage, they could not be required to attend every session, so we were also subject to some degree of chance in how many participants were present. Despite this limitation, we feel that the significance of change in survey responses was enough to publish given the limitations of survey data and that waiting until the module could be presented at another institution was unlikely to significantly change the results. Second, our method of before-and-after comparison has its own limitations. The use of a survey as the primary mode of before-and-after comparison comes with the inherent limitations of survey data, which may not accurately represent how the participants would perform in a true situation. We had intended the participants to complete the knowledge test prior to the module as well to compare test scores, but only two participants had done so upon arrival, and we did not have enough time set aside within the didactic schedule to allow test completion before the module on the day of presentation. Even more ideal would be a means of comparing radio responses to requests for orders before and after the module was completed. However, there is no standardized rubric for assessing the quality of such communications, and so, we were unable to complete such a comparison. This limitation does provide an opportunity for future study. Such a rubric for quality of OLMC interactions could be developed and this study repeated with future residents to more accurately assess the educational quality of the module.

This module was very well received by our residents, and we intend to continue to use it for basic EMS and OLMC education at least annually. We will continue to refine the educational content as well as the means of measuring effectiveness. Possible future changes include ensuring that the residents complete the test before the module in addition to after; repeating the survey, the scenarios, or both halfway through the year to ensure retention of the information and skills; and creating a standardized means of measuring improvement of actual EMS radio interactions.

## Appendices

OLMC Scenarios.docxIntro to EMS.pptxMedical Oversight of EMS.pptxSurvey.docxTest and Key.docxLecture Outlines.docx
*All appendices are peer reviewed as integral parts of the Original Publication.*
