# Supplementary material for: Online Medical Control for EMS: A Lecture and Case-Based Teaching Module
Source: MedEdPORTAL. 2020 May 15;16:10902. doi: 10.15766/mep_2374-8265.10902 (PMC7331954; doi:10.15766/mep_2374-8265.10902)
Supplement: Supplementary file 1 — OLMC Scenarios.docxIntro to EMS.pptxMedical Oversight of EMS.pptxSurvey.docxTest and Key.docxLecture Outlines.docx [file mep_2374-8265.10902-s001.zip › A. OLMC Scenarios.docx]

| **Appendix A: OLMC Scenarios – Case 1**  **SIMULATION CASE TITLE: EMS OLMC – Altered Mental Status**  **AUTHORS: Jose V. Nable, MD and Frank Tift, MD**  **LEARNER AUDIENCE: Emergency Medicine residents and medical students** | |
| --- | --- |
| **PATIENT NAME: N/A (patient names are not given over the radio)**  **PATIENT AGE: 62**  **CHIEF COMPLAINT: altered mental status, possible stroke**  **PHYSICAL SETTING: Learner is working in an Emergency Department that provides online medical control to EMS units when he/she is contacted (by radio) by an EMS unit requesting orders.** | |
|  | |
| **Brief narrative description of case** | In this scenario, a prehospital provider is requesting orders from the learner over a radio for a male patient who has altered mental status. The prehospital provider is concerned that the patient may be having a stroke, but after the learner asks appropriate questions, it is discovered that the prehospital provider neglected to check the patient’s blood sugar, which is low. Therapy for hypoglycemia should then be initiated. |
| **Primary Learning Objectives** | By the end of this scenario, learners will be able to:   - Demonstrate proper use of the radio - Recognize the importance of asking for additional information - Recall that hypoglycemia is a potential stroke mimic - Give appropriate orders for hypoglycemia treatment |
| **Critical Actions** | 1. Utilizes the radio appropriately 2. Probes the prehospital provider for additional information about potential stroke symptoms and potential mimics 3. Discovers that the patient is hypoglycemic 4. Orders IV dextrose and NOT oral glucose |
| **Learner Preparation or Prework** | The learner is an attending physician on duty in the emergency department at a regional stroke center.  No specific additional reading or prep work should be required for this scenario |

| Radio report | | | |
| --- | --- | --- | --- |
| **Overall Setting and Appearance** | No specific setting is required other than ensuring that the learner and facilitator are in separate rooms and able to communicate via a two-way radio | | |
| **Confederates (e.g., standardized participants) and their roles in the room at case start** | No additional personnel are required. | | |
| **Facilitator script for opening radio statement** | “Medic 1 to [OLMC]” *(feel free to insert an appropriate callsign for your local institution/area)*  After the learner acknowledges transmission:  “Medic 1 is en route to your facility with an 62-year-old male with a possible stroke. He has been mumbling his words for the last 2 hours. I can’t understand what he is saying, and he is drooling. I think you should activate the stroke team.”  All information below must be specifically requested by the learner | | |
| **Initial vital signs** | HR: 82, BP: 148/90, Resp: 14, Sats: 99% on RA | | |
| **Past Medical/Surgical History** | **Medications** | **Allergies** | **Family History** |
| Hypertension  Diabetes | Hydrochlorothiazide  Insulin | NKDA | Diabetes |
| **Physical Examination** | | | |
| **General** | Altered, slurred speech, somnolent, but eyes open to voice | | |
| **HEENT** | Normocephalic, atraumatic | | |
| **Neck** | Supple, no signs of trauma | | |
| **Lungs** | Non-labored breathing, clear to auscultation bilaterally | | |
| **Cardiovascular** | Normal S1/S2, regular rhythm, no murmurs | | |
| **Abdomen** | Soft, non-distended, non-tender | | |
| **Neurological** | Somnolent, inconsistently follows commands, no gross focal deficits, moving all extremities | | |
| **Skin** | Warm, clammy, no obvious rash | | |
| **GU** | *(Not examined)* | | |
| **Psychiatric** | Somnolent, unintelligible speech | | |

| Instructor Notes - Changes and CASE Branch Points | |
| --- | --- |
| **Intervention / Time point** | **Change in Case** |
| *Learner asks for a glucose level* | *“Oh, we forgot to check. Would you like us to?”……..”It’s 22! Should we give him some oral glucose?”* |
| *When asked about oral glucose* | Critical Action:   - Denies oral glucose request - Orders IV dextrose |

**Ideal Scenario Flow**

After receiving the initial report and request to activate the stroke team, the learner should recognize that the prehospital provider has not described any truly focal neurological deficit. While slurred speech can be a sign of stroke, it is rarely the only symptom. Additionally, global altered mental status it is very unusual as the only sign in patients with stroke. The learner should ask for more details about the physical exam in search of other signs of stroke. He/she should then also ask about blood glucose, as it is the most common stroke mimic. Upon learning of the patient’s hypoglycemia, the prehospital provider suggests giving oral glucose, which the learner should deny, as the patient may not tolerate oral medication in his current state. The learner should instruct the prehospital provider to administer IV dextrose and continue in to the hospital.

**Anticipated Management Mistakes**

1. Failure to ask for additional information: The facilitator may give a one-time hint that another etiology is the culprit. For example: “Okay, we wanted to tell you first since he doesn’t seem to be weak or numb anywhere.”
2. Failure to ask for a glucose level: Patient will arrive in the ED and be taken quickly to the CT scanner following the facility’s stroke protocol. The scenario will end, but at the beginning of the debrief, the facilitator should state that Neurologist cancelled the stroke alert shortly after the patient arrived at the scanner, as the stroke team checked his sugar and found him to be hypoglycemic. The patient returned to the ED alert and following all commands.
3. Failure to deny oral glucose: The patient arrives in the ED and must be taken into an ED room prior to going to the CT scanner for airway management. The prehospital providers believe that he aspirated on the oral glucose. The scenario will end, and further discussion may take place during the debrief.

Debrief materials

What are some common stroke mimics?

- Hypoglycemia, complex migraine, seizure with Todd’s paralysis, CNS tumor or abscess, head trauma, systemic infection, CNS infection, metabolic encephalopathy, etc.
- Hypoglycemia is the most common, and a blood sugar should be checked on every altered mental status patient prior to initiating treatment.

Why did the prehospital provider neglect to check the blood sugar?

- Simply forgot—all people can make mistakes

Why did the prehospital provider think this patient had a stroke?

- Altered mental status with slurred speech. The latter can be a sign of stroke, but is not typically an isolated sign, and this finding in isolation should prompt a search for other stroke symptoms or signs of stroke mimics. Altered mental status it is very unusual as the only sign in patients with stroke.

Why should you not give oral glucose to this patient?

- Slurred speech with drooling suggests difficulty with swallowing, and thus he is at risk for aspirating the oral glucose

Discussion

The emergency physician must be keenly aware that acute neurologic deficits and/or altered mental status are not always presentations for an ischemic stroke. Several other conditions can mimic, including hypoglycemia.^1^ Radio communications can make it challenging for the recipient of a report to understand what is happening on the scene. However, it is incumbent on the emergency physician to probe prehospital providers to provide more details as necessary in order to make appropriate medical command decisions.

In this patient with neurological deficits, a fingerstick was omitted by the prehospital provider. Soliciting this information resulted in discovering that the patient was actually hypoglycemic. While oral glucose is a mainstay of treatment for hypoglycemic patients, this scenario included a patient who was drooling and incoherent. Such a patient is a poor candidate for oral glucose due to the risks of aspiration.

References:

1. Magauran BG Jr, Nitka M. Stroke mimics. *Emerg Med Clin North Am* 2012;30:795-804.

| **Appendix A: OLMC Scenarios – Case 2**  **SIMULATION CASE TITLE: EMS OLMC - Head Trauma and Anticoagulant Use**  **AUTHORS: Jose V. Nable, MD and Frank Tift, MD**  **LEARNER AUDIENCE: Emergency Medicine residents and medical students** | |
| --- | --- |
| **PATIENT NAME: N/A (patient names are not given over the radio)**  **PATIENT AGE: 82**  **CHIEF COMPLAINT: fall with head injury and tachycardia**  **PHYSICAL SETTING: Learner is working in an Emergency Department that provides online medical control to EMS units when he/she is contacted (by radio) by an EMS unit requesting orders.** | |
|  | |
| **Brief narrative description of case** | In this scenario, a prehospital provider is requesting orders from the learner over a radio for a female patient after a fall with head injury when the patient is on anticoagulation. The patient is also in rapid atrial fibrillation, and the EMS crew is requesting orders to give an additional dose of diltiazem after having already given one dose. The learner should recognize that the situation meets criteria to take the patient to a trauma center, deny the request for diltiazem, and instruct the crew to transport to the closest trauma center. |
| **Primary Learning Objectives** | By the end of this scenario, learners will be able to:   - Demonstrate proper use of the radio - Recognize the importance of asking for additional information - Appropriately deny a potentially unsafe request for orders - Utilize the CDC Field Trauma Triage Guidelines appropriately |
| **Critical Actions** | 1. Utilizes the radio appropriately 2. Probes the prehospital provider for potential signs of trauma 3. Recognized that the patient meets criteria for evaluation at a trauma center 4. Denies the prehospital provider’s request for diltiazem |
| **Learner Preparation or Prework** | The learner is an attending physician on duty at Hospital A, which has a double coverage community ED that is a chest pain and cardiac center. There are two other hospitals in town, one of which is a trauma center. Hospital B is a level 2 trauma center with Neurosurgery coverage (as well as a STEMI center) and is about 15 minutes away. Hospital C has no specialty designations.  Learners should have reviewed the CDC Field Trauma Triage Criteria before the session  *(the specific hospital capabilities and distances can be withheld from the learner until he/she makes the determination that a trauma center is needed to prevent leading him/her towards the appropriate decision)* |

| Radio report | | | |
| --- | --- | --- | --- |
| **Overall Setting and Appearance** | No specific setting is required other than ensuring that the learner and facilitator are in separate rooms and able to communicate via a two-way radio | | |
| **Confederates (e.g., standardized participants) and their roles in the room at case start** | No additional personnel are required. | | |
| **Facilitator script for opening radio statement** | “Medic 2 to [OLMC]” *(feel free to insert an appropriate callsign for your local institution/area)*  After the learner acknowledges transmission:  “Medic 2 is en route to your facility with an 82-year-old female who had a mechanical fall and possible loss of consciousness. She has a history of atrial fibrillation. When we arrived on scene, her heart rate was in the 160s. We have given 10 mg of IV diltiazem and her heart rate is in the 140s. I am requesting permission to give an additional dose of diltiazem.”  All information below must be specifically requested by the learner | | |
| **Initial vital signs** | HR: 144 and irregular, BP: 116/80, Resp: 14, Sat: 97% on RA | | |
| **Complaints** | Headache, pain = 7/10. Palpitations. No chest pain or dyspnea | | |
| **Past Medical/Surgical History** | **Medications** | **Allergies** | **Family History** |
| Atrial fibrillation | Warfarin  Metoprolol | NKDA | hypertension  hyperlipidemia  coronary artery disease  atrial fibrillation |
| **Physical Examination** | | | |
| **General** | Awake, alert, oriented x4, and in no acute distress | | |
| **HEENT** | Normocephalic, contusion noted on right parietal scalp | | |
| **Neck** | Supple, full range of motion, no tenderness | | |
| **Lungs** | Non-labored breathing, clear to auscultation bilaterally | | |
| **Cardiovascular** | Normal S1/S2, irregularly irregular rhythm, tachycardic, no murmurs | | |
| **Abdomen** | Soft, non-distended, non-tender | | |
| **Extremities** | No signs of trauma, no deformities, grossly normal range of motion | | |
| **Neurological** | Alert, cranial nerves grossly intact, moving all extremities, normal sensation to touch in all 4 extremities, GCS 15 | | |
| **Skin** | Warm, dry, no obvious rash | | |
| **Psychiatric** | Answering questions appropriately with normal insight and judgement | | |

| Instructor Notes - Changes and CASE Branch Points | |
| --- | --- |
| **Intervention / Time point** | **Change in Case** |
| *Learner asks what treatment has been done* | - 20g IV left AC - Hung 1L bag of NS, approximately 100 mL has gone in - 12-lead ECG: A.fib, 144, no signs of ischemia - Diltiazem 10mg IV (as already mentioned) |
| *After learning that the patient has signs of head trauma* | **Critical Actions:**   - Denies request for an additional dose of diltiazem - Requests that the patient be transported to either Hospital B   If the details of the local trauma system were withheld to begin with, the facilitator should provide the trauma designation level and distance for Hospital B as information that the learner would already know |

**Ideal Scenario Flow**

After receiving the initial report and orders request from the prehospital provider, the learner recognizes the potential for trauma in a patient on anticoagulation due to the provided history of possible loss of consciousness and atrial fibrillation. He/she should ask the prehospital provider for more information to include vitals, medications, and focused elements of the physical exam. Upon learning that the patient does in fact have signs of head trauma while taking warfarin, he/she should recognize the potential for intracerebral hemorrhage and the need for the patient to be evaluated at a trauma center. He/she should deny the request for further diltiazem, as the tachycardia could be compensatory due to bleeding and/or early response to increased intracerebral pressure, and should request that the patient be taken to a trauma center rather than proceeding to the learner’s facility.

**Anticipated Management Mistakes**

1. Failure to obtain appropriate information. The facilitator may provide a one-time hint which suggests that the requested order may not be appropriate. For example: “I just wanted to be sure since she has this contusion on her head.”
2. Failure to deny the repeat dose of diltiazem: The case will conclude with whatever orders and transport decision are given. If the learner does not deny the additional dose of diltiazem, then at the beginning of the debrief, the facilitator should state that the patient was unstable on arrival with severe bradycardia.
3. Failure to divert to a trauma center: The case will conclude with whatever orders and transport decision are given. If the learner fails to divert the incoming ambulance to a trauma center, then at the beginning of the debrief, the facilitator should state that a CT scan obtained after the patient arrived at the learner’s facility showed an acute epidural hematoma and he/she had to arrange transfer to a trauma center. Before this transfer could occur, the patient became unstable and required intubation, measures to reduce ICP, and ionotropic support.
4. Failure to perform both critical actions. Combine the outcomes of #2 and #3 above.

Debrief materials

What were the initial clues that something other than atrial fibrillation with RVR was potentially the issue?

- Fall with possible loss of consciousness
- History of atrial fibrillation suggesting anticoagulant use

Why is it important to listen carefully to the report and ask for additional information if needed?

- The prehospital provider may not always give all important, relevant information
- They are usually busy taking care of the patient by themselves (while their partner drives) and calling report to the hospital and/or asking for orders at the same time. They want to give the minimum information necessary to get what they feel is needed.
- They, like anyone, are subject to anchoring bias, especially if busy with the patient and short on available time prior to arriving at your facility.

Why was giving additional diltiazem not an appropriate action?

- Not all tachycardic atrial fibrillation is atrial fibrillation with RVR.
- If there is concern for another issue causing a compensatory tachycardia, be careful using rate control agents before more information is available.

How did the patient qualify for trauma center evaluation?

- Yes. Step Four of the CDC Field Trauma Triage Guidelines includes head injury on anticoagulation
- Discuss the guidelines in more detail (provided below)
- It is important to know your local trauma destination guidelines, as your local center may use a modified version of these guidelines.

If more than one trauma center had been available, could the patient have been taken to a lower level trauma center that was closer?

- Per the CDC guidelines, yes.
- Anticoagulation puts patients at higher risk of intracerebral hemorrhage, but there still may be no significant injury.
- Lower level centers (i.e. level 3 and 4) do not have neurosurgery coverage, but they will have pre-arranged transfer agreements and should be equipped to initiate appropriate management. It may still be helpful to proactively contact the receiving hospital to discuss the potential need for neurosurgical intervention.

Discussion

As an emergency physician who provides online medical command, it is important to be familiar with your jurisdiction’s protocols regarding trauma center referral criteria. One of the most commonly used guidelines come from the CDC. The most recent algorithm developed by the CDC’s National Expert Panel on Field Triage is provided below.^1^ Step 1 assesses vital signs and level of consciousness. Step 2 evaluates anatomic injuries. Step 3 considers mechanism of injury. And Step 4 includes special considerations.

This particular patient highlights the importance of probing EMS providers for possible injuries that may necessitate trauma center referral. This patient, who had a history of atrial fibrillation, suffered a ground-level fall. This history of atrial fibrillation should clue emergency physicians to the potential that she may have been on anticoagulants. Asking for a physical exam would uncover a head contusion. Step 4 of the CDC guidelines specifically notes that patients with head trauma on anticoagulation should be considered for transport to a trauma center.

It is the position of the National Association of EMS Physicians that the Trauma Field Triage Guidelines be utilized by all EMS agencies.^2^

References

1. Sasser SM, Hunt RC, Faul M, et al. Guidelines for Field Triage of Injured Patients: Recommendations of the National Expert Panel on Field Triage, 2011*.* *MMWR* 2011;61:1-20.
2. NAEMSP, et al. Field triage of the injured patient. Prehosp Emerg Care 2011;15:541.
3. Chisholm KM, Harruff RC. Elderly deaths due to ground-level falls. Am J Forensic Med Pathol 2010;31:350–4.
4. Brewer ES, Reznikov B, Liberman RF, et al. Incidence and predictors of intracranial hemorrhage after minor head trauma in patients taking anticoagulant and antiplatelet medication. J Trauma 2011;70:E1–5.

2011 CDC Field Trauma Triage Guidelines

Step One: assess vital signs and level of consciousness

- Glascow Coma Score ≤ 13
- Systolic blood pressure < 90 mmHg
- Respiratory rate < 10 or > 29 (< 20 in infant aged < 1 year), or need for ventilatory support

Step Two: assess anotomy of injury

- All penetrating injuries to head, neck, torso, and extremities proximal to elbow or knee
- Chest wall instability or deformity (e.g. flail chest)
- Two or more proximal long bone fractures
- Crushed, degloved, mangled, or pulseless extremity
- Amputation proximal to wrist or ankle
- Pelvic fractures
- Open or depressed skull fractures
- Paralysis

**If any condition from Step One or Step Two is present, the patient should be transported to the highest level trauma center within the system

Step Three: Assess mechanism of injury and evidence of high-energy impact

- Falls
  - Adults: >20 feet (one story is equal to 10 feet)
  - Children: >10 feet or two or three times the height of the child
- High-risk auto crash
  - Intrusion, including roof: > 12 inches occupant site; > 18 inches any site
  - Ejection (partial or complete) from automobile
  - Death in same passenger compartment
  - Vehicle telemetry data consistent with a high risk of injury
- Auto vs. pedestrian/bicyclist thrown, run over, or with significant (> 20 mph) impact
- Motorcycle crash > 20 mph

**If any condition from Step Three is present, the patient should be transported to a trauma center, but it need not be the highest level center in the system

Step Four: Assess special patient or system considerations

- Older adults
  - Risk of injury/death increases after age 55 years
  - SBP < 110 might represent shock after age 65 years
  - Low impact mechanisms (e.g. ground level falls) might result in severe injury
- Children
  - Should be triaged preferentially to pediatric capable trauma centers
- Anticoagulants and bleeding disorders
  - Patients with head injury are at high risk for rapid deterioration
- Burns
  - Without other trauma mechanism: triage to burn facility
  - With trauma mechanism: triage to trauma center
- Pregnancy > 20 weeks
- EMS provider judgement

**If any condition from Step Four is present, the patient should be transported to a trauma center or any hospital capable of timely and through evaluation and initial management of potentially serious injuries. If no condition from any of Steps One through Four are present, transport based on local protocol.

| **Appendix A: OLMC Scenarios – Case 3**  **SIMULATION CASE TITLE: EMS OLMC – Major Trauma and Request for Aviation Resources**  **AUTHORS: Jose V. Nable, MD and Frank Tift, MD**  **LEARNER AUDIENCE: Emergency Medicine residents and medical students** | |
| --- | --- |
| **PATIENT NAME: N/A (patient names are not given over the radio)**  **PATIENT AGE: 24**  **CHIEF COMPLAINT: right leg pain post MVC**  **PHYSICAL SETTING: Learner is working in an Emergency Department that provides online medical control to EMS units when he/she is contacted (by radio) by an EMS unit requesting orders.** | |
|  | |
| **Brief narrative description of case** | In this scenario, a prehospital provider is requesting orders from the learner over a radio to launch air medical resources for a 24-year-old male who was an unrestrained passenger in a rollover MVC who required extrication. There was also a death within the same vehicle. Despite this mechanism and risk factors, objective signs from the patient do not suggest severe, unstable injury. A level 2 trauma center is closer to the unit by ground than the level 1 center is by air, and the learner should deny the request for air resources and recommend transport to the closer level 2 center by ground. |
| **Primary Learning Objectives** | By the end of this scenario, learners will be able to:   - Demonstrate proper use of the radio - Recognize the importance of asking for additional information - Utilize the CDC Field Trauma Triage Guidelines appropriately - Appropriately deny a request for unnecessary use of air medical resources |
| **Critical Actions** | 1. Utilizes the radio correctly 2. Asks the prehospital provider for vital signs and physical exam findings 3. Asks the prehospital provider for expected transport times 4. Denies the request for air medical resources |
| **Learner Preparation or Prework** | The learner an attending physician on duty at Hospital A, an academic, level 1 trauma center which provides OLMC services for the entire region. There is also a level 2 trauma center within the region, Hospital B, which is about 40 minutes away from Hospital A by car.  The learner should review the CDC Field Trauma Triage Guidelines prior to the session |

| Radio report | | | |
| --- | --- | --- | --- |
| **Overall Setting and Appearance** | No specific setting is required other than ensuring that the learner and facilitator are in separate rooms and able to communicate via a two-way radio | | |
| **Confederates (e.g., standardized participants) and their roles in the room at case start** | No additional personnel are required. | | |
| **Facilitator script for opening radio statement** | “Medic 3 to [OLMC]” *(feel free to insert an appropriate callsign for your local institution/area)*  After the learner acknowledges transmission:  “This is Medic 3 from [White County EMS]. I have a 24-year-old male that was an unrestrained backseat passenger in a rollover MVC which entrapped the patient. The driver was dead on arrival, and the patient required about 10 minutes to be extricated from the vehicle. I am requesting permission to launch air medical to transport him to your facility.”  *(Feel free to change the EMS service to an appropriate service in your area)*  All information below must be specifically requested by the learner  *(Learner may simply ask for traumatic findings and be given a summary of them)* | | |
| **Initial vital signs** | HR: 96, BP: 112/74, Resp: 14, Sat: 98% on RA | | |
| **Past Medical/Surgical History** | **Medications** | **Allergies** | **Family History** |
| None | None | NKDA | N/A |
| **Physical Examination** | | | |
| **General** | Awake, alert, oriented x4, and in moderate distress due to pain | | |
| **HEENT** | Normocephalic, contusion to right parietal scalp | | |
| **Neck** | Supple, full range of motion, no tenderness | | |
| **Lungs** | Non-labored breathing, clear to auscultation bilaterally, multiple abrasions to chest and back | | |
| **Cardiovascular** | Normal S1/S2, regular rhythm, no murmurs | | |
| **Abdomen** | Soft, non-distended, non-tender | | |
| **Extremities** | Obvious bony deformity of right femur at midshaft, no other signs of trauma | | |
| **Neurological** | Alert, cranial nerves grossly intact, moving all extremities, normal sensation to touch in all 4 extremities, GCS 15 | | |
| **Skin** | Warm, dry, no obvious rash | | |
| **Psychiatric** | Answering questions appropriately with normal insight and judgement | | |

| Instructor Notes - Changes and CASE Branch Points | |
| --- | --- |
| **Intervention / Time point** | **Change in Case** |
| *If learner asks for repeat vitals* | HR: 82, BP: 122/86, RR: 16, Sat: 99% on RA |
| *Learner asks what treatment has been given/performed* | - Spinal immobilization - Bilateral 18g IVs in both ACs - Fentanyl 100 mcg IV |
| *Learner asks for approximate transport times* | **Critical Action**:   - Requests a second set of vital signs for stability - Asks for approximate transport times   Hospital A by ground: 50 minutes  Hospital B by ground: 15 minutes  Hospital A by air: 15-20 minutes |
| *After learning vitals, physical exam findings, and transport times* | **Critical Action**:   - Deny request for air medical resources and recommend transport by ground to Hospital B |

**Ideal Scenario Flow**

After receiving the initial report and request for orders from the prehospital provider, the learner should recognize that more information is needed to determine whether air transport is necessary. He/she should ask for vital signs and traumatic exam findings. He/she should then recognize that the patient does not actually meet any Step One or Step Two criteria from the CDC Field Trauma Triage Guidelines, and thus does not require transport to the highest-level trauma center and may be evaluated at a lower level trauma center. He/she should then ask the crew about approximate transport times, and upon learning that Hospital B is closer by ground than Hospital A even by air, he/she should direct that the patient be taken to Hospital B by ground.

**Anticipated Management Mistakes**

1. Failure to obtain appropriate information. The facilitator may provide a one-time hint if the learner initially authorizes air transport. For example: “I just wanted to be sure that you didn’t think it should go to the other trauma center.”
2. Failure to obtain a second set of vital signs. The facilitator may provide a one-time hint if the learner does not reassess vital signs. For example: “I was worried he might become more unstable.”
3. Failure to deny air medical request. The scenario will end whether or not the request is granted. If granted, at the beginning of the debrief, the facilitator may mention that a few weeks later, the learner received a notification from the air medical service that they were reviewing the case as part of their standard utilization review and are requesting more information about the learner’s decision to authorize air transport.

Debrief materials

Did the patient qualify for trauma center evaluation?

- Yes. Step three of the CDC Field Trauma Triage Guidelines includes death in the same compartment as a high-risk mechanism of injury.
- Additionally, despite none of them meeting Step Two criteria, he has obvious injuries which warrant evaluation by an orthopedist, and those findings plus the high-risk mechanism further suggest the need for a trauma center.
- It is important to know your local trauma destination guidelines, as your local center may use a modified version of the CDC guidelines.

Did the patient need to go to the highest-level trauma center available?

- No, as none of his injuries nor his vital signs meet Step One or Step Two criteria
- Continue to discuss the guidelines in detail

When is air medical transport appropriate?

- Joint position statement defines clinical benefit from HEMS as:
  - Meaningfully shortening time to definitive care in time-sensitive conditions
  - Providing necessary specialized expertise or equipment
  - Providing transport to patients inaccessible by other means
- It is also generally considered appropriate if there are few enough local ground resources such that one leaving the area for multiple hours to transport would be detrimental to the local EMS system

Discussion

Inappropriate use of helicopter EMS (HEMS) is being increasingly recognized as a concern throughout the country. Overtriaging patients to use of aviation resources is expensive and can put providers, patients, and members of the public at risk from aircraft mishaps.^1,2^ Conversely, undertriage of patients increases morbidity and mortality.^3^ HEMS can provide critically-ill patients with significant technical expertise and speedier transport to definitive care, improving chances of survival.^4^

Emergency physicians who are called upon to authorize the use of HEMS must balance the need of providing high quality care to patients with these potential safety and cost concerns. Also, when ground transport times are relatively short, it may be better to transport such patients by conventional EMS rather than wait on-scene for HEMS. The time it takes for HEMS to be dispatched to the scene must be taken into account.

It is the position of the National Association of EMS Physicians that HEMS provides a clinical benefit by:^5^

1. Meaningfully shortening the time to delivery of definitive care to patients with time-sensitive medical conditions

2. Providing necessary specialized medical expertise or equipment to patients before and/or during transport

3. Providing transport to patients inaccessible by other means of transport

For this particular patient, who is relatively stable, it is much faster for the ground EMS providers to transport directly to another trauma center, rather than wait for aviation resources.

References

1. Bledsode BE, et al. Medical helicopter accidents in the United States: a 10-year review. *J Trauma* 2004;56:1325-8.
2. Taylor CB, et al. Helicopter Emergency Medical Services (HEMS) over-triage and the financial implications for major trauma centres in NSW, Australia. *BMC Emerg Med* 2013;13:11.
3. Haas B, et al. Survival of the fittest: the hidden cost of undertriage of major trauma. *J Am Coll Surg* 2010;211:804-811.
4. Galvagno SM, et al. Association between helicopter vs ground emergency medical services and survival for adults with major trauma. *JAMA* 2012;307:1602-1610.
5. Floccare DJ, Stuhlmiller DF, Braithwaite SA, et al. Appropriate and safe utilization of helicopter emergency medical services: a joint position statement with resource document. *Prehosp Emerg Care* 2013;17:521-5.

| **Appendix A: OLMC Scenarios – Case 4**  **SIMULATION CASE TITLE: EMS OLMC – Termination of Resuscitation**  **AUTHORS: Frank Tift, MD and Jose V. Nable, MD**  **LEARNER AUDIENCE: Emergency Medicine residents and medical students** | |
| --- | --- |
| **PATIENT NAME: N/A (patient names are not given over the radio)**  **PATIENT AGE: mid 50s**  **CHIEF COMPLAINT: cardiac arrest**  **PHYSICAL SETTING: Learner is working in an Emergency Department that provides online medical control to EMS units when he/she is contacted (by radio) by an EMS unit requesting orders.** | |
|  | |
| **Brief narrative description of case** | In this scenario, a prehospital provider is requesting orders from the learner over a radio to terminate resuscitation for a case of ongoing CPR. After requesting more information from the prehospital providers, it is clear that this patient does not meet criteria for field termination of resuscitation (TOR), as he had a shockable rhythm on arrival and was defibrillated. The learner should then direct the prehospital provider to continue with resuscitation and transport to the hospital. |
| **Primary Learning Objectives** | By the end of this scenario, learners will be able to:   - Demonstrate proper use of the radio - Recognize the importance of asking for additional information - Demonstrate proper application of the “TOR” rule. |
| **Critical Actions** | 1. Utilizes the radio correctly 2. Asks the prehospital provider for more information about resuscitative efforts 3. Recognizes that the patient does not meet criteria for field TOR 4. Denies the request for TOR and recommends transport to the hospital |
| **Learner Preparation or Prework** | The learner an attending physician on duty in a double coverage ED in a community hospital which is a STEMI center with ICU coverage.  The learner should be familiar with the “BLS TOR” rule prior to the session (see below)  Reading reference number 1 below is strongly recommended prior to the session. |

| Radio report | | | |
| --- | --- | --- | --- |
| **Overall Setting and Appearance** | No specific setting is required other than ensuring that the learner and facilitator are in separate rooms and able to communicate via a two-way radio | | |
| **Confederates (e.g., standardized participants) and their roles in the room at case start** | No additional personnel are required. | | |
| **Facilitator script for opening radio statement** | “Medic 4 to [OLMC]” *(feel free to insert an appropriate callsign for your local institution/area)*  After the learner acknowledges transmission:  Medic 4 is on scene with a CPR in progress. The patient is a male and appears to be in his mid-50s. He has been asystole on the monitor for the last 4 pulse checks. We are requesting permission to terminate resuscitation”  All information below must be specifically requested by the learner | | |
| **Initial vital signs** | Asystole. Receiving rescue breaths by BVM via ET tube at approximately 10-12 times per minute | | |
| **Past Medical/Surgical History** | **Medications** | **Allergies** | **Family History** |
| (on scene family can provide if asked)  hypertension  coronary artery disease  Prior heart attack | Unknown | (on scene family can provide if asked)  NKDA | (on scene family can provide if asked)  Coronary artery disease  Heart attacks |
| **Physical Examination** | | | |
| **General** | Obtunded, moribund | | |
| **HEENT** | Normocephalic, atraumatic | | |
| **Neck** | No stiffness, no deformity. 8.0 ETT at 25 cm at the lips | | |
| **Lungs** | Assisted by BVM. Lungs clear to auscultation bilaterally | | |
| **Cardiovascular** | Asystole | | |
| **Abdomen** | Soft, non-distended, non-tender | | |
| **Neurological** | Unresponsive. No movement to pain in any extremity | | |
| **Skin** | Pale and cool | | |
| **GU** | *(Not examined)* | | |
| **Psychiatric** | Unresponsive | | |

| Instructor Notes - Changes and CASE Branch Points | |
| --- | --- |
| **Intervention / Time point** | **Change in Case** |
| *Learner asks for the presenting rhythm* | *Ventricular tachycardia which converted to asystole after a single defibrillation attempt* |
| *Learner requests a summary of treatment so far* | “We arrived to find bystanders performing CPR. Presenting rhythm was ventricular tachycardia, so we shocked him after attaching the monitor, and his rhythm converted to asystole. We intubated him and have performed 4 rounds of CPR with two doses of epinephrine given and one of bicarb. Rhythm has been asystole at all pulse checks. We have been coding him for maybe 10-15 minutes.” |
| *After learning the initial rhythm and that defibrillation was attempted* | **Critical Actions**:   - Deny request for TOR - Request transport to the hospital with continued resuscitative efforts |

**Ideal Scenario Flow**

After receiving the initial report and request for orders from the prehospital provider, the learner should recognize that more information is needed to determine field termination of resuscitation (TOR) is appropriate. He/she should request the initial rhythm as well as further summary of what resuscitative efforts have occurred. Upon learning that the patient’s initial rhythm was ventricular tachycardia and he received defibrillation, the learner should recognize that the patient does not meet field TOR criteria, should deny the request to terminate, and recommend transport to the hospital with continued resuscitative efforts.

**Anticipated Management Mistakes**

1. Failure to obtain needed information. The facilitator may provide a one-time hint that further details are available. For example; “Okay, we just weren’t sure after the defibrillation.”
2. Failure to deny TOR request and recommend transport. The prehospital providers will come back over the radio and state that the family is strongly against ceasing resuscitative efforts and are becoming agitated and aggressive towards the crew. They are planning to continue resuscitation and transport due to safety concerns.

Debrief materials

Why did the patient not meet TOR criteria?

- The “BLS TOR” rule states that termination of resuscitation is appropriate if all of the following are true:
  - Arrest was not witnessed by EMS personnel
  - No shock was delivered
  - No return of spontaneous circulation prior to initiating transport
- This patient’s initial rhythm was shockable and defibrillation was attempted

Why is field TOR currently recommended?

- Low likelihood of meaningful survival in out of hospital cardiac arrest
- CPR performed on scene has been shown to be higher quality than CPR performed during transport, thus there is a higher chance of successful resuscitation if the crew stays on scene to resuscitate, but field TOR and non-transport must be an option to allow this practice
- Reduce risk of injury to patient, EMS crew, and the general public from traffic accidents related to driving emergency traffic
- Increase availability of local EMS resources by allowing them to return to service sooner
- Decrease unnecessary use of ED resources

Are there other field TOR rules, and how would they apply to this patient?

- Yes, several.^1^
- Many include ALS measures, but none have had significantly better predictive value than the BLS TOR rule
- Some rules include no bystander CPR as a criterion, which this patient received.
- Many state that resuscitation should be attempted for more than 15-20 minutes before TOR is appropriate, and this patient has received resuscitation for approximately 10-15 minutes
- It is important to be familiar with the TOR guidelines for your local EMS services

Why would the medics not include the presenting rhythm in their initial report?

- May simply have forgotten given the stress of the situation
- May feel that successful revival is unlikely and prefer to terminate given all of the reasons above that TOR is currently recommended in appropriate situations

Would asking the crew to continue efforts on scene for another 10-15 minutes then call back for TOR orders be an option?

- Depends on local guidelines
- Insufficient evidence to recommend this practice, but it is done in some areas for all of the same reasons that TOR is recommended in appropriate situations
- Again, it is important to be familiar with your local EMS service guidelines.

Discussion

It is the position of the National Association of EMS Physicians that all EMS agencies should have protocols allowing for termination of resuscitation (TOR) in the field.^1^ A TOR protocol allows providers to concentrate on providing high quality CPR in the field, rather than transporting all cardiac arrests to the emergency department. Transporting patients has been associated with poorer chest compressions, with chest compression quality being an important determinant in survival from cardiac arrest.^2,3^

Several guidelines have been developed for field TOR protocols.^1,4,5^ While more research is needed to develop evidence-based guidelines, it is generally believed that presence of a shockable rhythm at any point during resuscitation should be considered a contraindication for field TOR.^1^

References:

1. Millin MG, Khandker SR, Malki A. Termination of resuscitation of nontraumatic cardiopulmonary arrest: resource document for the National Association of EMS Physicians position statement. *Prehosp Emerg Care* 2011;15:547.
2. Russi CS, Kolb LJ, Myers LA. A comparison of chest compression quality delivered during on-scene and transport cardiopulmonary resuscitation [abstract]. *Prehosp Emerg Care* 2011;15: 106.
3. Christenson J, Andrusiek D, Everson-Stewart S, et al. Chest compression fraction determines survival in patients with out-of-hospital ventricular fibrillation. *Circulation* 2009;120:1241-7.
4. Morrison LJ, Verbeek PR, Vermeulen MJ, et al. Derivation and evaluation of a termination of resuscitation clinical prediction rule for advanced life support providers. Resuscitation 74: 266, 2007.
5. Sherbino J, Leim SM, Davis DP, et al. Clinical decision rules for termination of resuscitation in out-of hospital cardiac arrest. *J Emerg Med* 2010;38:80.
6. Van den Hoek TL,Morrison LJ, ShusterM, et al. Part 12: cardiac arrest in special situations: 2010 American Heart Association guidelines for cardiopulmonary resuscitation and emergency cardiovascular care. *Circulation* 2010;18(suppl 3):S829–S861.

| **Appendix A: OLMC Scenarios – Case 5**  **SIMULATION CASE TITLE: EMS OLMC – Pediatric Refusal of Care**  **AUTHORS: Jose V. Nable, MD and Frank Tift, MD**  **LEARNER AUDIENCE: Emergency Medicine residents and medical students** | |
| --- | --- |
| **PATIENT NAME: N/A (patient names are not given over the radio)**  **PATIENT AGE: 15**  **CHIEF COMPLAINT: shortness of breath**  **PHYSICAL SETTING: Learner is working in an Emergency Department that provides online medical control to EMS units when he/she is contacted (by radio) by an EMS unit requesting orders.** | |
|  | |
| **Brief narrative description of case** | In this scenario, a prehospital provider is requesting orders from the learner over a radio to allow a patient to refuse further care and not be transported to the hospital. However, this patient is a minor, and thus he is not legally allowed to refuse care. His parents are not available to make decisions, thus the learner should deny the prehospital provider’s request and instruct that the patient be brought to the emergency department. |
| **Primary Learning Objectives** | By the end of this scenario, learners will be able to:   - Demonstrate proper use of the radio - Discuss the limitations of legal decision making with regard to minor patients - Demonstrate the ability to appropriately manage a situation where a pediatric patient does not wish to be treated. |
| **Critical Actions** | 1. Utilizes the radio correctly 2. Recognizes that the patient is a minor, and thus cannot legally refuse care 3. Asks whether the patient’s parents are available for decision making 4. Denies the request to allow refusal and instructs the prehospital provider that the patient must be transported to the emergency department 5. Engages law enforcement to assist when necessary |
| **Learner Preparation or Prework** | The learner is an attending physician on duty in a double-coverage community emergency department in a small town, which is the only hospital in town. This hospital has basic Internal Medicine and Pediatrics coverage for routine issues.  The learner should be familiar with the laws surrounding pediatric consent for treatment and refusal of care. Reading reference 3 below is strongly recommended. |

| Radio report | | | |
| --- | --- | --- | --- |
| **Overall Setting and Appearance** | No specific setting is required other than ensuring that the learner and facilitator are in separate rooms and able to communicate via a two-way radio | | |
| **Confederates (e.g., standardized participants) and their roles in the room at case start** | No additional personnel are required. | | |
| **Facilitator script for opening radio statement** | “Medic 5 to [OLMC]” *(feel free to insert an appropriate callsign for your local institution/area)*  After the learner acknowledges transmission:  “This is Medic 5. I’m on scene with a 15-year-old male who is having an asthma attack. He used his own inhaler prior to our arrival, and he states he feels a little better. He still has some wheezing on exam, but he states he does not wish to go to the ER. I am requesting permission to obtain a patient refusal.”  All information below must be specifically requested by the learner | | |
| **Initial vital signs** | HR: 108, BP: 114/84, Resp: 20, Sats: 96% on RA | | |
| **Past Medical/Surgical History** | **Medications** | **Allergies** | **Family History** |
| Asthma | Albuterol as needed | NKDA | asthma |
| **Physical Examination** | | | |
| **General** | Awake, alert, and in no acute distress | | |
| **HEENT** | Normocephalic, atraumatic | | |
| **Neck** | Supple, full range of motion, no tenderness | | |
| **Lungs** | Non-labored breathing, mild diffuse expiratory wheeze | | |
| **Cardiovascular** | Normal S1/S2, regular rhythm, no murmurs | | |
| **Abdomen** | Soft, non-distended, non-tender | | |
| **Neurological** | Alert, cranial nerves grossly intact, no gross focal deficits | | |
| **Skin** | Warm, dry, no obvious rash | | |
| **GU** | *(Not examined)* | | |
| **Psychiatric** | Answering questions appropriately with normal insight and judgement | | |

| Instructor Notes - Changes and CASE Branch Points | |
| --- | --- |
| **Intervention / Time point** | **Change in Case** |
| *Immediately after report* | **Critical Actions**:   - Recognizes that the patient is a minor and may not refuse - Asks whether the patient’s parent/guardian is available   After asking for parent/guardian:  “No. He is attending a concert in the park with some friends. His parents are not with him.” |
| *After learning that the patient’s parents are not available* | Learner should ask whether there is any way to contact the patient’s parents. (not a critical action) If he/she does so:  “No. He says they are out of town, and he is staying with his friend. He has already tried to call them, and they did not answer.” |
| *After learning that the patient’s parents are unavailable and cannot be contacted.* | **Critical Action:**   - Denies request for refusal and instructs the prehospital providers to transport the patient to the ER |
| *After instructing prehospital providers to transport the patient* | “Sir/Ma’am, he has become rather angry and is yelling at us that he will not go and that he is going to sue us. What should we do?”  **Critical Action:**   - Engage law enforcement   The learner should tell the prehospital providers to engage law enforcement to de-escalate the situation and assist in getting the patient to come to the hospital. They could either ask about law enforcement already on scene for the concert or ask for police to be dispatched to the scene. If done, the patient will calm down and agree to go to the hospital. |

**Ideal Scenario Flow**

After receiving the initial report and request for orders, the learner should recognize that the patient is a minor and is not legally allowed to refuse transport. The learner should then attempt to engage the patient’s parents to make the decision, at which point he/she learns that they are not present. He/she should ideally make further attempts to contact them as well. When it is clear that the parents are unavailable to make decisions, the learner should instruct the prehospital providers to transport the patient to the ED. The patient then becomes agitated towards the crew and continues to refuse to go to the hospital, at which point law enforcement should be engaged to assist with de-escalation and transport.

**Anticipated Management Mistakes**

1. Failure to deny the request for refusal. The scenario will end, but at the beginning of the debrief, the facilitator can tell the learner that the EMS service forwarded a complaint from the patient’s mother to the ED who is very angry and stating that her son should have been evaluated and treated. She is threatening to sue for patient abandonment.
2. Failure to attempt to contact the patient’s parents. The scenario will end, but at the beginning of the debrief, the facilitator can tell the learner that the EMS service forwarded a complaint from the patient’s mother to the ED who is stating that she feels she should have been contacted before her son was taken to the ED. She is refusing to pay the bills for either the ambulance service or the ED.
3. Failure to engage law enforcement. This situation can be handled in two ways. First, the patient is brought into the ED on the stretcher with physical restraints in place and with worsening respiratory distress. The scenario will end, and the facilitator can tell the learner that multiple complaints were filed with the EMS service from bystanders who witnessed EMS restrain the child. His mother is also threatening to sue for assault and battery. Alternatively, the prehospital provider may call back over the radio and state that the patient has fled the scene and inform the learner that they contacted police, who are now looking for the child, and that they are returning to service.
4. If the learner tells EMS to sedate/restrain the patient. This situation can be handled just as the first option above.

Debrief materials

What are the rules about minors consenting for or refusing treatment and/or transport?

- They are not allowed to make decisions about their own care unless they are an emancipated minor, married, or the medical issue involves reproductive health (pregnancy/STDs)
- A parent or legal guardian must give consent for treatment and/or transport.

What may be done for a minor patient without consent?

- Providers may treat obvious life-threatening conditions under implied consent
- A minor may be transported to the ED against his/her will if parental refusal is not available
- Treatment for non-life-threatening conditions should be withheld until parent/guarding consent is obtained. As such, EMS transport without further treatment is an option.

How could consent be obtained in this situation?

- By phone is the most plausible way.
- You must have reasonable certainty that the person on the other end of the line is in fact the patient’s parent or guardian.
- Should have two separate people discuss with the parent/guardian over the phone and document the conversation and which two people confirmed consent.
- Should ideally still get consent in writing after the fact if able

What if the patient continues to refuse?

- EMS crews and ED providers should do what is in the best interest of the patient, but they should also not compromise their own safety or the safety of the patient.
- Physically or chemically restraining the patient is inherently dangerous and should be avoided if at all possible
- Law enforcement should be involved and can assist with proper restraints if needed, though all efforts should be made to convince the patient to go willingly and without restraints.
- EMS and ED providers should attempt to prevent the patient from fleeing if able to do so safely, but they do not need to put themselves at risk of harm or pursue a fleeing patient due to safety concerns. They should report the situation to law enforcement.

Discussion

Patients not uncommonly refuse transport by EMS.^1^ Many jurisdictions require online medical command consultation in order for EMS providers to obtain a patient-initiated refusal. Non-transports are a source of significant litigation, so emergency physicians must have a thorough understanding of their potential role in such situations.

Prehospital providers are challenged when parents are not on scene with a child having a medical emergency.^3^ They should make an attempt to contact the patient’s parent or guardian to secure consent to treat, but they may transport a minor patient against his/her will if no parent or guardian is available. Treatment of life-threatening conditions is allowed by implied consent, but all other treatment should be withheld until consent is obtained. In difficult situations surrounding minor consent, law enforcement should be involved.

References:

1. Knight S, Olson LM, Cook LJ, et al. Against all advice: an analysis of out-of-hospital refusals of care. *Ann Emerg Med* 2003;42:689-96.
2. Stuhlmiller DF, Cudnik MT, Sundheim SM, et al. Adequacy of online medical command communication and emergency medical services documentation of informed refusals. *Acad Emerg Med* 2005;12:970-7.
3. Committee on Pediatric Emergency Medicine and Committee on Bioethics. Consent for emergency medical services for children and adolescents. *Pediatrics* 2011;128:427-33.

| **Appendix A: OLMC Scenarios – Case 6**  **SIMULATION CASE TITLE: EMS OLMC – Atypical STEMI**  **AUTHORS: Jose V. Nable, MD and Frank Tift, MD**  **LEARNER AUDIENCE: Emergency Medicine residents and medical students** | |
| --- | --- |
| **PATIENT NAME: N/A (patient names are not given over the radio)**  **PATIENT AGE: 52**  **CHIEF COMPLAINT: shortness of breath**  **PHYSICAL SETTING: Learner is working in a freestanding Emergency Department that provides online medical control to EMS units when he/she is contacted (by radio) by an EMS unit requesting orders.** | |
|  | |
| **Brief narrative description of case** | In this scenario, a prehospital provider is providing a “heads up” to the learner about a sick patient that he is bringing to the learner’s facility. The provider believes the patient to be suffering from a CHF exacerbation, but upon request for an ECG, it is determined that the patient is having an inferior STEMI. The learner’s facility is not a PCI-capable facility, while there is one nearby. The learner should divert the EMS unit to the PCI-capable facility. |
| **Primary Learning Objectives** | By the end of this scenario, learners will be able to:   - Demonstrate proper use of the radio - Recognize the importance of asking for additional information - Discuss the EMTALA law and how it applies to diverting an ambulance away from your facility. |
| **Critical Actions** | 1. Utilizes the radio correctly 2. Asks the prehospital provider whether an ECG has been performed 3. Diverts the ambulance to a more appropriate receiving facility |
| **Learner Preparation or Prework** | The learner is an attending physician on duty in a freestanding emergency department which accepts EMS traffic. There is a hospital in a nearby town, Hospital B, which is a STEMI center and a stroke center.  No specific additional preparation is required  (information about Hospital B may optionally be withheld until the learner asks, at which point it is presented as information that the learner would already know) |

| Radio report | | | |
| --- | --- | --- | --- |
| **Overall Setting and Appearance** | No specific setting is required other than ensuring that the learner and facilitator are in separate rooms and able to communicate via a two-way radio | | |
| **Confederates (e.g., standardized participants) and their roles in the room at case start** | No additional personnel are required. | | |
| **Facilitator script for opening radio statement** | “Medic 6 to [OLMC]” *(feel free to insert an appropriate callsign for your local institution/area)*  After the learner acknowledges transmission:  “Medic 6 is en route to your facility with a 52 year-old-male with shortness of breath. I’ve placed him on CPAP because I heard crackles bilaterally. I’m just giving you a heads up so you can prepare.”  All information below must be specifically requested by the learner | | |
| **Initial vital signs** | HR: 68, BP: 145/62, Resp: 18, Sats: 100% on wall CPAP at a PEEP of 5 | | |
| **Past Medical/Surgical History** | **Medications** | **Allergies** | **Family History** |
| hypertension  diabetes | hydrochlorothiazide  metformin | NKDA | congestive heart failure  heart attacks  hypertension  diabetes |
| **Physical Examination** | | | |
| **General** | Awake, alert, in mild respiratory distress on CPAP mask | | |
| **HEENT** | Normocephalic, atraumatic | | |
| **Neck** | Supple, full range of motion, no tenderness | | |
| **Lungs** | Mild distress on CPAP, diffuse mild rales | | |
| **Cardiovascular** | Normal S1/S2, regular rhythm, no murmurs | | |
| **Abdomen** | Soft, non-distended, non-tender | | |
| **Neurological** | Alert, cranial nerves grossly intact, no gross focal deficits | | |
| **Skin** | Warm, diaphoretic, no obvious rash | | |
| **GU** | *(Not examined)* | | |
| **Psychiatric** | Answering questions appropriately with normal insight and judgement | | |

| Instructor Notes - Changes and CASE Branch Points | |
| --- | --- |
| **Intervention / Time point** | **Change in Case** |
| *Immediately after initial report* | **Critical Action:**   - Ask for ECG findings   Prehospital provider will reply:  “I haven’t gotten one. Do you want me to get one?”  Learner should say yes, then:  Prehospital provider says: “Oh! It’s showing ST elevation in leads II, III, and aVF. Do you have any further orders?” |
| *Other symptoms of MI and symptom onset* | If asked, the patient does not have chest pain, but he is vaguely nauseated and appears diaphoretic.  Onset immediately prior to calling 911, approximately 20 minutes before radio report. |
| *Medications given/orders* | If learner asks what meds have been given, the prehospital provider states that they have only started CPAP.  The learner may ask them to administer aspirin.  If he/she asks for heparin to be given, the prehospital provider should respond saying that he is not a critical care medic and they do not carry heparin on their truck. |
| *Transport times if/when asked* | To learner’s facility: 5 minutes  To Hospital B: 20 minutes |
| *After learning transport times* | **Critical Action:**   - Divert ambulance to Hospital B |

**Ideal Scenario Flow**

After receiving the initial report, the learner should recognize that shortness of breath could be an atypical presentation of a myocardial infarction, especially since this patient doesn’t have known history of CHF. He/she should ask whether a 12-lead ECG has been performed. Upon learning than it has not, the learner should request one. This ECG reveals an anterior STEMI. The learner should then realize that the patient would be better managed at PCI-capable facility and ask about transport times to Hospital B. Once it is clear that Hospital B is not significantly farther away, the learner should divert the ambulance to Hospital B.

**Anticipated Management Mistakes**

1. Failure to ask for an ECG. The facilitator may state that the patient has started to develop chest pain. If the learner still does not request an ECG, then the scenario will end with the patient arriving at the learner’s facility and the STEMI being discovered once an ECG is performed. Treatment options at that point may be discussed as part of the debrief.
2. Failure to divert the ambulance. The scenario will end with the patient arriving at the learner’s facility and the STEMI being discovered once an ECG is performed. Treatment options at that point will again be discussed in the debrief.

Debrief materials

What is EMTALA and how does it apply to freestanding EDs as well as EMS traffic coming to an ED?

- Emergency Medical Treatment and Active Labor Act
- EDs must perform a medical screening examination and appropriate stabilizing treatment to all patients who present to the ED requesting medical care
- Applies to freestanding EDs the same as an in-hospital ED
- Radio contact with the hospital does NOT incur an EMTALA obligation to the patient
- Facilities may divert an ambulance prior to arrival if they do not feel that can adequately care for the patient and/or it is in the patient’s best interest to go straight to a specialty center

When is it okay to divert an ambulance?

- If the facility does not have the capability to care for the patient, either due to overcrowding or lack of necessary service lines
- It helps if a formal diversion status has already been announced
- Any ED should be capable of stabilizing treatment, and such treatment should be provided if the patient comes to the ED despite a request to divert

What if Hospital B were significantly farther away?

- Consider air medical. Launching the helicopter before arrival would speed up the transfer process. The ambulance could even rendezvous with the helicopter without coming to the freestanding ED, but given how close they are and the time to launch a helicopter, it would be best for treatment to be initiated at the freestanding ED
- Consider giving tPA prior to transfer. Goal time for PCI is 90 minutes from first medical contact to balloon.^1^ EMS contact counts as first medical contact, so if the transfer process would lead to PCI >90 minutes after EMS contact, tPA should be given
- First medical contact to PCI within 90 minutes is a big reason why the emphasis has been placed on taking patients directly to STEMI centers rather than stopping at a closer facility.

What if the patient arrives at the freestanding ED anyway?

- Same as above
- Treat to the best of your ability

Discussion

Freestanding emergency departments (FEDs) are now being utilized throughout much of the healthcare system.^2^ Unlike emergency departments physically located within hospitals, FEDs in general must transfer patients for inpatient admission or for complex procedures.

FEDs can provide crucial interventions for unstable patients before being transferred to definitive acute care facilities. For example, a patient with an unstable airway might first be intubated at an FED. Indeed, EMS protocols may allow for EMS providers to first take their unstable patient to an FED.^3^

Determining whether to send a patient to a FED versus a traditional emergency department therefore requires balancing several concerns such as the acuity and predicted needs of the patient, along with distances to the FED versus a hospital. This particular patient was having a STEMI. In rural areas far from cardiac interventional centers or helicopter EMS resources, it may be reasonable to transport to an FED. However, in a more urban environment, directing this patient to the closest cardiac-capable facility would be most appropriate.

This case also requires online medical physicians to understand that STEMI can present other than typical chest pain.

References:

1. American Heart Association. Recommendations for Criteria for STEMI Systems of Care. <http://www.heart.org/HEARTORG/HealthcareProfessional/Mission-Lifeline-Recommendations-for-Criteria-for-STEMI-Systems-of-Care_UCM_312070_Article.jsp#.XLjjCOhKiUk> Accessed 18 April 2019.
2. Sullivan AF, Bachireddy C, Steptoe AP, Oldfield J, Wilson T, Camargo C a. A profile of freestanding emergency departments in the United States, 2007. *J Emerg Med* 2012;43:1175-80.
3. Maryland Institute for Emergency Medical Services. The Maryland Medical Protocols for Emergency Medical Services Providers. 2010.
4. O'Gara PT, Kushner FG, Ascheim DD, et al. 2013 ACCF/AHA guideline for the management of ST-elevation myocardial infarction: a report of the American College of Cardiology Foundation/American Heart Association Task Force on Practice Guidelines. J *Am Coll Cardiol* 2013 29;61:e78-140

| **Appendix A: OLMC Scenarios – Case 7**  **SIMULATION CASE TITLE: EMS OLMC – Adult Refusal of Care**  **AUTHORS: Frank Tift, MD and Jose V. Nable, MD**  **LEARNER AUDIENCE: Emergency Medicine residents and medical students** | |
| --- | --- |
| **PATIENT NAME: N/A (patient names are not given over the radio)**  **PATIENT AGE: 47**  **CHIEF COMPLAINT: hypoglycemia**  **PHYSICAL SETTING: Learner is working in an Emergency Department that provides online medical control to EMS units when he/she is contacted (by radio) by an EMS unit requesting orders.** | |
|  | |
| **Brief narrative description of case** | In this scenario, a prehospital provider is requesting orders from the learner over a radio to allow a patient to refuse care. This patient is a diabetic who had altered mental status due to hypoglycemia. After receiving dextrose, he is awake and oriented and does not wish to go to the hospital. The learner must determine if the patient has decision making capacity, which he does, then grant permission for refusal. |
| **Primary Learning Objectives** | By the end of this scenario, learners will be able to:   - Demonstrate proper use of the radio - Recognize the importance of asking for additional information - Discuss how to determine whether a patient has decision making capacity - Discuss when a patient may refuse medical care |
| **Critical Actions** | 1. Utilizes the radio correctly 2. Ensures the patient has decision-making capacity 3. Ensures the patient understands the risks of refusal 4. Allows the patient to refuse care |
| **Learner Preparation or Prework** | The learner is an attending physician on duty in the ED at a local community hospital.  No specific additional preparation is required. |

| Radio report | | | |
| --- | --- | --- | --- |
| **Overall Setting and Appearance** | No specific setting is required other than ensuring that the learner and facilitator are in separate rooms and able to communicate via a two-way radio | | |
| **Confederates (e.g., standardized participants) and their roles in the room at case start** | No additional personnel are required. | | |
| **Facilitator script for opening radio statement** | “Medic 7 to [OLMC]” *(feel free to insert an appropriate callsign for your local institution/area)*  After the learner acknowledges transmission:  “This is Medic 7. I’m on scene with a 47-year-old male with diabetes who had a hypoglycemic episode. Initial blood sugar was 27, but after an amp of D50, it’s up to 148. The patient is alert and oriented and does not wish to come to the hospital. However, this has not happened to him in several years, and he seems mildly concerned about starting a new medication, so I wanted to call for permission to allow the patient to refuse.”  All information below must be specifically requested by the learner | | |
| **Initial vital signs** | HR: 86, BP: 138/76, Resp: 12, Sats: 99% on RA | | |
| **Past Medical/Surgical History** | **Medications** | **Allergies** | **Family History** |
| hypertension  diabetes | lisinopril  metformin  glyburide | NKDA | hypertension |
| **Physical Examination** | | | |
| **General** | Awake, alert, and in no acute distress | | |
| **HEENT** | Normocephalic, atraumatic | | |
| **Neck** | Supple, full range of motion, no tenderness | | |
| **Lungs** | Non-labored breathing, clear to auscultation bilaterally | | |
| **Cardiovascular** | Normal S1/S2, regular rhythm, no murmurs | | |
| **Abdomen** | Soft, non-distended, non-tender | | |
| **Neurological** | Alert, cranial nerves intact, normal sensation, moving all extremities | | |
| **Skin** | Warm, dry, no obvious rash | | |
| **GU** | *(Not examined)* | | |
| **Psychiatric** | Answering questions appropriately with normal insight and judgement | | |

| Instructor Notes - Changes and CASE Branch Points | |
| --- | --- |
| **Intervention / Time point** | **Change in Case** |
| *Immediately following report* | The learner should take the hint about a new medication as important and ask about the patient’s medications. |
| *After learning the patient is on Glyburide* | Critical Action:   - Ensure patient understands the risks of refusal   The learner should recognize the risk of recurrent hypoglycemia and instruct the prehospital provider to ensure that the patient is:   - aware of that risk - understands that his blood sugar could drop again and be potentially fatal without medical treatment - understands that transport to the hospital is recommended. |
| *After determining that the patient understands the risks of refusal* | Critical Action:   - Learner grants permission to refuse |
| *Other instructions to EMS regarding hypoglycemia* | The learner could either instruct EMS to ensure the patient eats something or that a responsible person is present or both |
| Learner requests a repeat blood glucose | It is 125 |
| *Learner requests to speak to the patient* | The facilitator may assume the role of the patient talking to the learner over the radio. The patient should understand the risks of refusal after the discussion and still state that he does not wish to go to the hospital. |

**Ideal Scenario Flow**

After receiving report and the request for refusal, the learner should note that the prehospital provider seems a bit concerned about this patient despite this issue being routine. The learner should probe for more information, specifically regarding the new medication that the patient is taking. After hearing the patient’s medications, the learner will surmise that the Glyburide was likely the recently started agent. He/she should realize that this is a long acting hypoglycemic medication, putting the patient at higher risk of recurrent hypoglycemia. However, the patient is still within his right to refuse care assuming he has full decision-making capacity. That involved both being fully oriented AND understanding the risks of refusal, so the learner should ensure that the patient does understand those risks. Once so determined, the patient may be allowed to refuse care.

**Anticipated Management Mistakes**

1. Learner allows refusal without further information or without informing the patient of his risk of recurrent hypoglycemia. The case will end. At the beginning of the debrief, the facilitator may tell the learner that the patient presents to his/her ED by EMS about 2 hours later after an episode of recurrent hypoglycemia. He is angry that he was not informed that his new medicine put him at risk for recurrent hypoglycemia.
2. Failure to ask for medications. If the learner did an adequate job of ensuring the patient understood the risks of refusal even without determining medications, then the case will end and can be considered successful, but the additional details should be discussed in the debrief to further highlight the importance of the additional information

Debrief materials

When may a patient refuse medical care?

- Alert and oriented to person, place, time, and situation AND understand the risks of refusal
- It is often incorrectly believed that the patient merely needs to be fully oriented.
- This concept is an important part of medical ethics, as the patient who has decision-making capacity should be allowed to decide what care is rendered. (principle of autonomy)

When can a patient be transported against his/her will?

- Danger to self
- Danger to others
- Lacks decision-making capacity
  - Disoriented
  - If the patient appears oriented, but does not seem to understand the situation, then every attempt should be made to explain the risks. If the patient still does not seem to understand, then he/she is not fully informed and cannot make an appropriate medical decision.

What if the patient does not have decision-making capacity but refuses to go?

- Do your best to verbally explain the situation and convince the patient to go
- Engage law enforcement if the patient begins to get aggressive or violent.

Discussion

Patient refusals are a common issue in EMS, and they carry a large degree of liability for the EMS service and the service medical director. There is a wide variance in the industry as to whether a particular EMS service has a written policy regarding refusal of care, as well as what information is required to determine whether the patient may appropriately refuse^1^. In general, the best standard to determine capacity to refuse involves that the patient not only be fully oriented, but also understand the risks and benefits of refusal of care^1^.

This scenario highlights the importance of ensuring that the patient fully understands the risks of refusing care. In some cases, the physician him- or herself needs to obtain more information before the risks are truly known. This process can be particularly challenging when providing online medical control, as the information is relayed via the EMS provider on scene. Once certain situations become routine (such as reversal of hypoglycemia), the medical control physician should pay attention to the small clues which can indicate that this particular case is not so routine. In this example, a new episode of hypoglycemia in a patient that was previously well controlled, and the patient’s mention of a new medication warrant further investigation. Also keep in mind that it is certainly acceptable to request to speak directly to the patient if he/she is willing.

References:

1. Weaver J, Brinsfield KH, Dalphond D. Prehospital refusal-of-transport policies: adequate legal protection? Prehosp Emerg Care. 2000;4(1):53-6.

| **Appendix A: OLMC Scenarios – Case 8**  **SIMULATION CASE TITLE: EMS OLMC – Trauma Clearance**  **AUTHORS: Frank Tift, MD and Jose V. Nable, MD**  **LEARNER AUDIENCE: Emergency Medicine residents and medical students** | |
| --- | --- |
| **PATIENT NAME: N/A (patient names are not given over the radio)**  **PATIENT AGE: 27**  **CHIEF COMPLAINT: motor vehicle collision**  **PHYSICAL SETTING: Learner is working in an Emergency Department that provides online medical control to EMS units when he/she is contacted (by radio) by an EMS unit requesting orders.** | |
|  | |
| **Brief narrative description of case** | In this scenario, a prehospital provider is requesting orders from the learner over a radio to allow a patient who has been in an MVC and does meet trauma triage criteria to choose an alternate destination that is not a trauma center. The learner must determine if the patient has decision making capacity and understands the risks of transport to a non-trauma facility, at which point the patient may be allowed to go to his facility of choice. |
| **Primary Learning Objectives** | By the end of this scenario, learners will be able to:   - Demonstrate proper use of the radio - Discuss how to determine whether a patient has decision making capacity - Discuss specialty destination guidelines and how they relate to a patient’s ability to choose their preferred hospital |
| **Critical Actions** | 1. Utilize the radio correctly. 2. Recommends transport to the trauma facility 3. Ensures the patient has decision-making capacity 4. Ensures the patient understands the risks of choosing a facility contrary to destination guidelines 5. Grants permission to transport to an alternate facility |
| **Learner Preparation or Prework** | The learner is an attending physician on duty in the ED of an academic, Level 1 trauma center.  Hospital B is a large community hospital with most specialty services, but it is not designated as a trauma center.  Learners should have reviewed the CDC Field Trauma Triage Criteria before the session |

| Radio report | | | |
| --- | --- | --- | --- |
| **Overall Setting and Appearance** | No specific setting is required other than ensuring that the learner and facilitator are in separate rooms and able to communicate via a two-way radio | | |
| **Confederates (e.g., standardized participants) and their roles in the room at case start** | No additional personnel are required. | | |
| **Facilitator script for opening radio statement** | “Medic 8 to [OLMC]” *(feel free to insert an appropriate callsign for your local institution/area)*  After the learner acknowledges transmission:  “This is Medic 8. I’m on scene with a 27 year-old-male who was the restrained driver involved in a rollover MVC. He swerved to miss a deer in the road, hit the guardrail, and his car rolled over 2 times. He self-extricated and was ambulatory on arrival, but there is about a foot and a half of intrusion into the patient compartment. He is requesting to go to Hospital B. I am calling for trauma clearance to take him to a non-trauma center.”  All information below must be specifically requested by the learner | | |
| **Initial vital signs** | HR: 122, BP: 148/88, Resp: 16, Sats: 98% on RA | | |
| **Past Medical/Surgical History** | **Medications** | **Allergies** | **Family History** |
| None | None | NKDA | N/A |
| **Physical Examination** | | | |
| **General** | Awake, alert, and in no acute distress. Oriented to person, place, time, and situation, and he does recall the accident | | |
| **HEENT** | Normocephalic, atraumatic | | |
| **Neck** | Supple, full range of motion, no tenderness | | |
| **Lungs** | Non-labored breathing, clear to auscultation bilaterally, no signs of trauma to chest | | |
| **Cardiovascular** | Normal S1/S2, tachycardic, regular rhythm, no murmurs | | |
| **Abdomen** | Soft, non-distended, non-tender, no signs of trauma | | |
| **Neurological** | Alert, oriented x4, recalls the incident, cranial nerves intact, normal sensation, moving all extremities. | | |
| **Extremities** | Minor contusions and abrasions to all extremities. No deformity. Moving all extremities normally | | |
| **Skin** | Warm, dry, no obvious rash | | |
| **GU** | *(Not examined)* | | |
| **Psychiatric** | Answering questions appropriately with normal insight and judgement | | |

| Instructor Notes - Changes and CASE Branch Points | |
| --- | --- |
| **Intervention / Time point** | **Change in Case** |
| *If the learner asks for a summary of traumatic injuries* | The provider may simply describe the contusions and abrasions and then state that the rest of the exam is unremarkable. |
| *If the learner asks for transport times to the hospitals* | Trauma center: 15 minutes  Hospital B: 10 minutes |
| *If the learner asks for repeat vital signs* | HR: 118, BP 136/86, Resp: 18, Sats: 99% on RA |
| *After initial report* | Critical Actions:   - Requests a second set of vital signs for stability - Recommends transport to the trauma facility - Ensures the patient has decision-making capacity - Ensures the patient understands the risks of choosing a facility contrary to destination guidelines   The learner should ensure that the patient is fully oriented and understands the risks of going to a non-trauma facility. He/she may do so either by instructing the prehospital provider to inform the patient of the risks or by speaking to the patient directly. The learner should then ensure the patient understands the risks of his decision, including sudden deterioration and death, especially given his tachycardia. |
| *If the learner asks to speak to the patient* | The facilitator should assume the role of the patient and continue to speak to the learner over the radio. The patient is appreciative and understands the learner’s concern, but does not wish to go to the trauma center. |
| *After determining that the patient has decision-making capacity* | **Critical Action:**   - Grants permission to transport to an alternate facility |
| *If the learner decides to call the alternate facility and speak to the receiving doctor.* | The facilitator should assume the role of the receiving doctor. The learner should explain the concern for trauma, but that the patient is proceeding to the alternate facility by his own choice despite understanding the risks. The receiving MD will be appreciative of the information, and assures the learner that they will transfer the patient if any major trauma is noted. |

**Ideal Scenario Flow**

After receiving the initial report, the learner should recognize that this patient does meet criteria to go to a trauma center based on Step Three of the CDC Field Trauma Triage Guidelines. He/she should then take steps to ensure that the patient has decision-making capacity in order to be allowed to appropriately choose an alternate facility. At some point in this process, asking for full physical exam findings will help the learner highlight the potential need for a trauma center to the patient. He/she should also encourage the patient to come to the trauma center, highlight the risks of choosing another facility, and ensure the patient understands those risks. Once these steps have been taken, it is appropriate to allow the patient to choose his destination, and he should be allowed to do so. The learner should also ideally call the alternate facility and inform the receiving doctor that the patient was advised to come to the trauma center, but preferred to be transported to the alternate facility, however this step is not required.

**Anticipated Management Mistakes**

1. If he/she grants the request without any further information. The facilitator may give a one-time hint that further information should be gathered. For example: “Okay. He seemed a little confused at first, so we wanted to be sure it was okay.” If the learner still grants the request without determining decision-making capacity, then the scenario will end. At the beginning of the debrief, the facilitator may state that the patient was later transferred to the trauma center with a spleen laceration. His family is present and angry, asking why he was allowed to go to the other facility in the first place.
2. Failure to obtain a second set of vital signs. The facilitator may provide a one-time hint if the learner does not reassess vital signs. For example: “I was worried he might become more unstable.”
3. Failure to ensure the patient understands the risks of going to an alternate facility. The scenario will end. At the beginning of the debrief, the facilitator may state that the patient was later transferred to the trauma center with a spleen laceration. He is angry and stating that he would have come to the trauma center if he knew the other facility was just going to transfer him.

**Debrief materials**

What is the purpose of specialty destination guidelines?

- Ensure more rapid definitive treatment of specialty issues by directly transporting patients to the appropriate specialty center even if it’s not the closest hospital.
- Common destination guidelines: trauma, STEMI, stroke, pediatrics

Do specialty destination guidelines supersede the patient’s ability to choose his/her destination?

- Can vary between jurisdictions, but in most places, no.
- A patient with decision-making capacity is allowed to choose what care they receive, and as an extension of that, which facility they wish to use.
- The patient should be duly informed of the destination guidelines and why they are recommended, but he/she may still choose if able.

When does a patient have decision-making capacity?

- Must be fully oriented to person, place, time, and situation **PLUS** able to understand the risks of going against recommendations
- Without understanding the risks, they are not making a fully informed decision

**Discussion**

In some areas, EMS must contact medical control when a patient wishes to go to a facility which is contrary to destination guidelines. These situations should be managed similarly to a patient refusal in that the patient must not only be oriented, but also understand the risks inherent in their decision^1^.

A common example of this situation arises within the trauma system when patients who appear well or with minor injuries following a major traumatic event do not wish to go to a trauma center for reasons such a personal preference or perceived longer wait times. The base station physician must again be familiar with the CDC Trauma Field Triage Guide^2^ or the trauma destination guidelines of his/her local area. Upon determining that the patient does meet trauma triage guidelines, the physician should ensure that the patient is aware of this fact and potential downstream complications before being allowed to go to an alternate facility. In this example, the patient was relatively well appearing following a major motor vehicle accident. Given the lack of physical findings beyond bruising, abrasions, and tachycardia, his case could likely be handled by any Emergency Department. However, he must be made aware of the severity of his accident, and the potential need for transfer to a trauma center if any serious injuries are found, as well as the potential medical consequences of the delay of care and financial consequences of the transfer.

References:

1. Weaver J, Brinsfield KH, Dalphond D. Prehospital refusal-of-transport policies: adequate legal protection? Prehosp Emerg Care. 2000;4(1):53-6.
2. Sasser SM, Hunt RC, Faul M, et al. Guidelines for Field Triage of Injured Patients: Recommendations of the National Expert Panel on Field Triage, 2011*. MMWR* 2011;61:1-20.

2011 CDC Field Trauma Triage Guidelines

Step One: assess vital signs and level of consciousness

- Glascow Coma Score ≤ 13
- Systolic blood pressure < 90 mmHg
- Respiratory rate < 10 or > 29 (< 20 in infant aged < 1 year), or need for ventilatory support

Step Two: assess anotomy of injury

- All penetrating injuries to head, neck, torso, and extremities proximal to elbow or knee
- Chest wall instability or deformity (e.g. flail chest)
- Two or more proximal long bone fractures
- Crushed, degloved, mangled, or pulseless extremity
- Amputation proximal to wrist or ankle
- Pelvic fractures
- Open or depressed skull fractures
- Paralysis

**If any condition from Step One or Step Two is present, the patient should be transported to the highest level trauma center within the system

Step Three: Assess mechanism of injury and evidence of high-energy impact

- Falls
  - Adults: >20 feet (one story is equal to 10 feet)
  - Children: >10 feet or two or three times the height of the child
- High-risk auto crash
  - Intrusion, including roof: > 12 inches occupant site; > 18 inches any site
  - Ejection (partial or complete) from automobile
  - Death in same passenger compartment
  - Vehicle telemetry data consistent with a high risk of injury
- Auto vs. pedestrian/bicyclist thrown, run over, or with significant (> 20 mph) impact
- Motorcycle crash > 20 mph

**If any condition from Step Three is present, the patient should be transported to a trauma center, but it need not be the highest level center in the system

Step Four: Assess special patient or system considerations

- Older adults
  - Risk of injury/death increases after age 55 years
  - SBP < 110 might represent shock after age 65 years
  - Low impact mechanisms (e.g. ground level falls) might result in severe injury
- Children
  - Should be triaged preferentially to pediatric capable trauma centers
- Anticoagulants and bleeding disorders
  - Patients with head injury are at high risk for rapid deterioration
- Burns
  - Without other trauma mechanism: triage to burn facility
  - With trauma mechanism: triage to trauma center
- Pregnancy > 20 weeks
- EMS provider judgement

**If any condition from Step Four is present, the patient should be transported to a trauma center or any hospital capable of timely and through evaluation and initial management of potentially serious injuries. If no condition from any of Steps One through Four are present, transport based on local protocol.
