# Supplementary material for: Online Medical Control for EMS: A Lecture and Case-Based Teaching Module
Source: MedEdPORTAL. 2020 May 15;16:10902. doi: 10.15766/mep_2374-8265.10902 (PMC7331954; doi:10.15766/mep_2374-8265.10902)
Supplement: Supplementary file 1 — OLMC Scenarios.docxIntro to EMS.pptxMedical Oversight of EMS.pptxSurvey.docxTest and Key.docxLecture Outlines.docx [file mep_2374-8265.10902-s001.zip › B. Intro to EMS.pptx]

## Slide 1
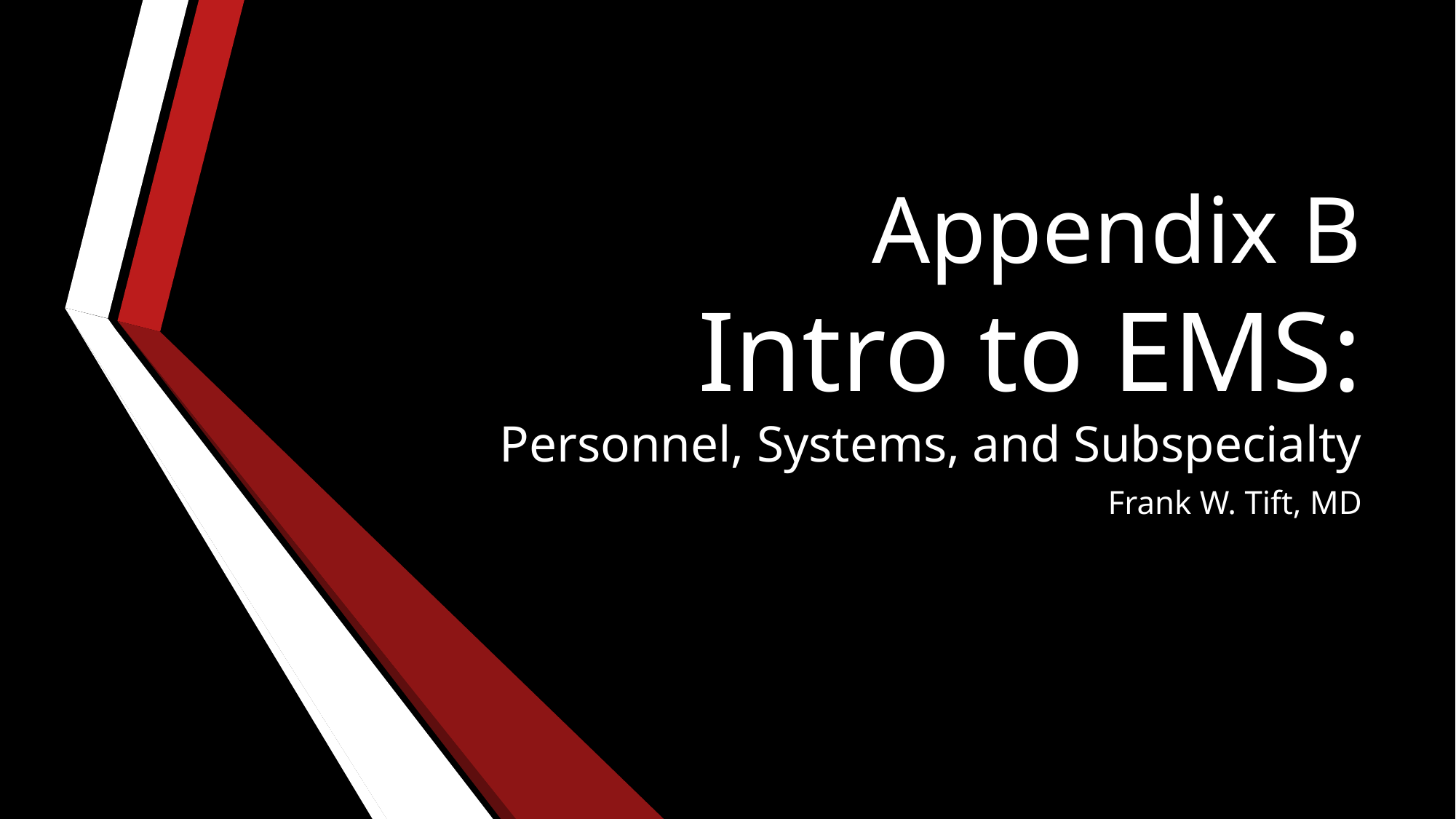

# Appendix B Intro to EMS: Personnel, Systems, and Subspecialty
Frank W. Tift, MD

## Slide 2
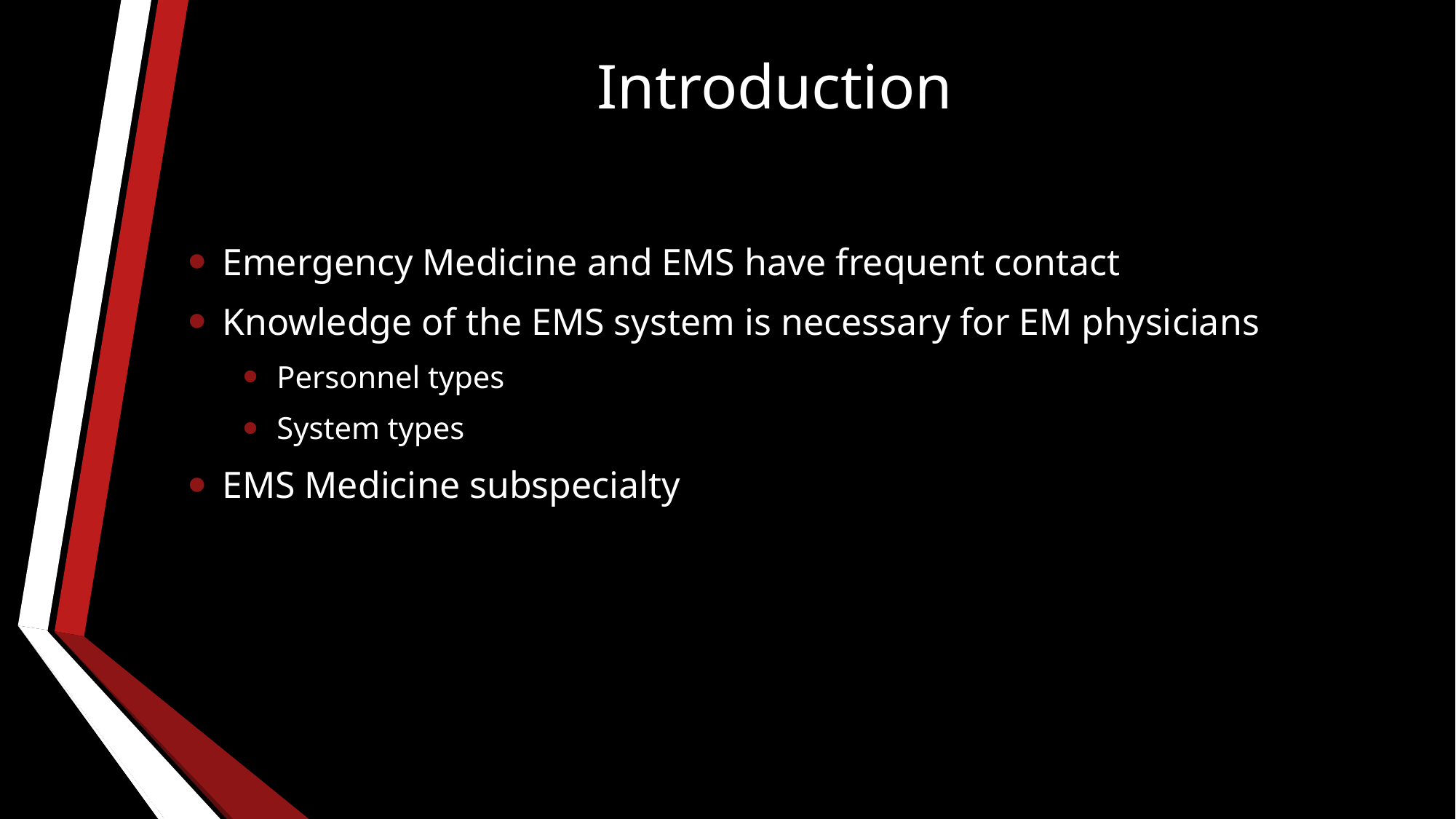

# Introduction
Emergency Medicine and EMS have frequent contact
Knowledge of the EMS system is necessary for EM physicians
Personnel types
System types
EMS Medicine subspecialty

## Slide 3
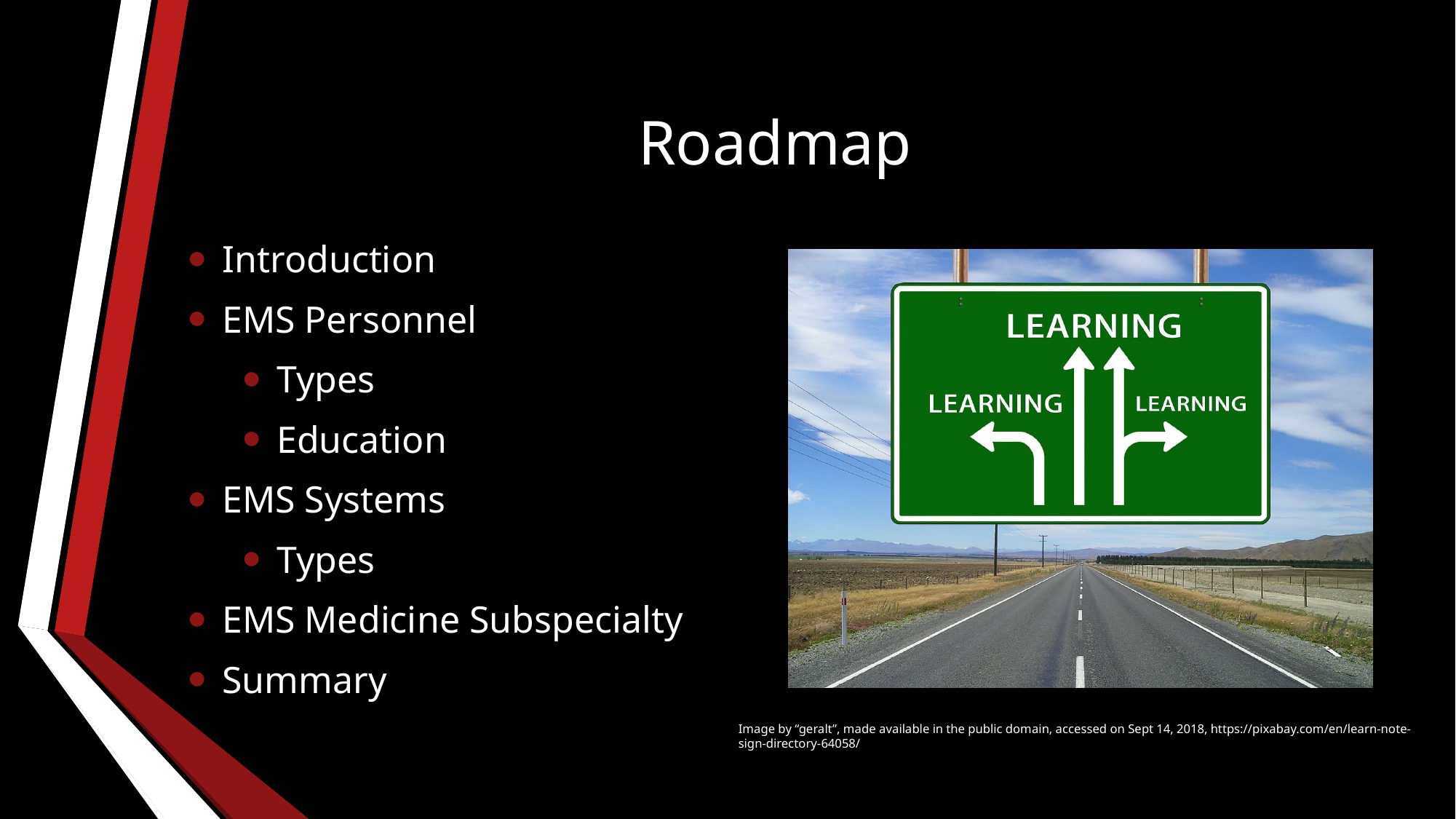

# Roadmap
Introduction
EMS Personnel
Types
Education
EMS Systems
Types
EMS Medicine Subspecialty
Summary
Image by “geralt”, made available in the public domain, accessed on Sept 14, 2018, https://pixabay.com/en/learn-note-sign-directory-64058/

## Slide 4
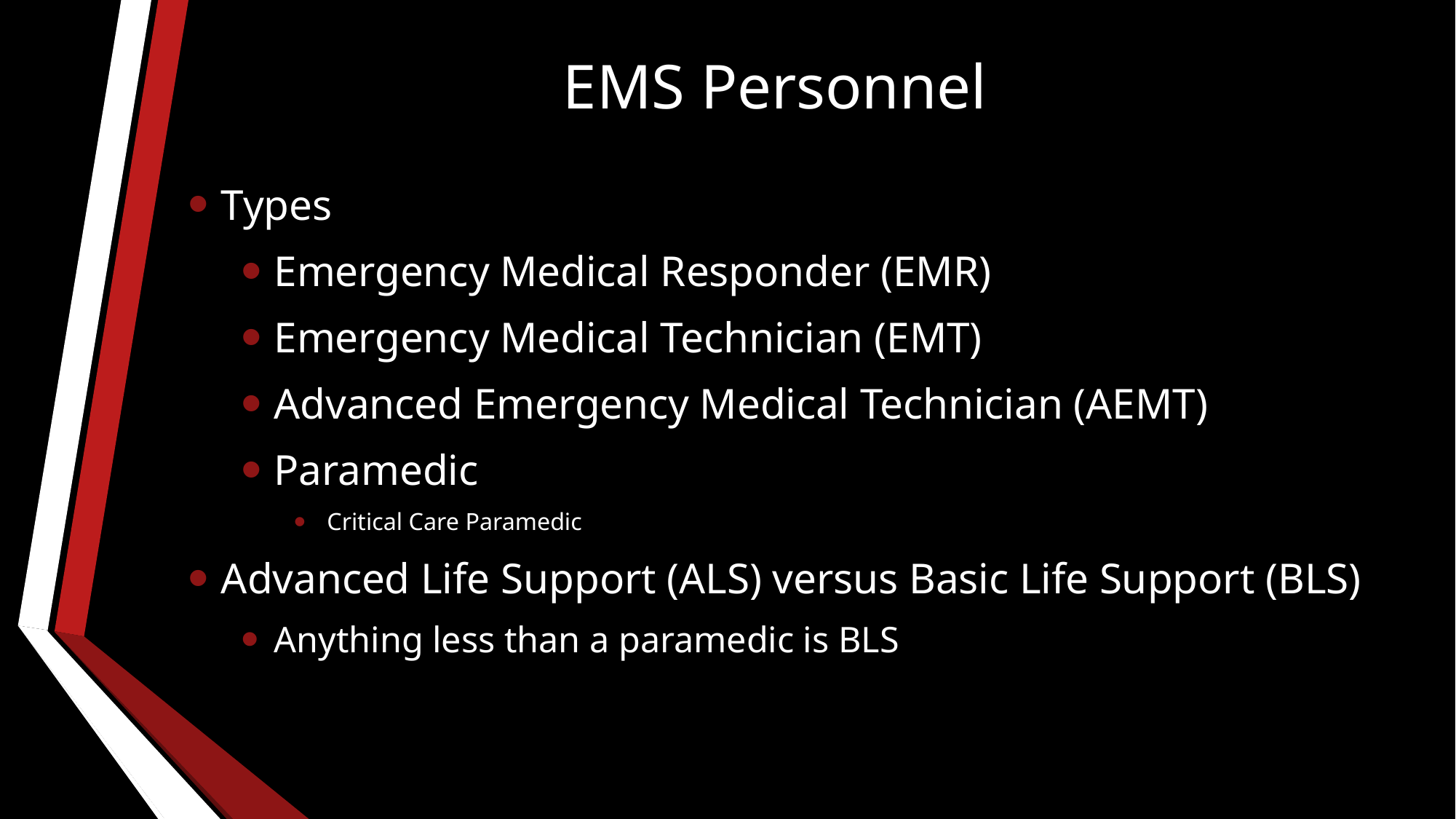

# EMS Personnel
Types
Emergency Medical Responder (EMR)
Emergency Medical Technician (EMT)
Advanced Emergency Medical Technician (AEMT)
Paramedic
Critical Care Paramedic
Advanced Life Support (ALS) versus Basic Life Support (BLS)
Anything less than a paramedic is BLS

## Slide 5
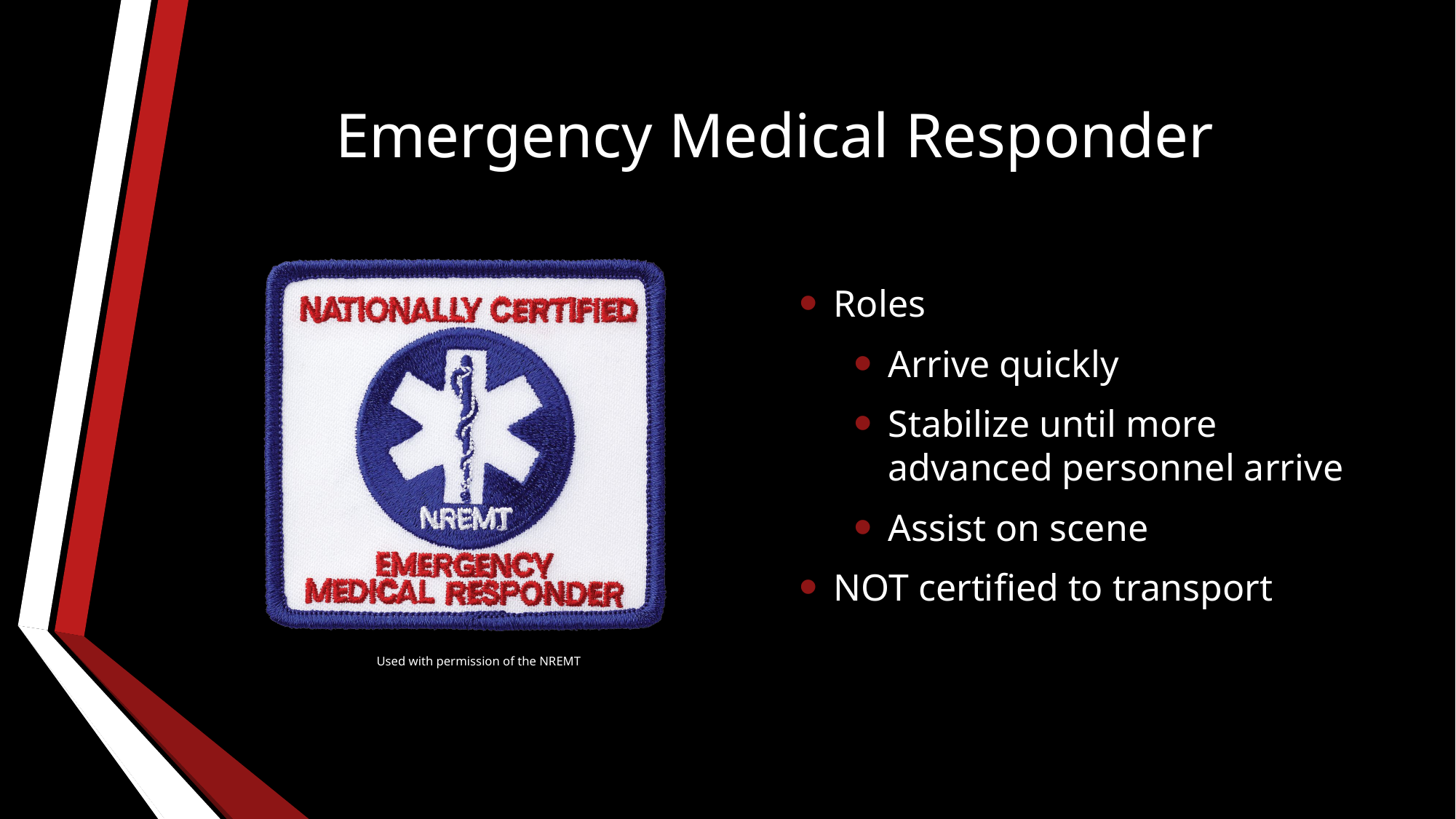

# Emergency Medical Responder
Roles
Arrive quickly
Stabilize until more advanced personnel arrive
Assist on scene
NOT certified to transport
Used with permission of the NREMT

## Slide 6
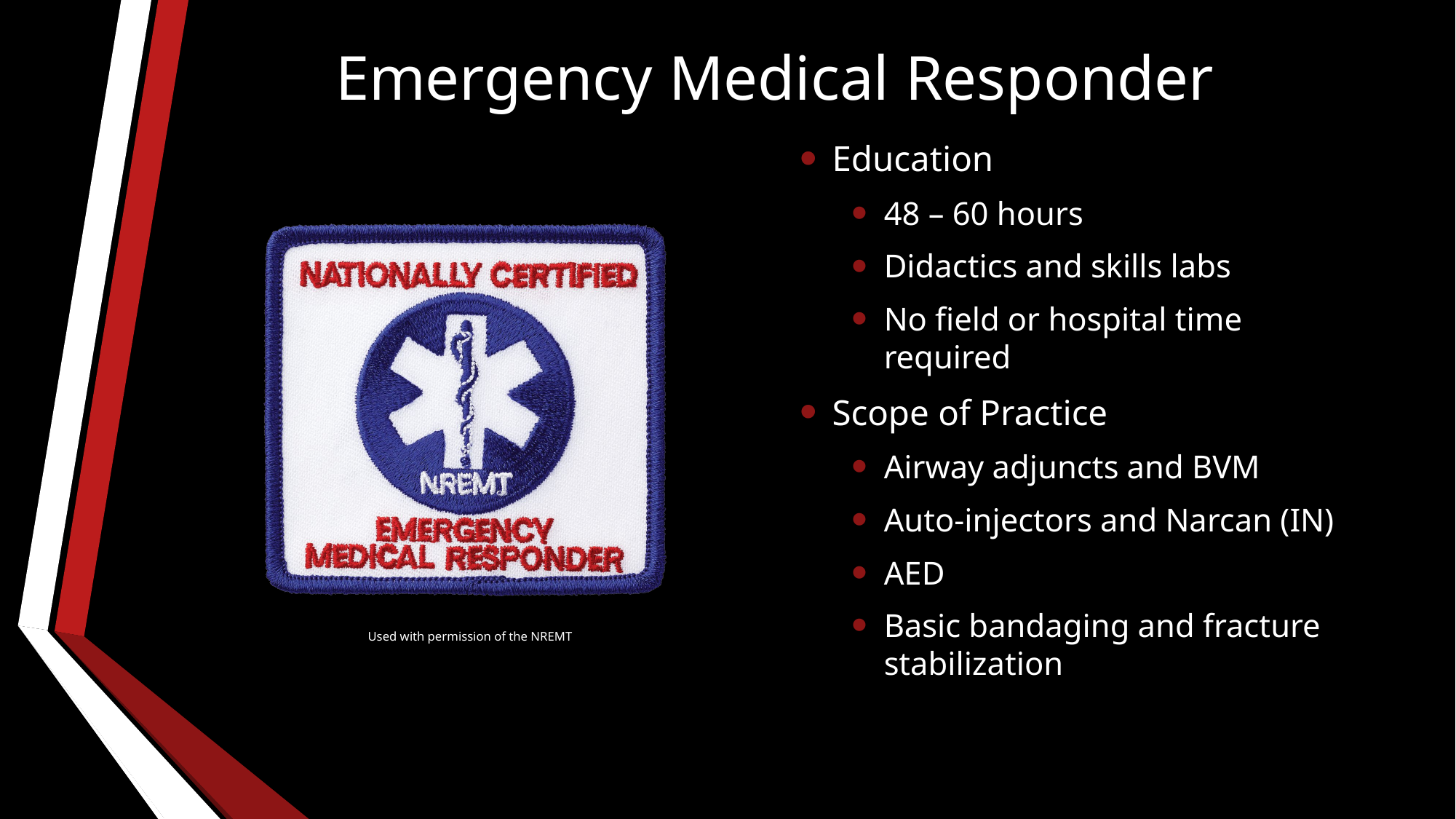

# Emergency Medical Responder
Education
48 – 60 hours
Didactics and skills labs
No field or hospital time required
Scope of Practice
Airway adjuncts and BVM
Auto-injectors and Narcan (IN)
AED
Basic bandaging and fracture stabilization
Used with permission of the NREMT

## Slide 7
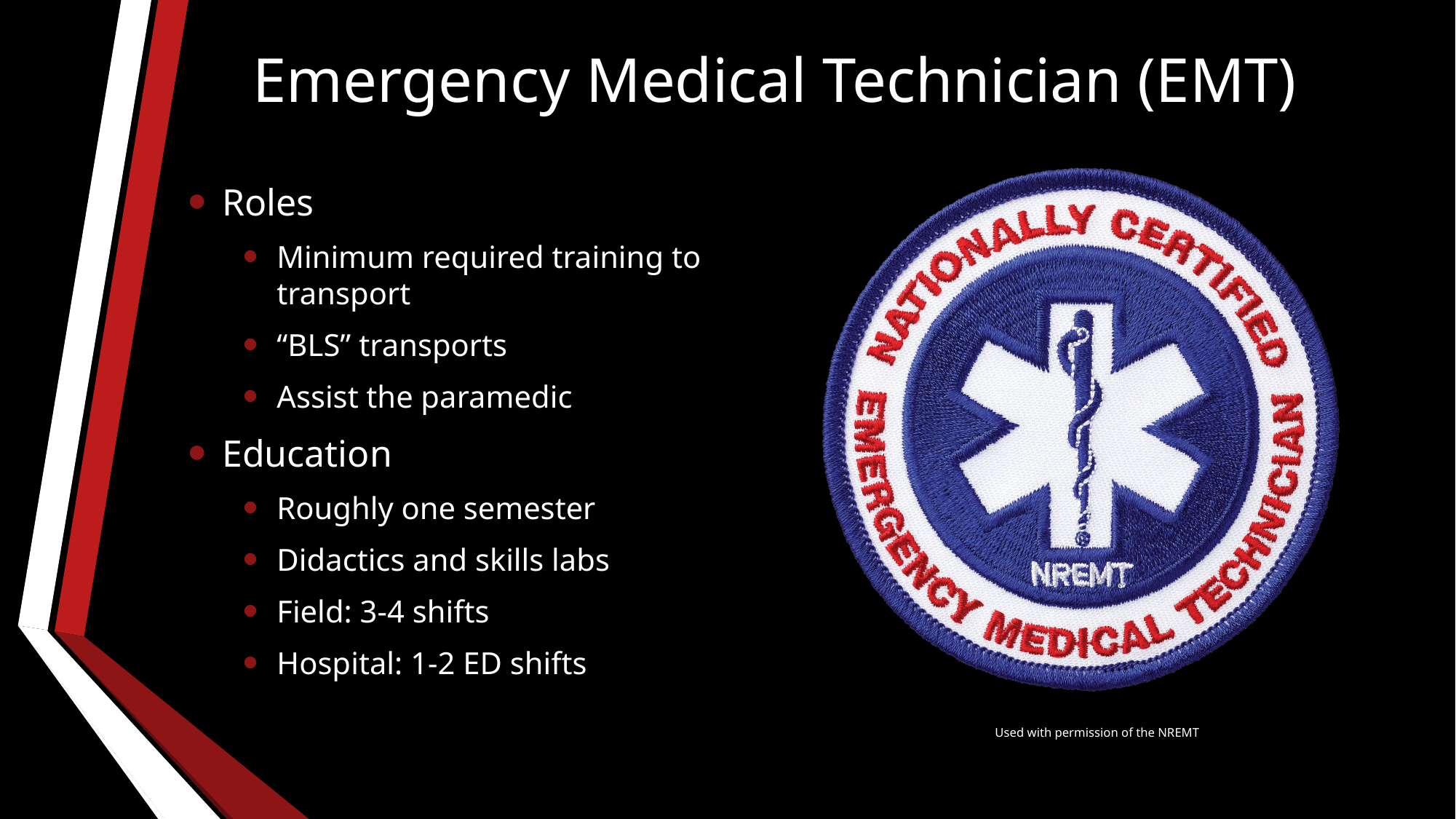

# Emergency Medical Technician (EMT)
Roles
Minimum required training to transport
“BLS” transports
Assist the paramedic
Education
Roughly one semester
Didactics and skills labs
Field: 3-4 shifts
Hospital: 1-2 ED shifts
Used with permission of the NREMT

## Slide 8
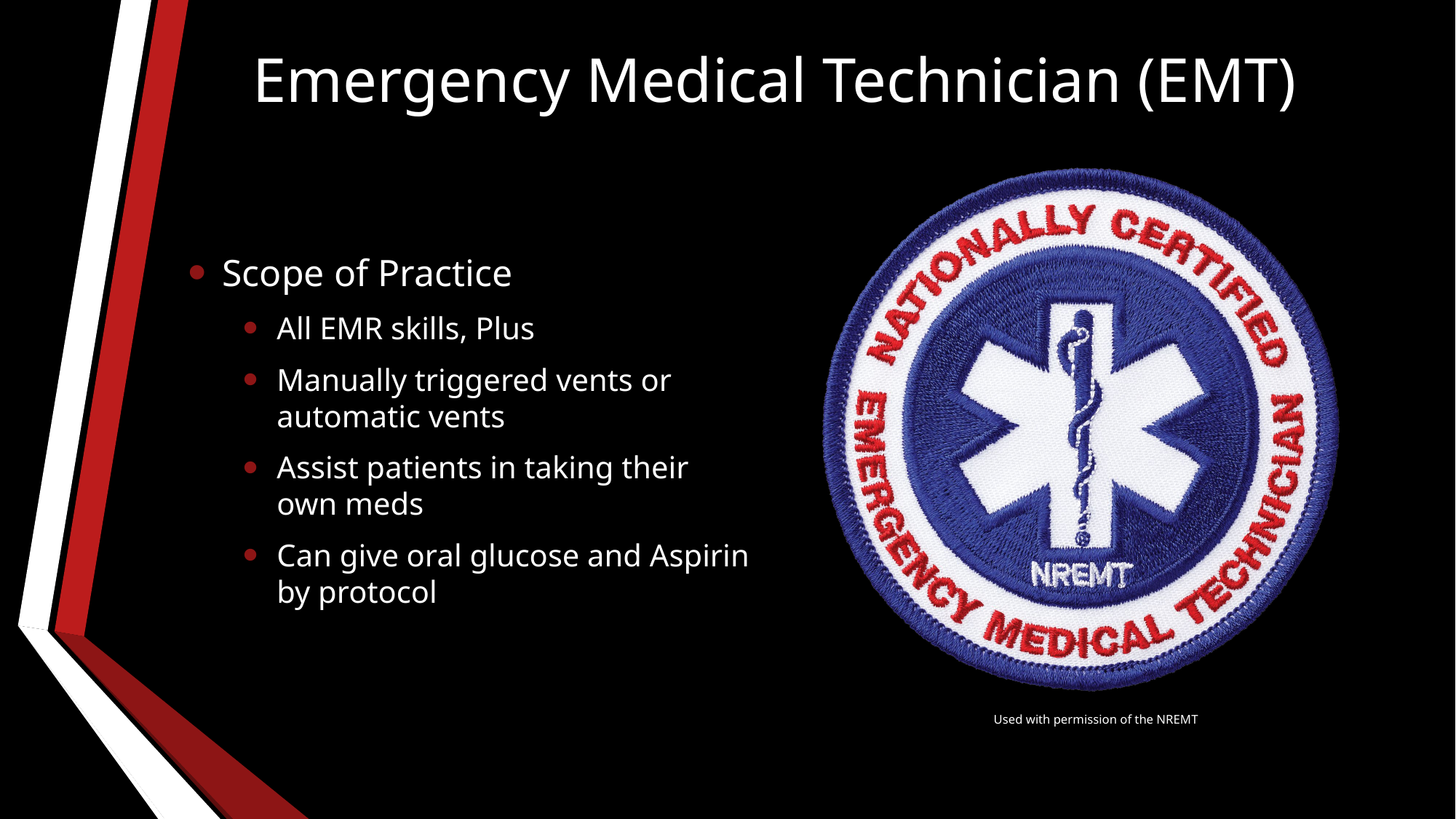

# Emergency Medical Technician (EMT)
Scope of Practice
All EMR skills, Plus
Manually triggered vents or automatic vents
Assist patients in taking their own meds
Can give oral glucose and Aspirin by protocol
Used with permission of the NREMT

## Slide 9
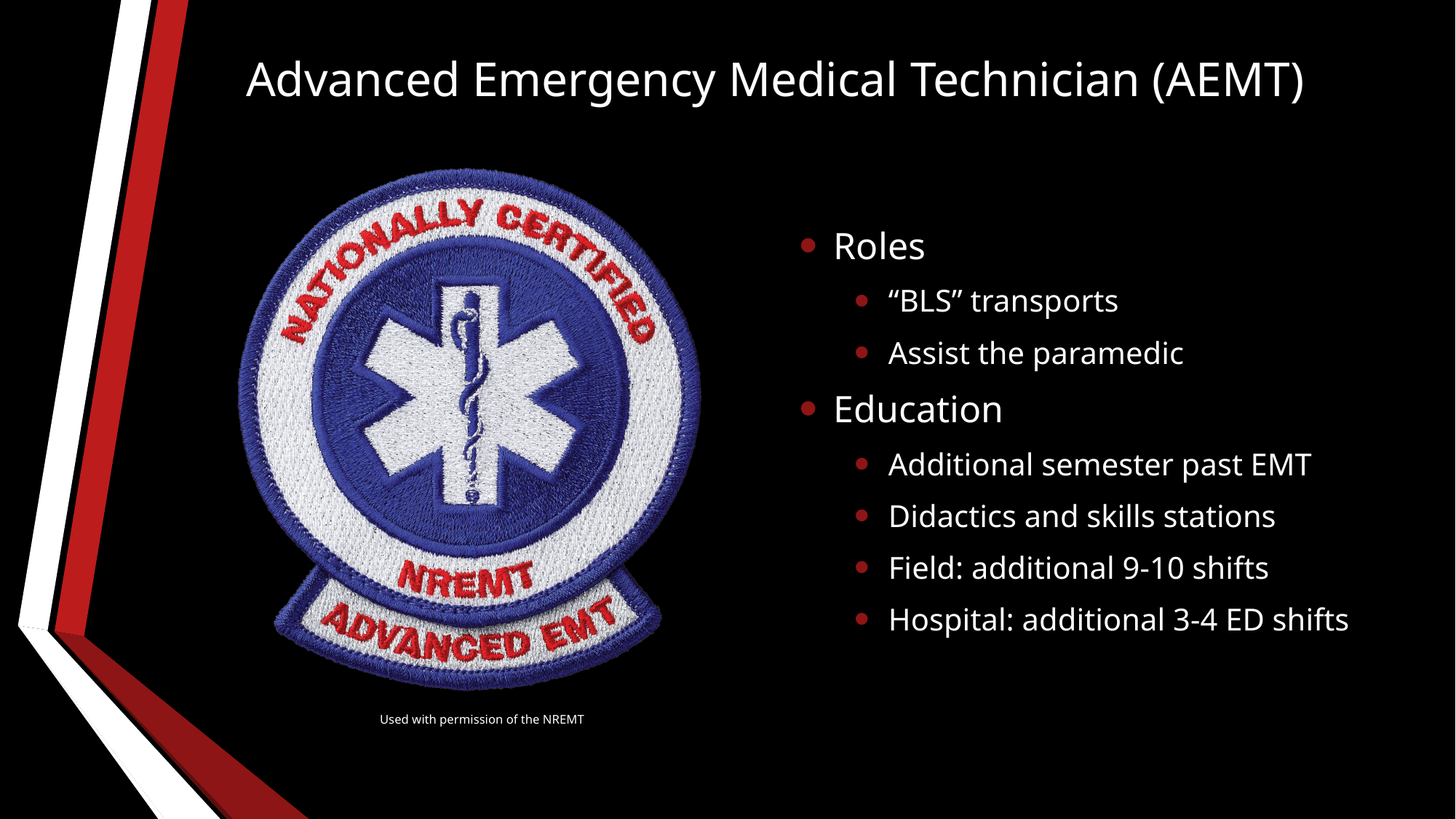

# Advanced Emergency Medical Technician (AEMT)
Roles
“BLS” transports
Assist the paramedic
Education
Additional semester past EMT
Didactics and skills stations
Field: additional 9-10 shifts
Hospital: additional 3-4 ED shifts
Used with permission of the NREMT

## Slide 10
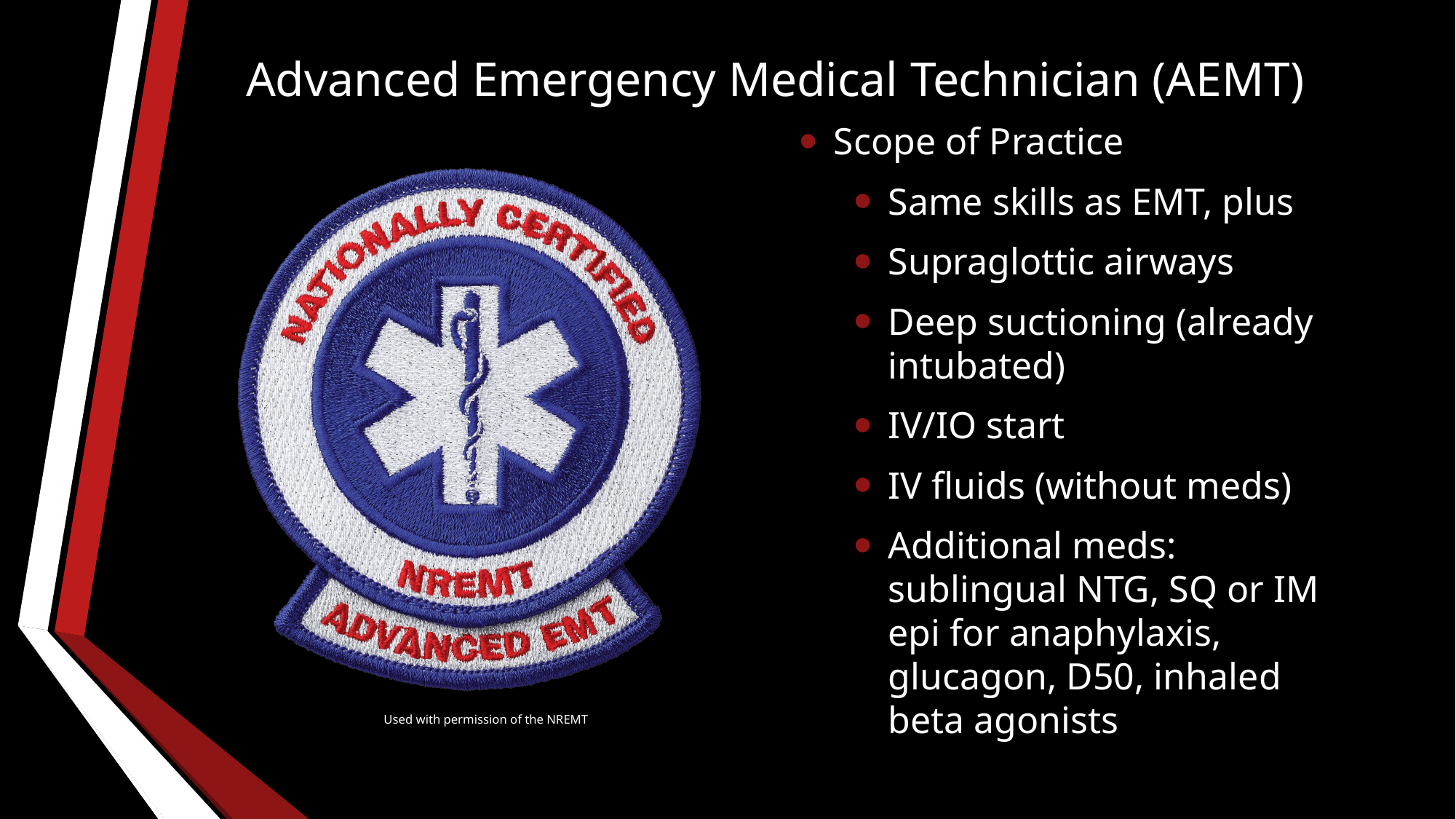

# Advanced Emergency Medical Technician (AEMT)
Scope of Practice
Same skills as EMT, plus
Supraglottic airways
Deep suctioning (already intubated)
IV/IO start
IV fluids (without meds)
Additional meds: sublingual NTG, SQ or IM epi for anaphylaxis, glucagon, D50, inhaled beta agonists
Used with permission of the NREMT

## Slide 11
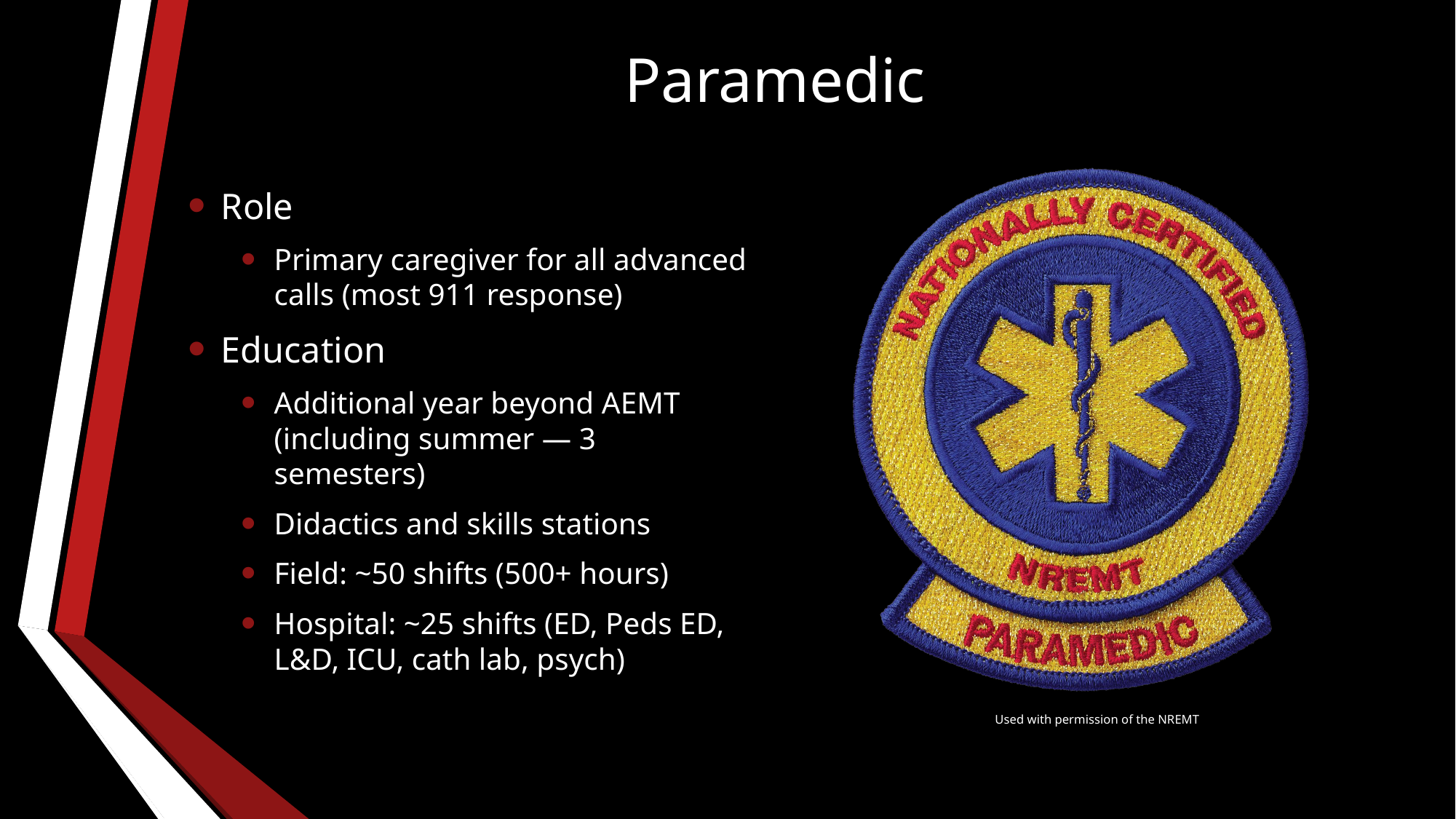

# Paramedic
Role
Primary caregiver for all advanced calls (most 911 response)
Education
Additional year beyond AEMT (including summer — 3 semesters)
Didactics and skills stations
Field: ~50 shifts (500+ hours)
Hospital: ~25 shifts (ED, Peds ED, L&D, ICU, cath lab, psych)
Used with permission of the NREMT

## Slide 12
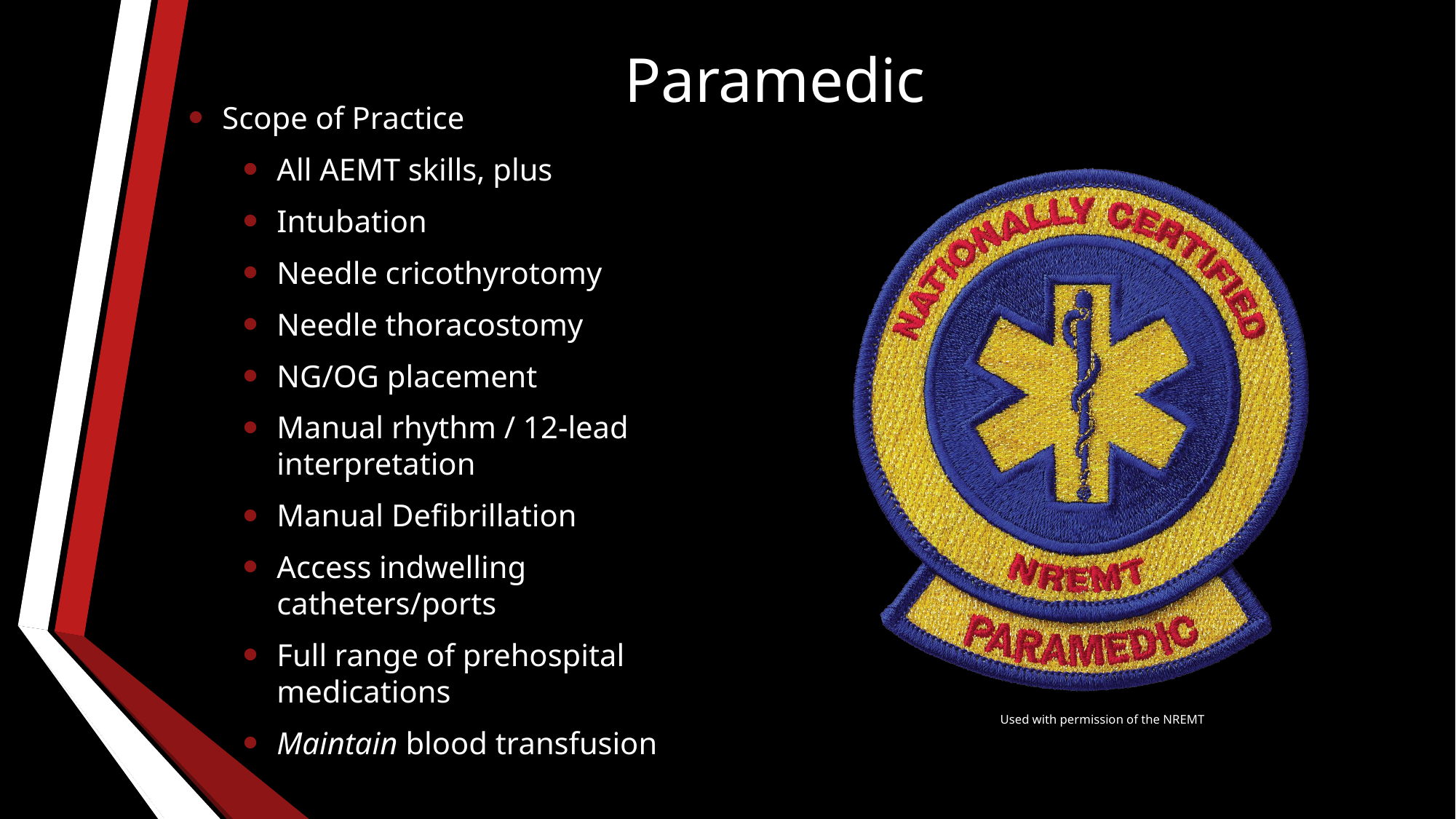

# Paramedic
Scope of Practice
All AEMT skills, plus
Intubation
Needle cricothyrotomy
Needle thoracostomy
NG/OG placement
Manual rhythm / 12-lead interpretation
Manual Defibrillation
Access indwelling catheters/ports
Full range of prehospital medications
Maintain blood transfusion
Used with permission of the NREMT

## Slide 13
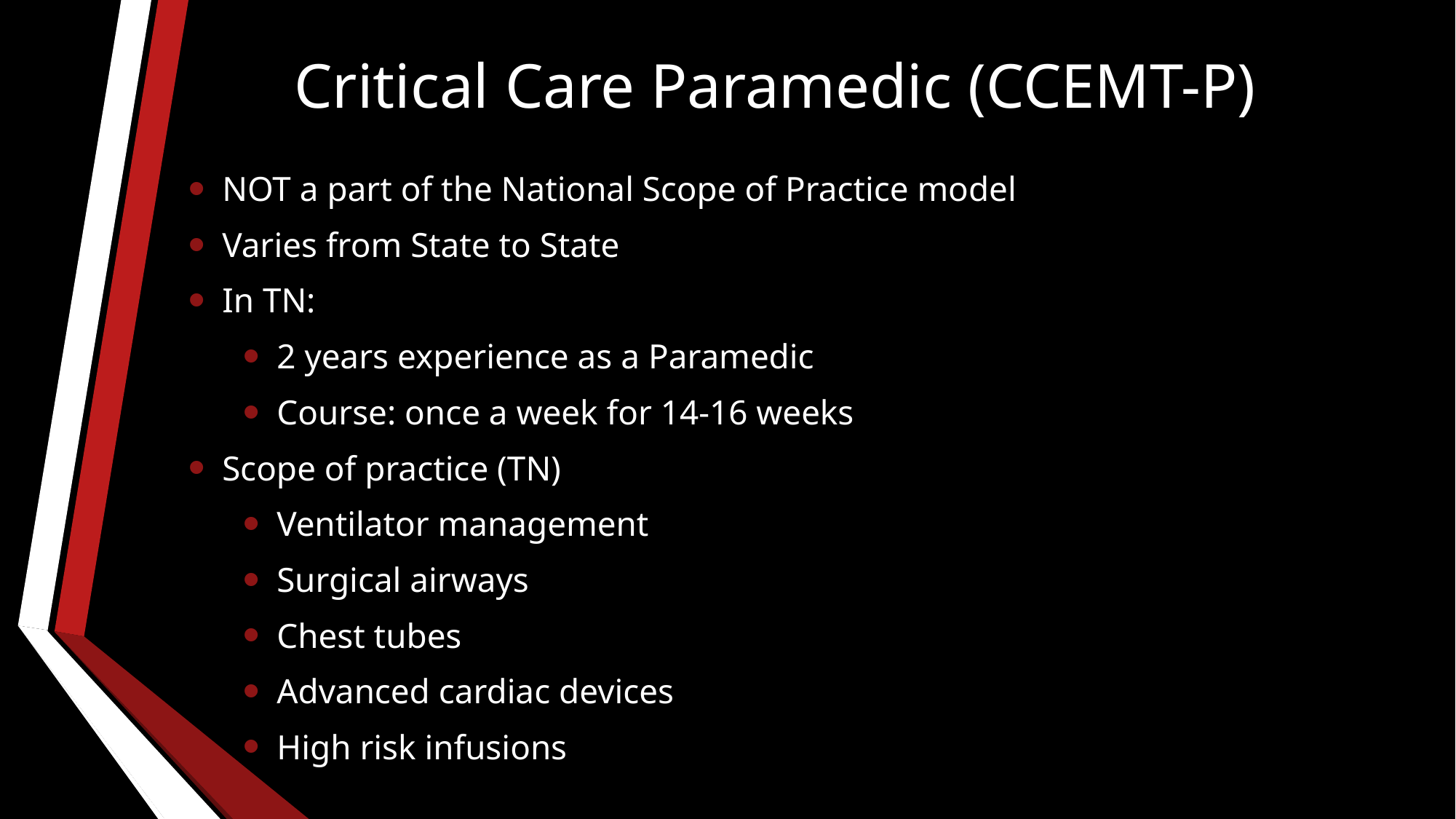

# Critical Care Paramedic (CCEMT-P)
NOT a part of the National Scope of Practice model
Varies from State to State
In TN:
2 years experience as a Paramedic
Course: once a week for 14-16 weeks
Scope of practice (TN)
Ventilator management
Surgical airways
Chest tubes
Advanced cardiac devices
High risk infusions

## Slide 14
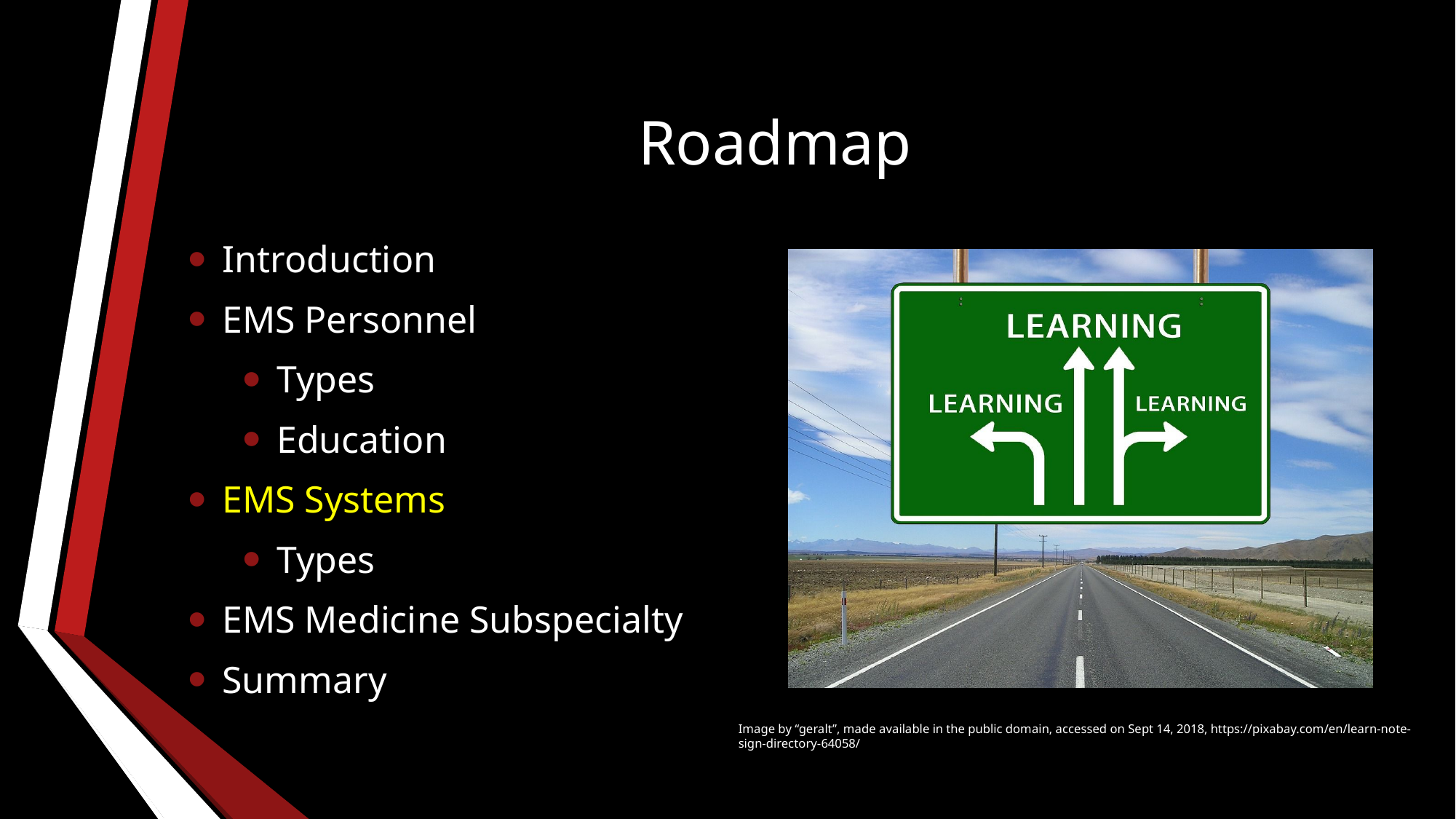

# Roadmap
Introduction
EMS Personnel
Types
Education
EMS Systems
Types
EMS Medicine Subspecialty
Summary
Image by “geralt”, made available in the public domain, accessed on Sept 14, 2018, https://pixabay.com/en/learn-note-sign-directory-64058/

## Slide 15
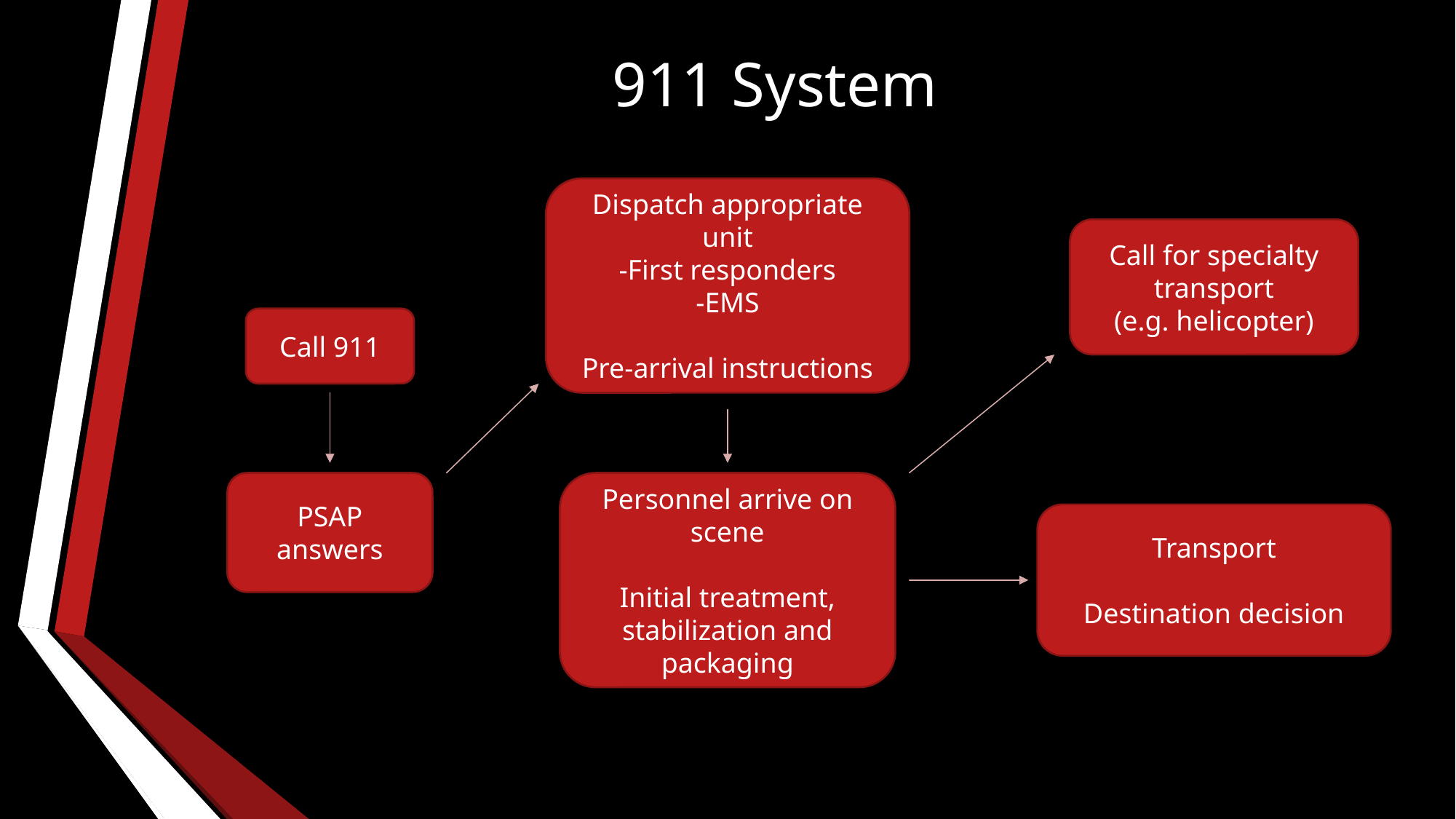

# 911 System
Dispatch appropriate unit
-First responders
-EMS
Pre-arrival instructions
Call for specialty transport
(e.g. helicopter)
Call 911
PSAP answers
Personnel arrive on scene
Initial treatment, stabilization and packaging
Transport
Destination decision

## Slide 16
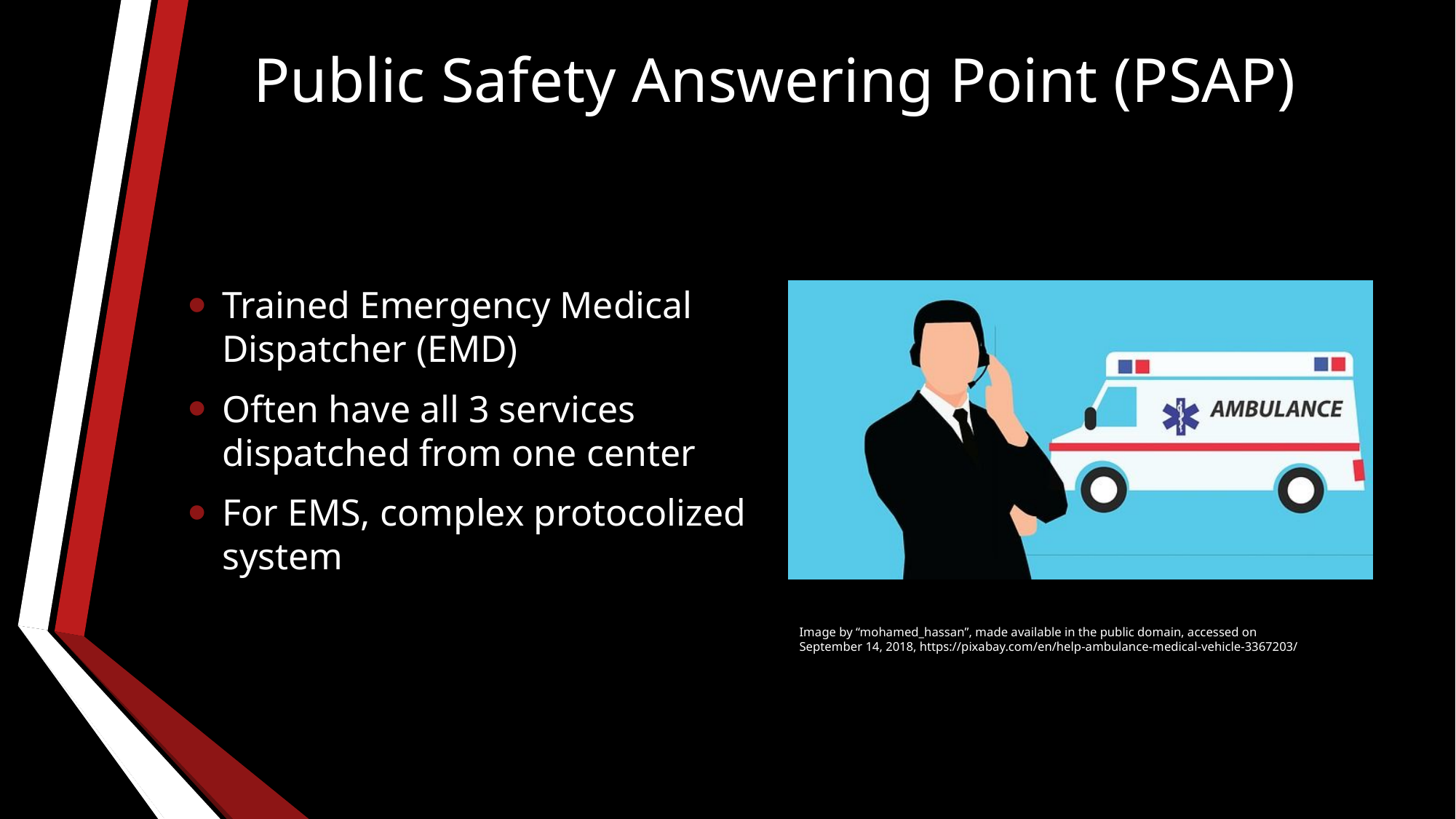

# Public Safety Answering Point (PSAP)
Trained Emergency Medical Dispatcher (EMD)
Often have all 3 services dispatched from one center
For EMS, complex protocolized system
Image by “mohamed_hassan”, made available in the public domain, accessed on September 14, 2018, https://pixabay.com/en/help-ambulance-medical-vehicle-3367203/

## Slide 17
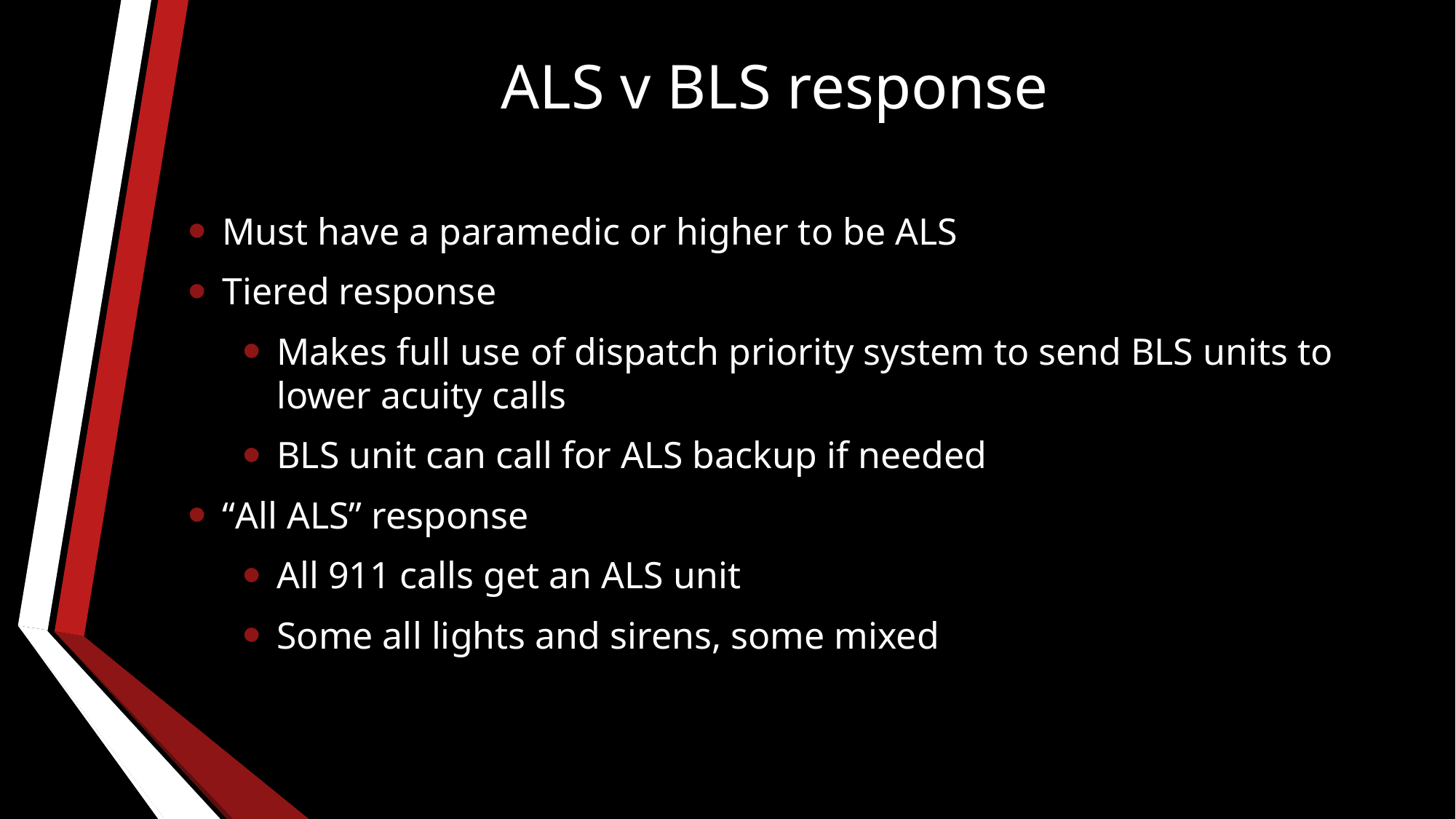

# ALS v BLS response
Must have a paramedic or higher to be ALS
Tiered response
Makes full use of dispatch priority system to send BLS units to lower acuity calls
BLS unit can call for ALS backup if needed
“All ALS” response
All 911 calls get an ALS unit
Some all lights and sirens, some mixed

## Slide 18
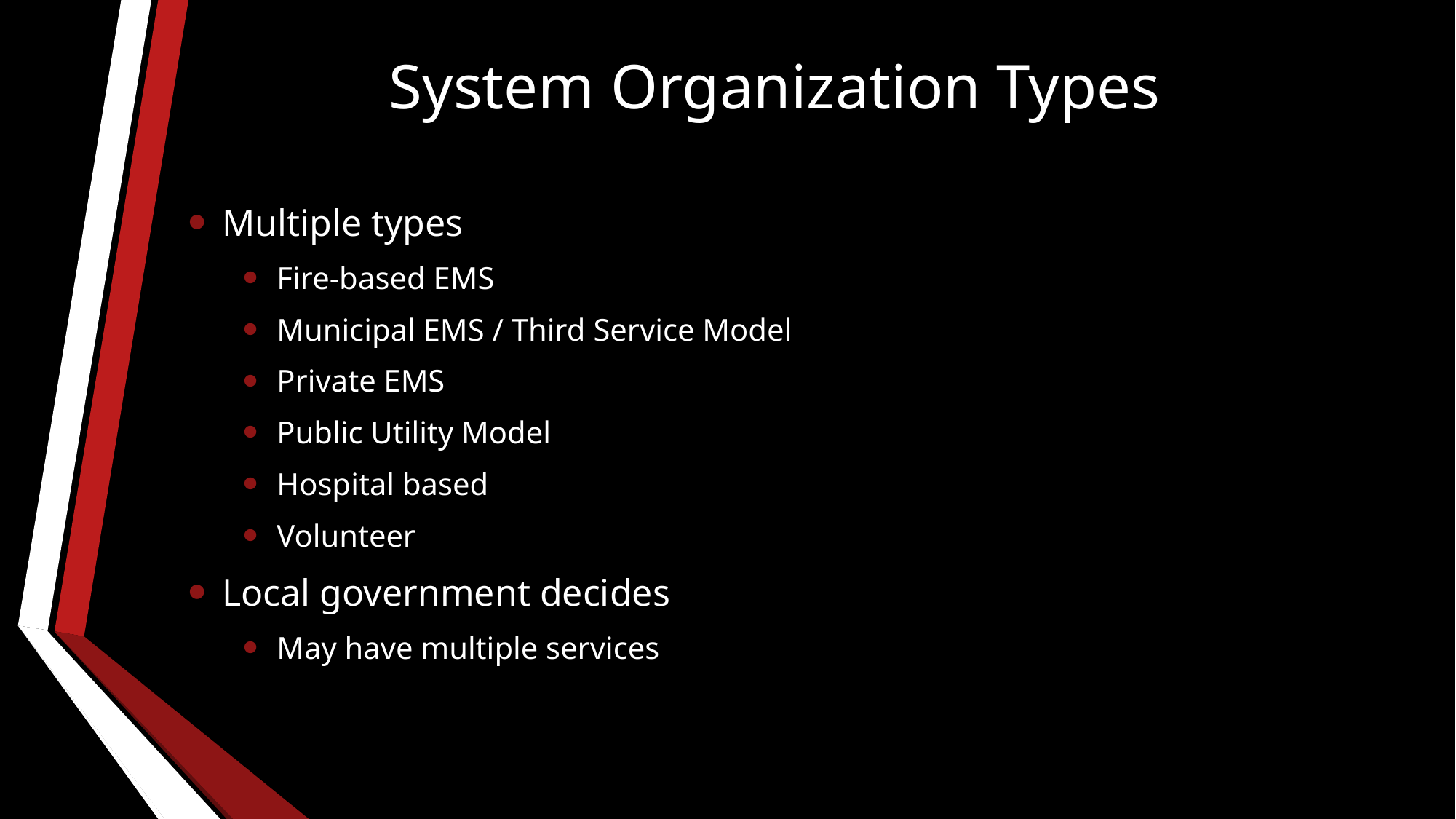

# System Organization Types
Multiple types
Fire-based EMS
Municipal EMS / Third Service Model
Private EMS
Public Utility Model
Hospital based
Volunteer
Local government decides
May have multiple services

## Slide 19
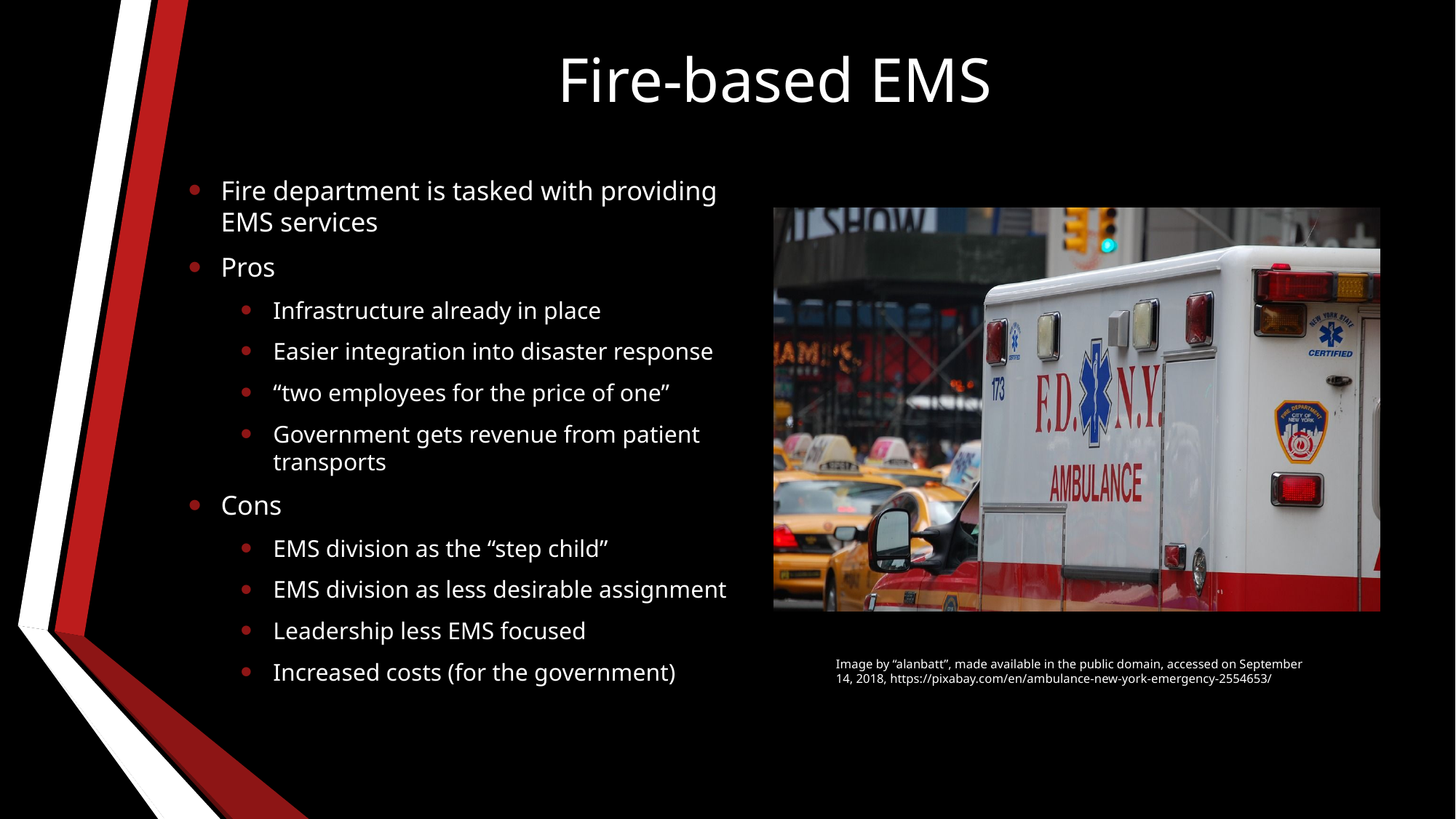

# Fire-based EMS
Fire department is tasked with providing EMS services
Pros
Infrastructure already in place
Easier integration into disaster response
“two employees for the price of one”
Government gets revenue from patient transports
Cons
EMS division as the “step child”
EMS division as less desirable assignment
Leadership less EMS focused
Increased costs (for the government)
Image by “alanbatt”, made available in the public domain, accessed on September 14, 2018, https://pixabay.com/en/ambulance-new-york-emergency-2554653/

## Slide 20
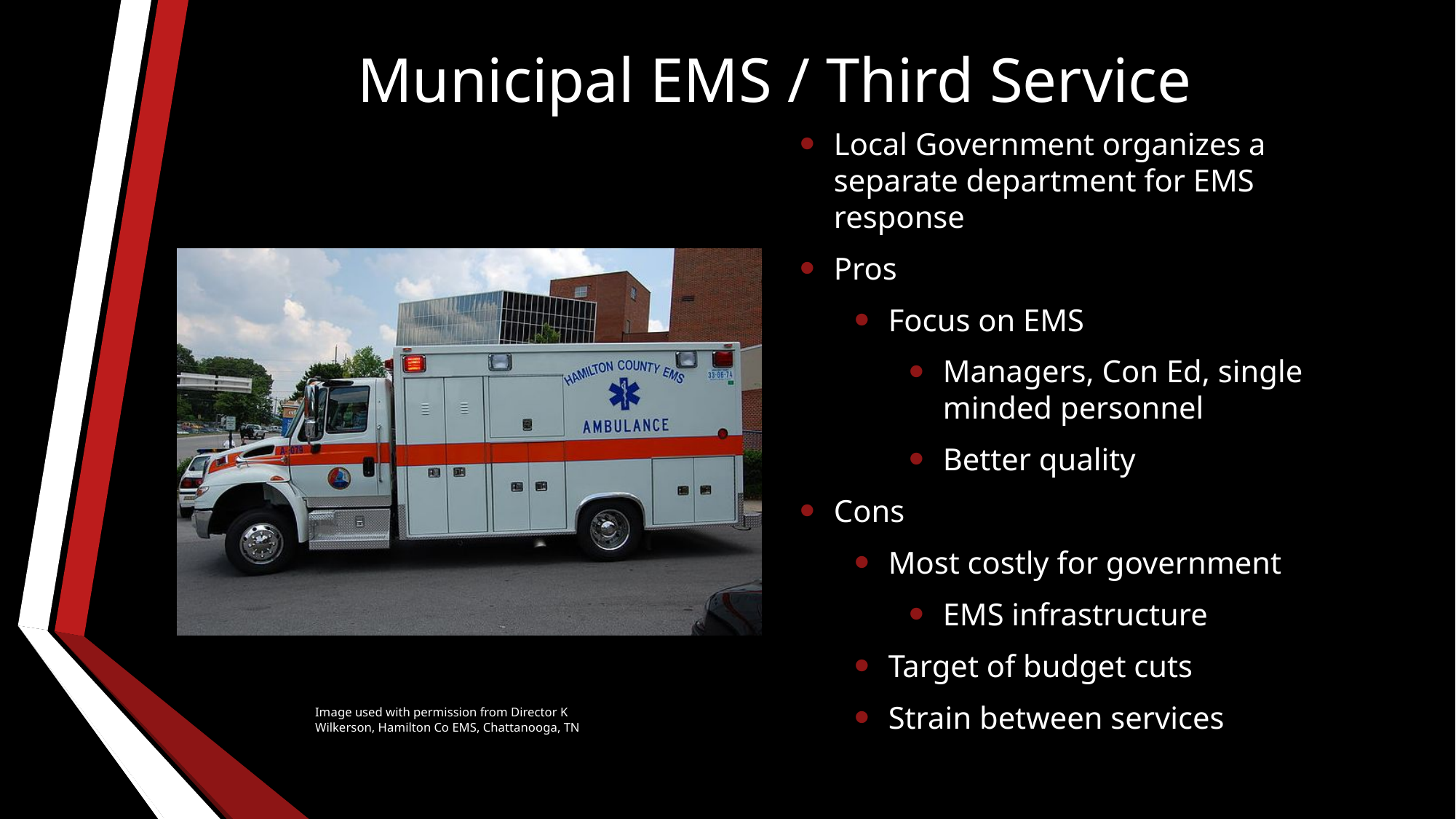

# Municipal EMS / Third Service
Local Government organizes a separate department for EMS response
Pros
Focus on EMS
Managers, Con Ed, single minded personnel
Better quality
Cons
Most costly for government
EMS infrastructure
Target of budget cuts
Strain between services
Image used with permission from Director K Wilkerson, Hamilton Co EMS, Chattanooga, TN

## Slide 21
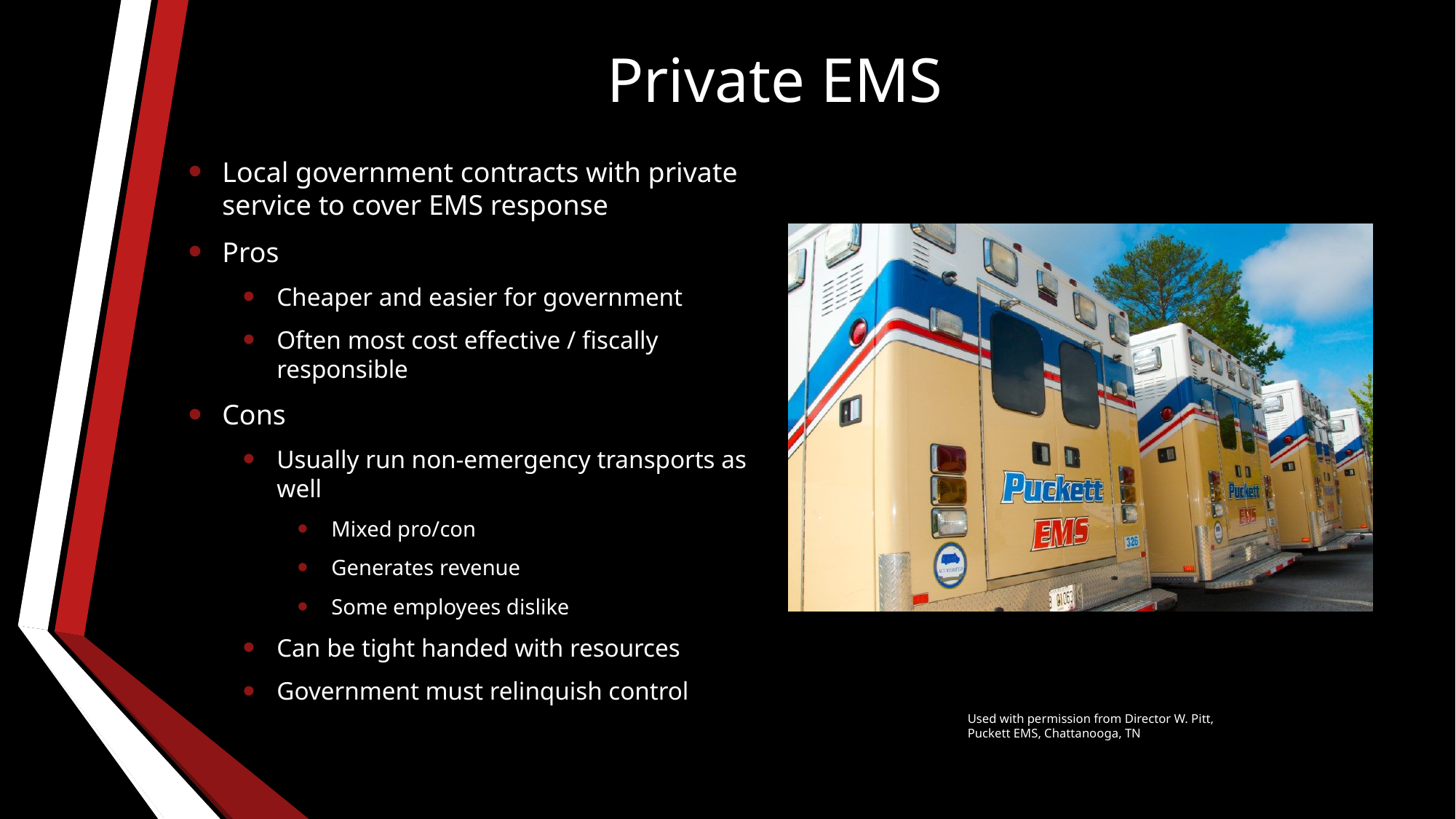

# Private EMS
Local government contracts with private service to cover EMS response
Pros
Cheaper and easier for government
Often most cost effective / fiscally responsible
Cons
Usually run non-emergency transports as well
Mixed pro/con
Generates revenue
Some employees dislike
Can be tight handed with resources
Government must relinquish control
Used with permission from Director W. Pitt,
Puckett EMS, Chattanooga, TN

## Slide 22
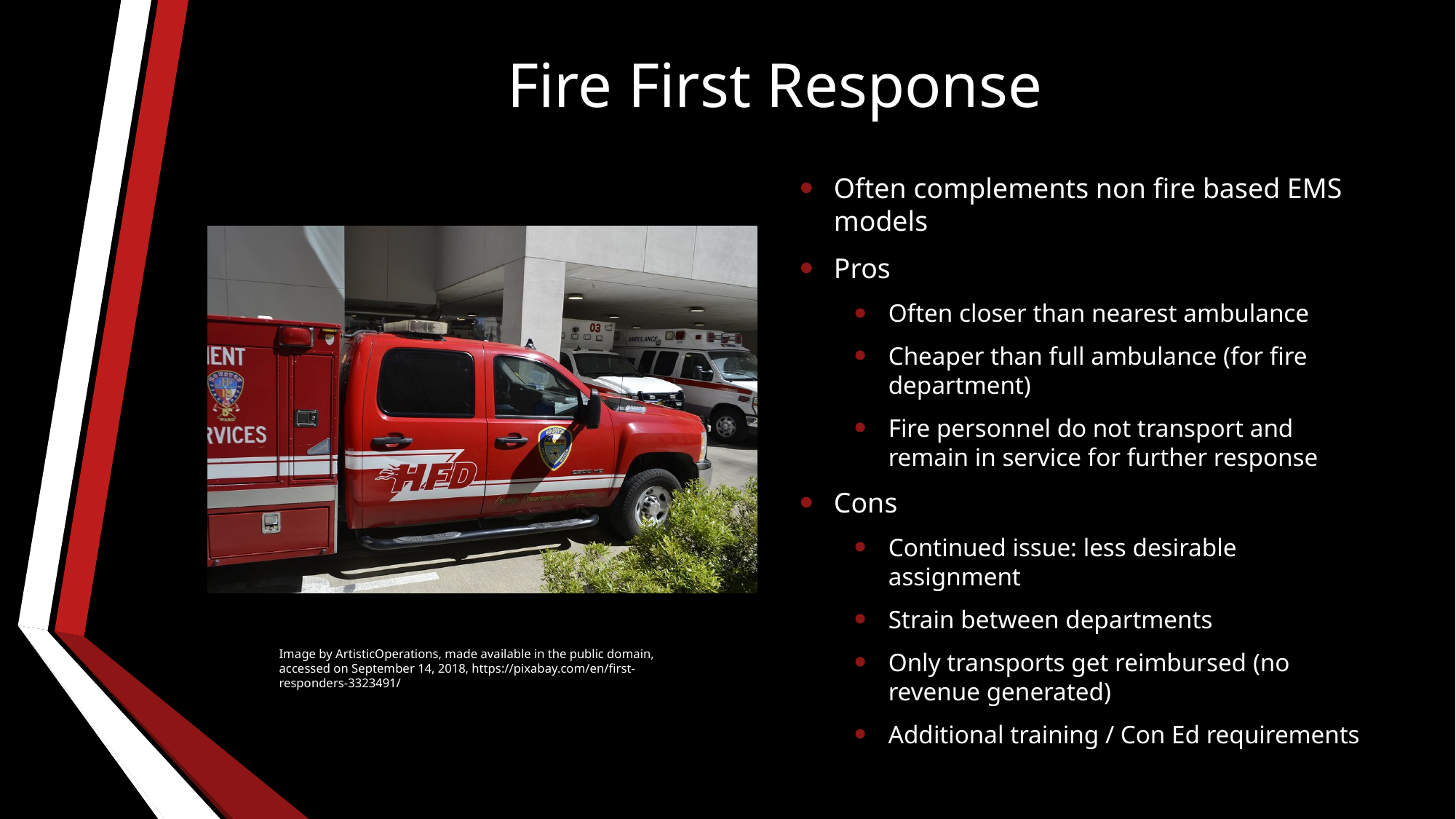

# Fire First Response
Often complements non fire based EMS models
Pros
Often closer than nearest ambulance
Cheaper than full ambulance (for fire department)
Fire personnel do not transport and remain in service for further response
Cons
Continued issue: less desirable assignment
Strain between departments
Only transports get reimbursed (no revenue generated)
Additional training / Con Ed requirements
Image by ArtisticOperations, made available in the public domain, accessed on September 14, 2018, https://pixabay.com/en/first-responders-3323491/

## Slide 23
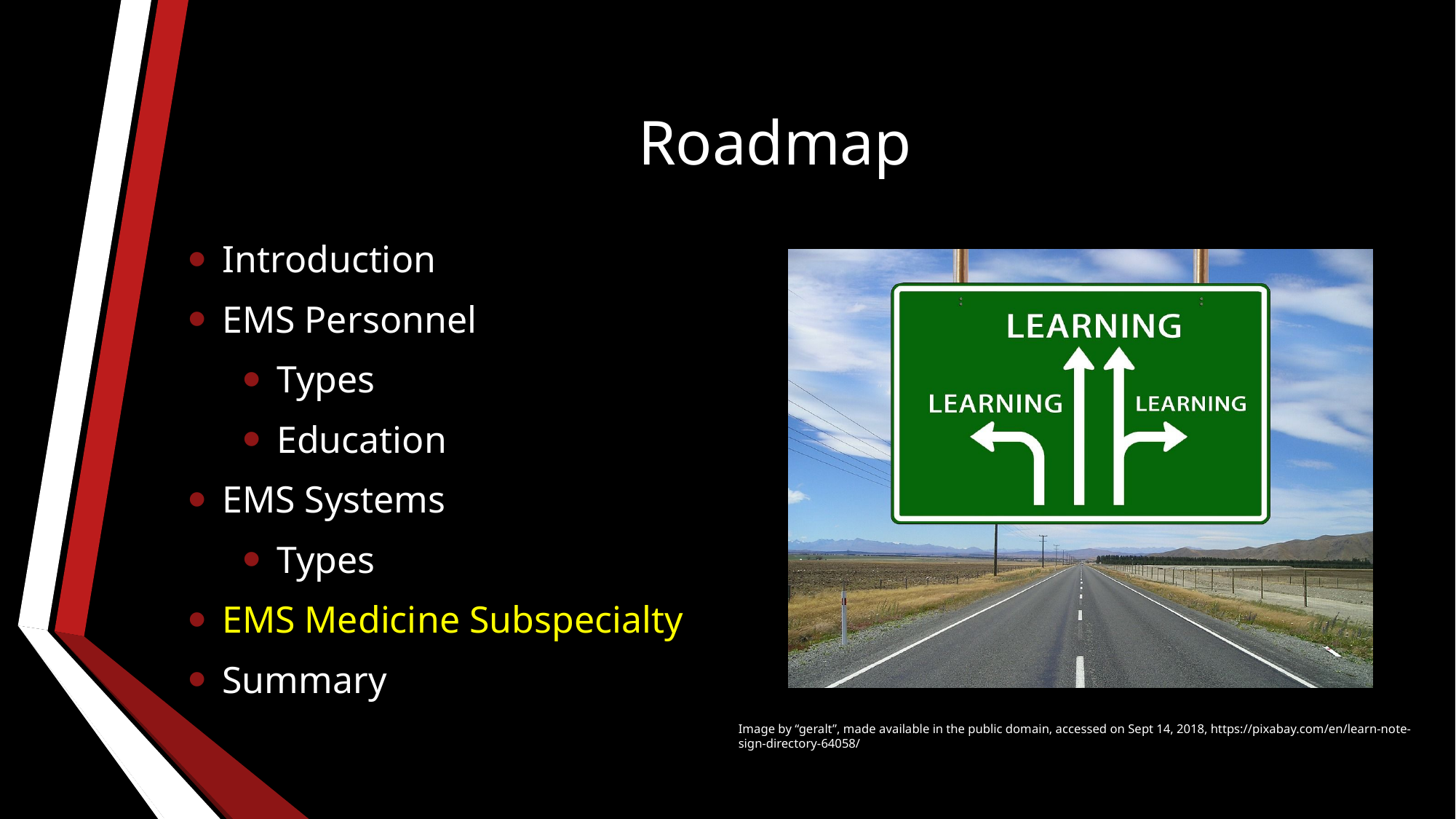

# Roadmap
Introduction
EMS Personnel
Types
Education
EMS Systems
Types
EMS Medicine Subspecialty
Summary
Image by “geralt”, made available in the public domain, accessed on Sept 14, 2018, https://pixabay.com/en/learn-note-sign-directory-64058/

## Slide 24
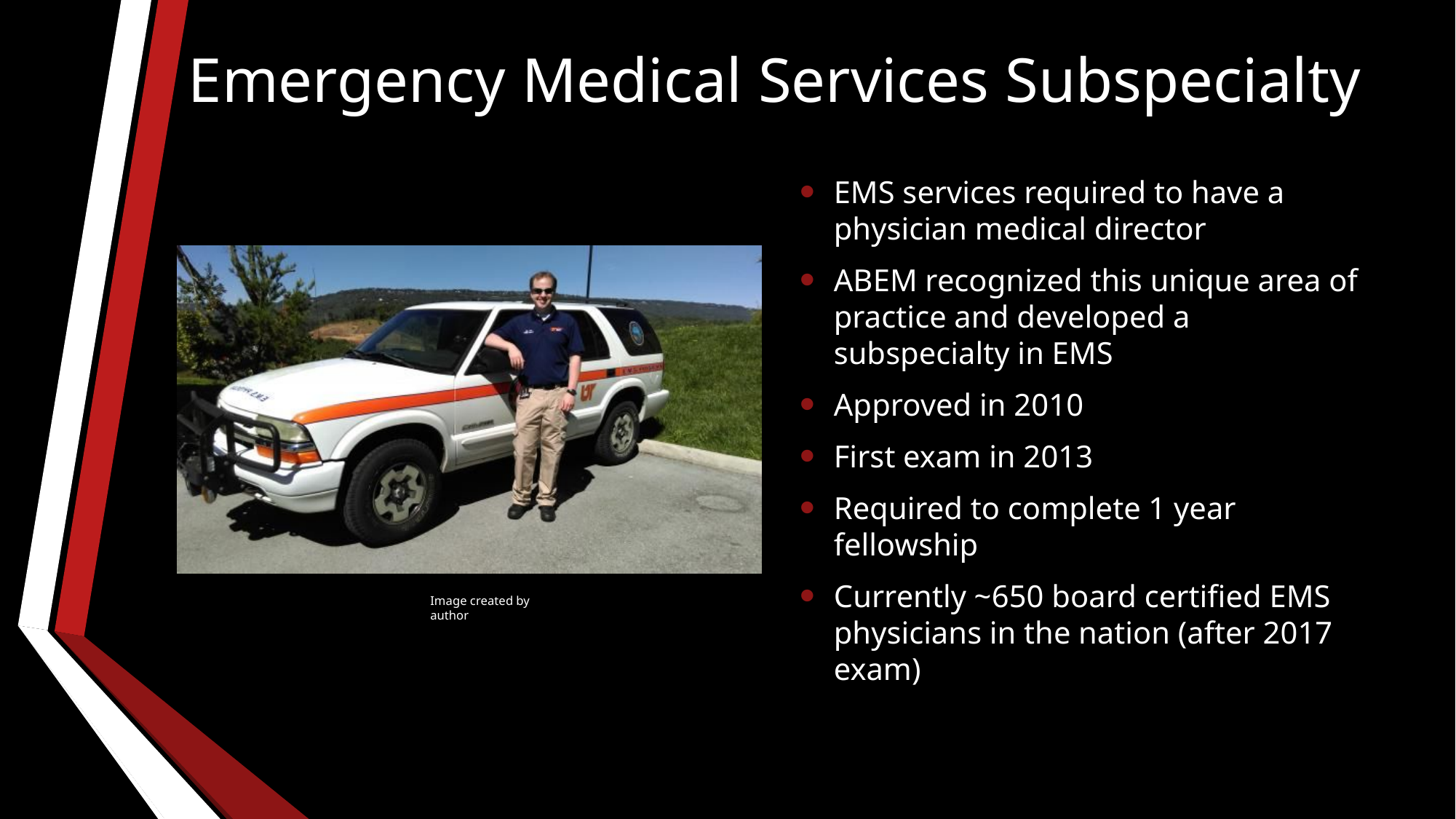

# Emergency Medical Services Subspecialty
EMS services required to have a physician medical director
ABEM recognized this unique area of practice and developed a subspecialty in EMS
Approved in 2010
First exam in 2013
Required to complete 1 year fellowship
Currently ~650 board certified EMS physicians in the nation (after 2017 exam)
Image created by author

## Slide 25
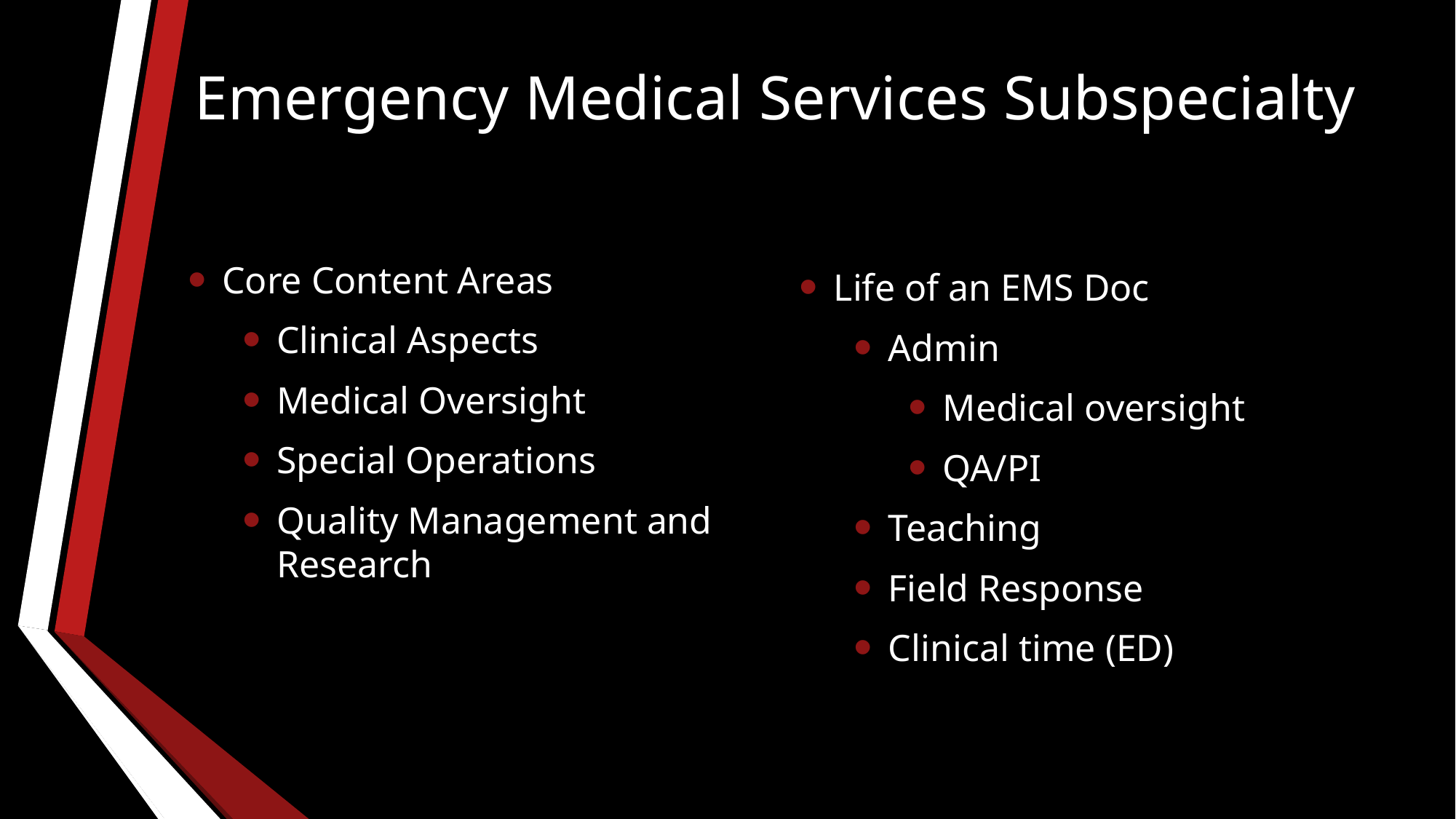

# Emergency Medical Services Subspecialty
Core Content Areas
Clinical Aspects
Medical Oversight
Special Operations
Quality Management and Research
Life of an EMS Doc
Admin
Medical oversight
QA/PI
Teaching
Field Response
Clinical time (ED)

## Slide 26
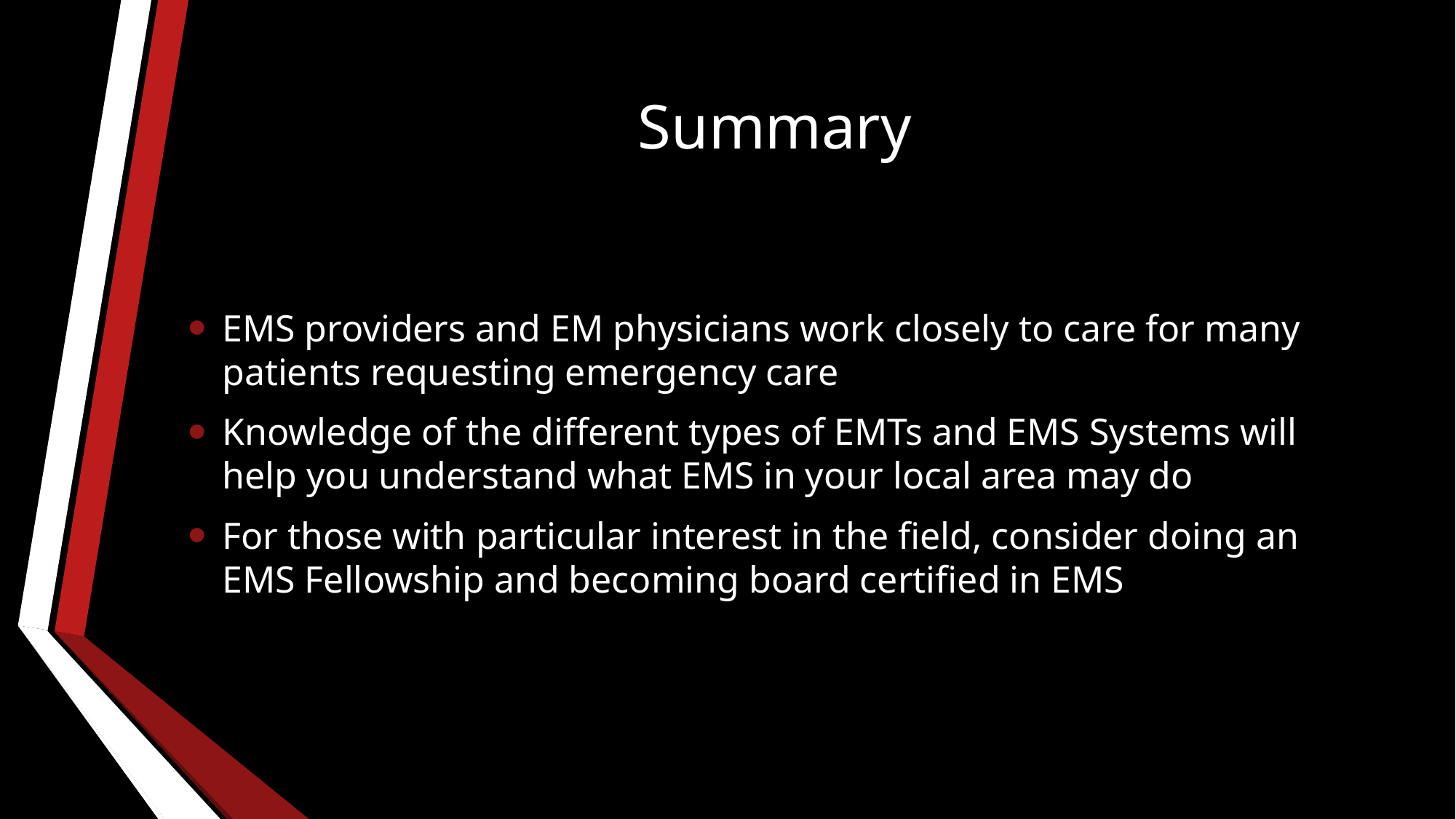

# Summary
EMS providers and EM physicians work closely to care for many patients requesting emergency care
Knowledge of the different types of EMTs and EMS Systems will help you understand what EMS in your local area may do
For those with particular interest in the field, consider doing an EMS Fellowship and becoming board certified in EMS

## Slide 27
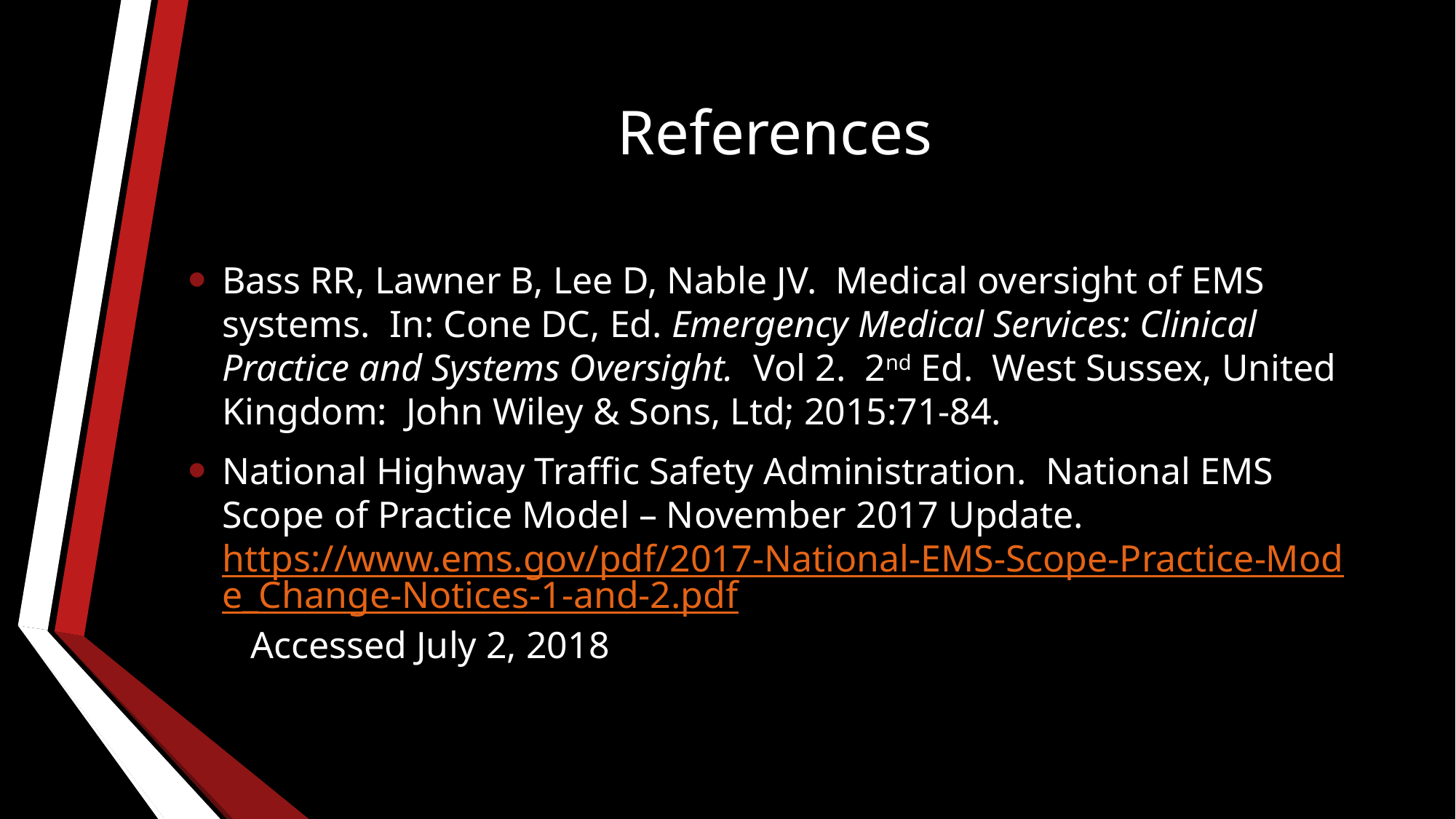

# References
Bass RR, Lawner B, Lee D, Nable JV. Medical oversight of EMS systems. In: Cone DC, Ed. Emergency Medical Services: Clinical Practice and Systems Oversight. Vol 2. 2nd Ed. West Sussex, United Kingdom: John Wiley & Sons, Ltd; 2015:71-84.
National Highway Traffic Safety Administration. National EMS Scope of Practice Model – November 2017 Update. https://www.ems.gov/pdf/2017-National-EMS-Scope-Practice-Mode_Change-Notices-1-and-2.pdf Accessed July 2, 2018
