# Supplementary material for: Online Medical Control for EMS: A Lecture and Case-Based Teaching Module
Source: MedEdPORTAL. 2020 May 15;16:10902. doi: 10.15766/mep_2374-8265.10902 (PMC7331954; doi:10.15766/mep_2374-8265.10902)
Supplement: Supplementary file 1 — OLMC Scenarios.docxIntro to EMS.pptxMedical Oversight of EMS.pptxSurvey.docxTest and Key.docxLecture Outlines.docx [file mep_2374-8265.10902-s001.zip › C. Medical Oversight of EMS.pptx]

## Slide 1
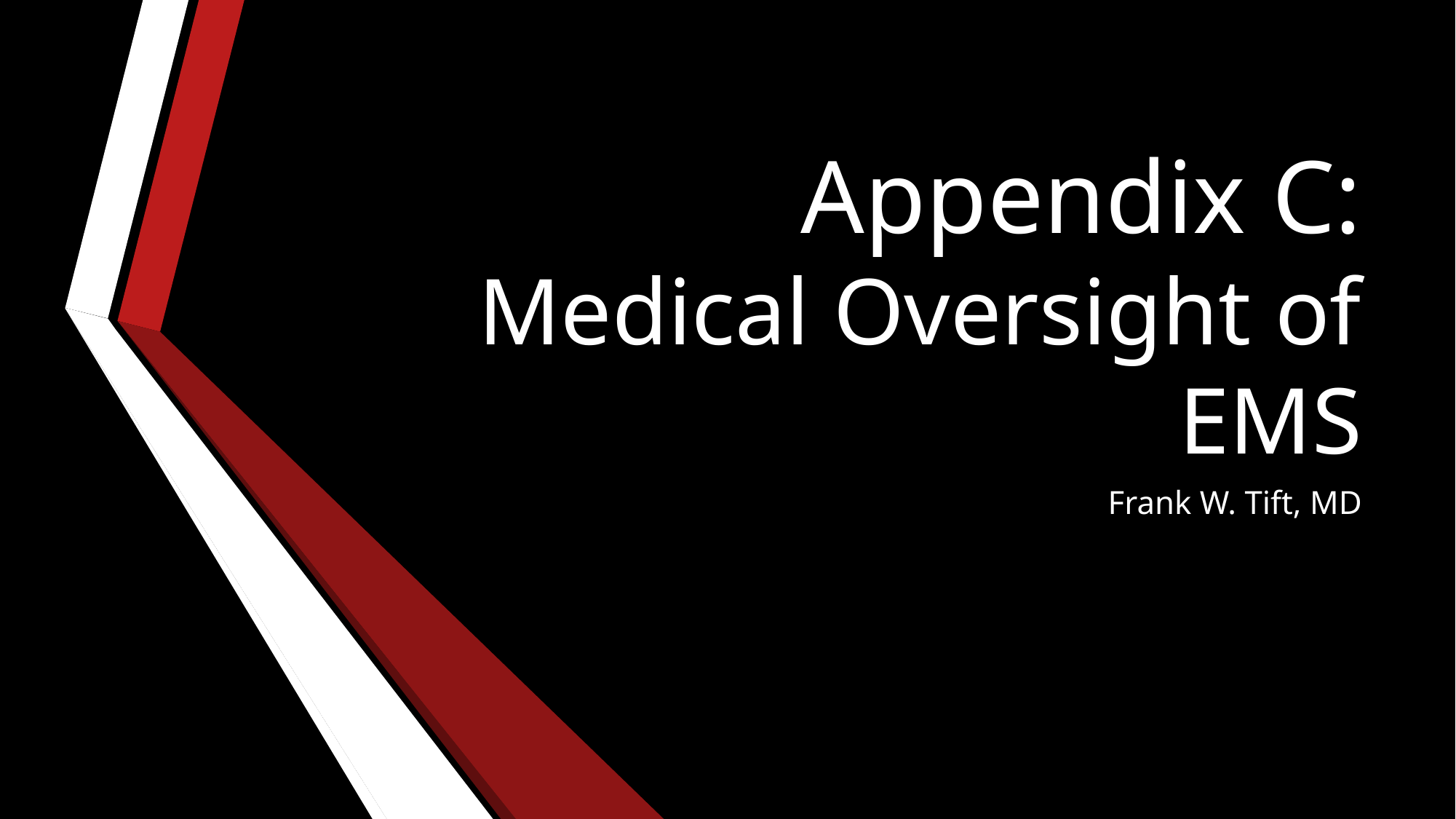

# Appendix C: Medical Oversight of EMS
Frank W. Tift, MD

## Slide 2
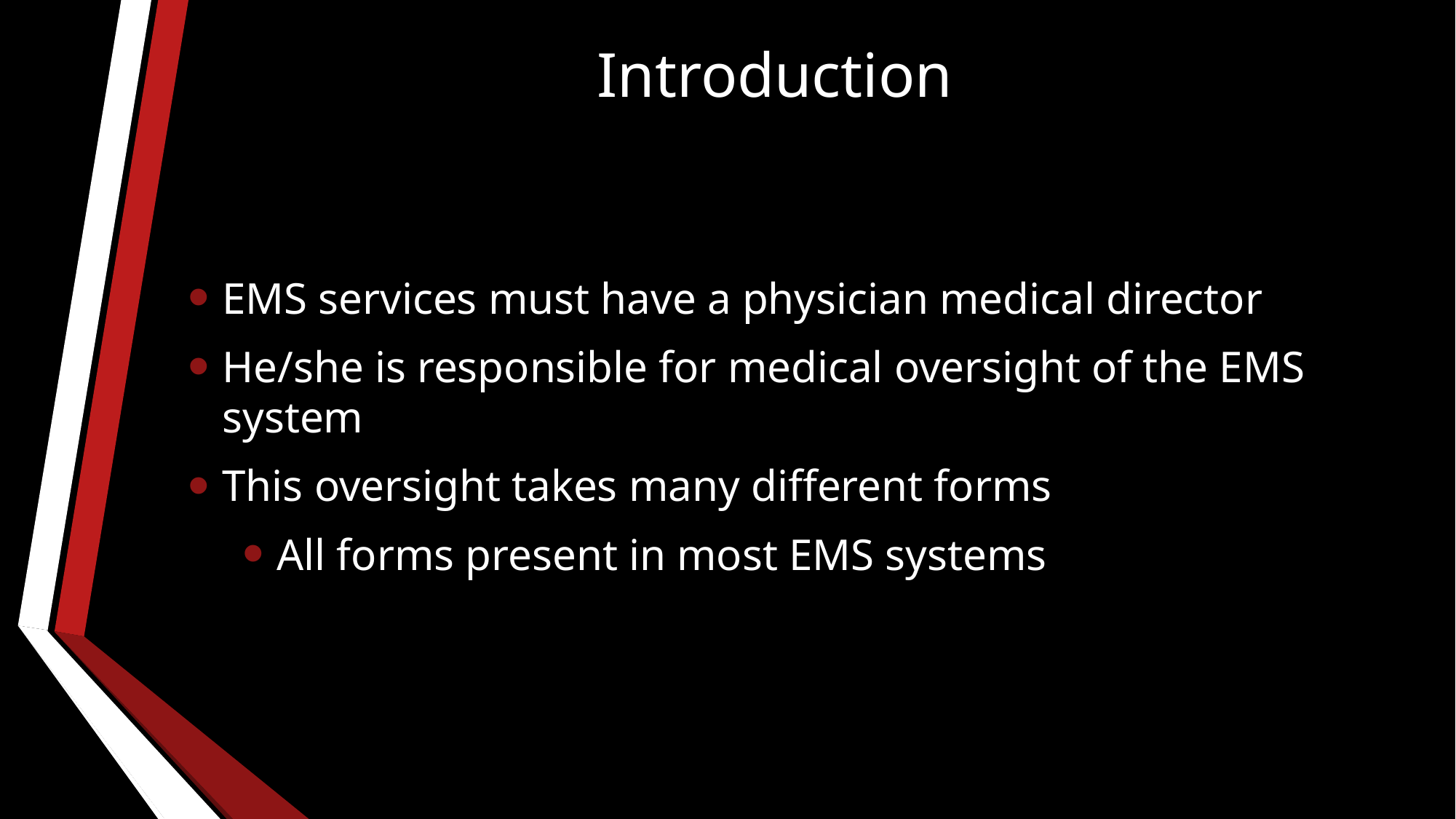

# Introduction
EMS services must have a physician medical director
He/she is responsible for medical oversight of the EMS system
This oversight takes many different forms
All forms present in most EMS systems

## Slide 3
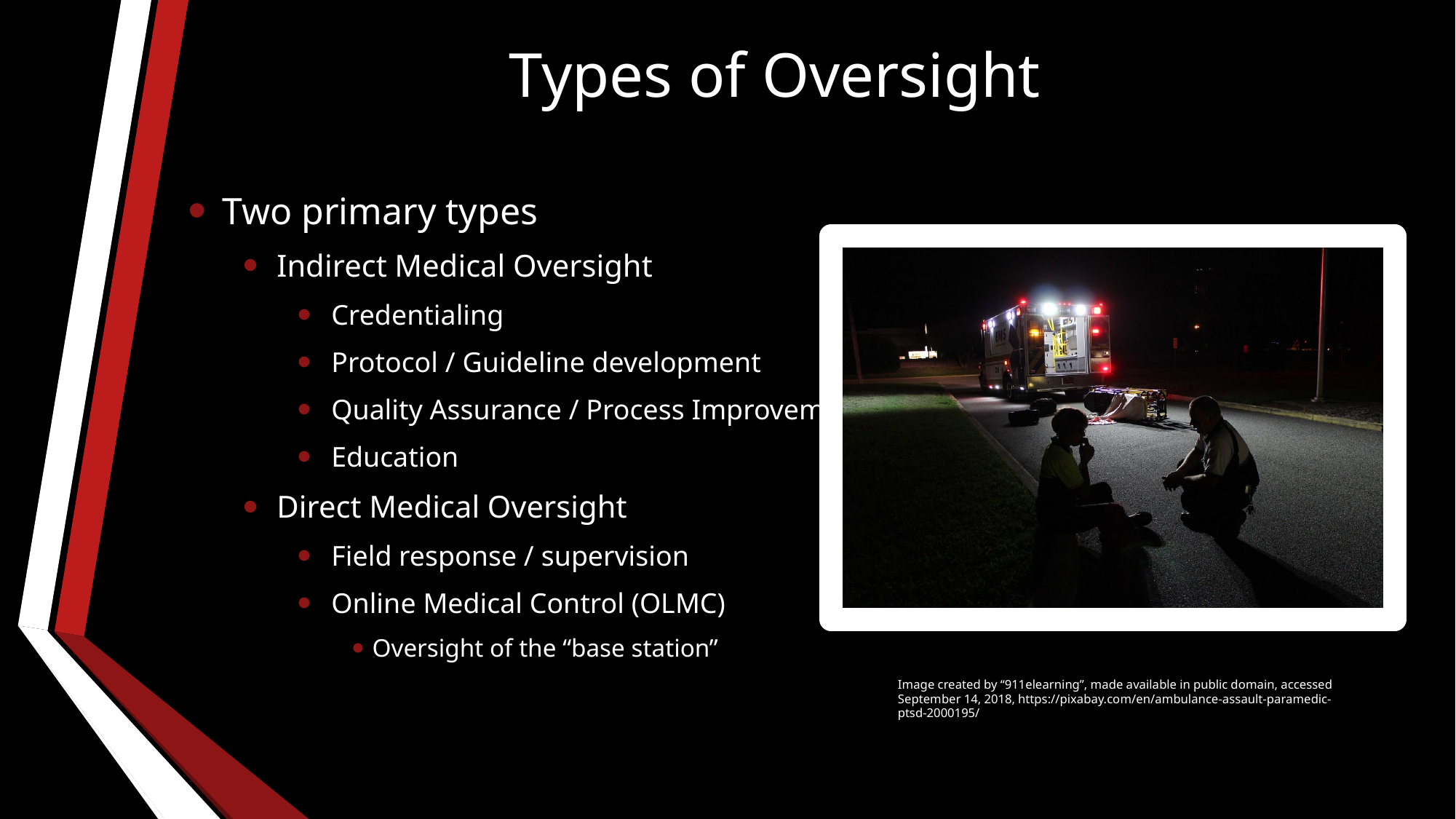

# Types of Oversight
Two primary types
Indirect Medical Oversight
Credentialing
Protocol / Guideline development
Quality Assurance / Process Improvement
Education
Direct Medical Oversight
Field response / supervision
Online Medical Control (OLMC)
Oversight of the “base station”
Image created by “911elearning”, made available in public domain, accessed September 14, 2018, https://pixabay.com/en/ambulance-assault-paramedic-ptsd-2000195/

## Slide 4
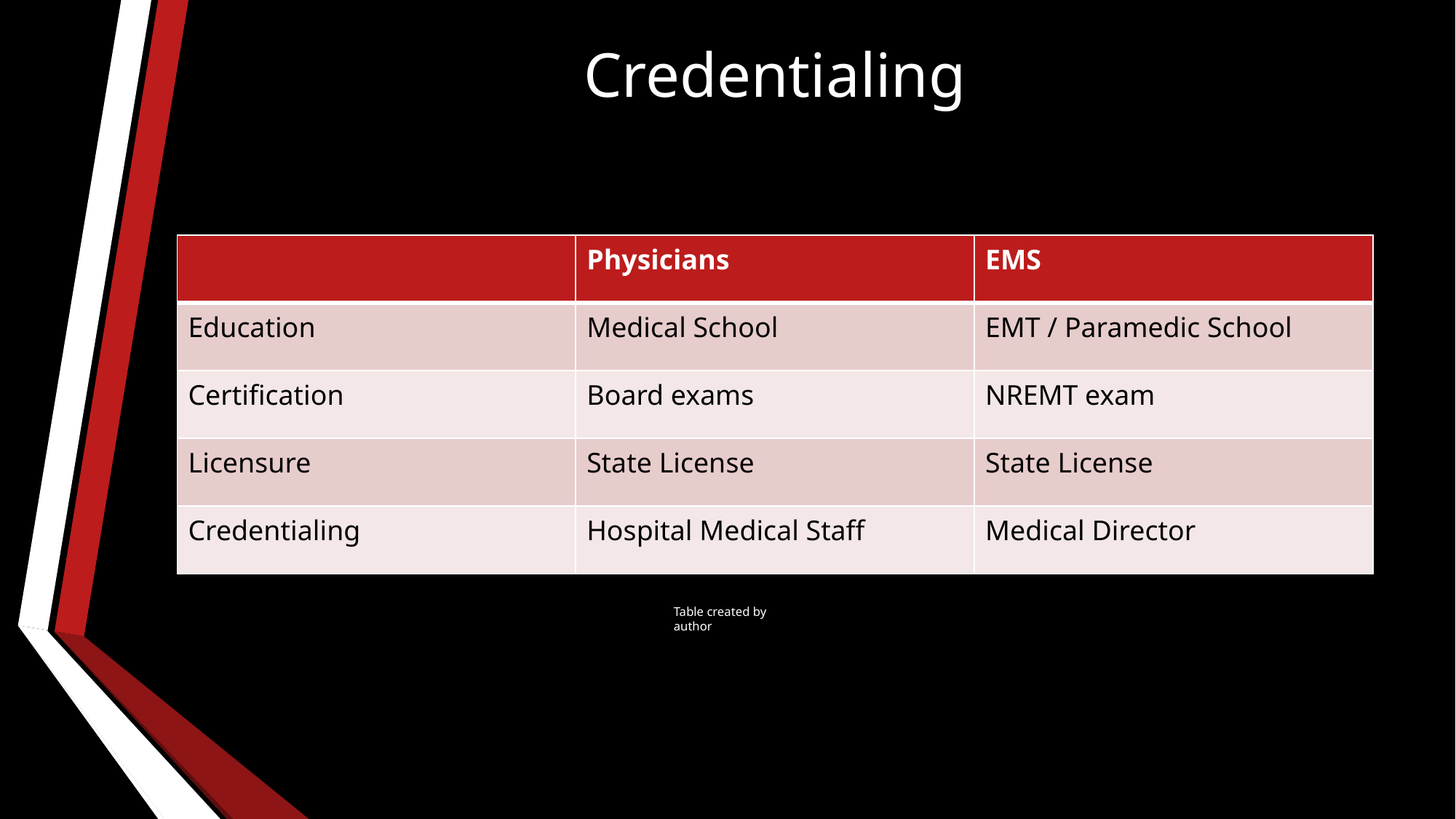

# Credentialing
| | Physicians | EMS |
| --- | --- | --- |
| Education | Medical School | EMT / Paramedic School |
| Certification | Board exams | NREMT exam |
| Licensure | State License | State License |
| Credentialing | Hospital Medical Staff | Medical Director |
Table created by author

## Slide 5
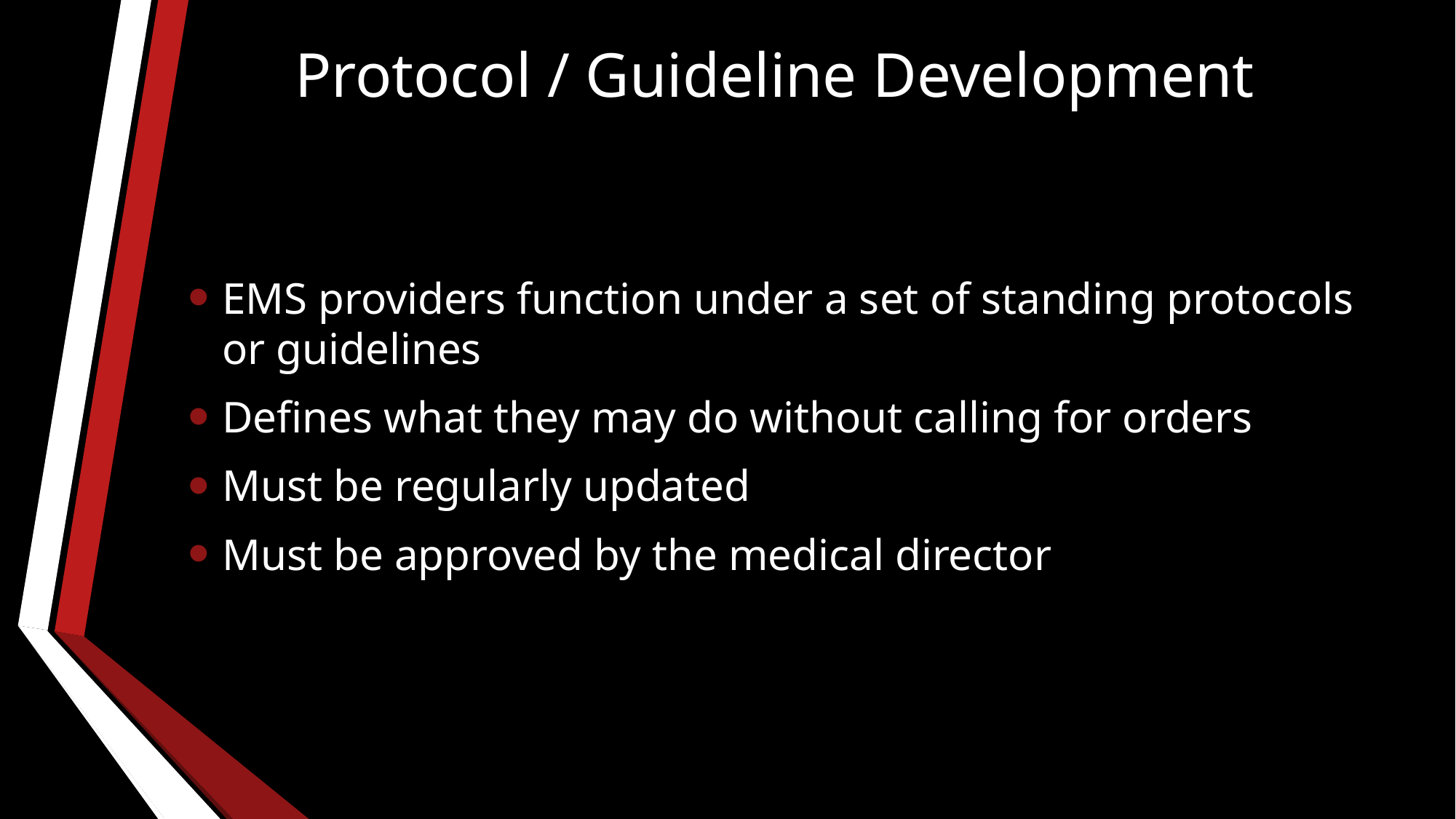

# Protocol / Guideline Development
EMS providers function under a set of standing protocols or guidelines
Defines what they may do without calling for orders
Must be regularly updated
Must be approved by the medical director

## Slide 6
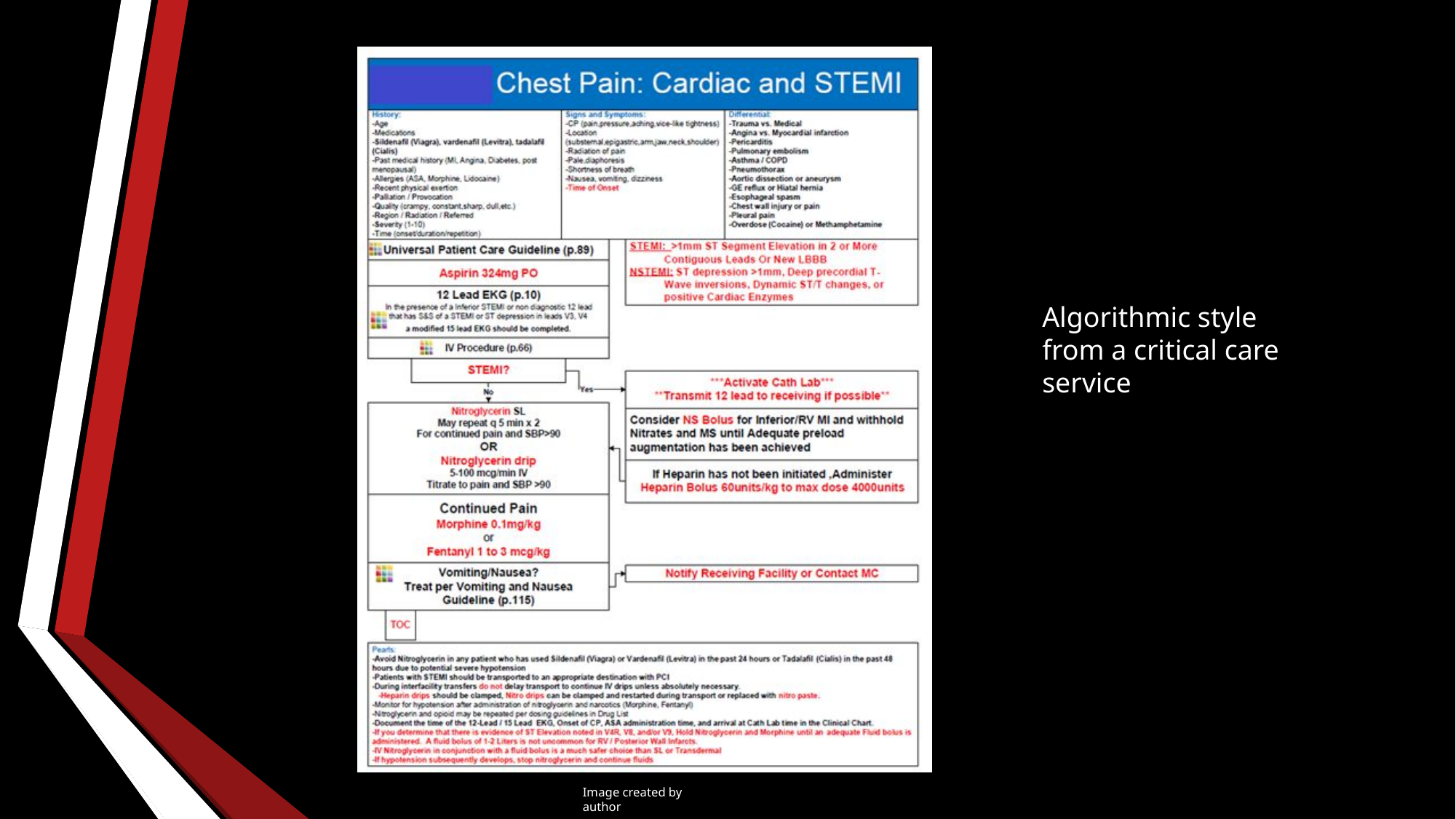

Algorithmic style from a critical care service
Image created by author

## Slide 7
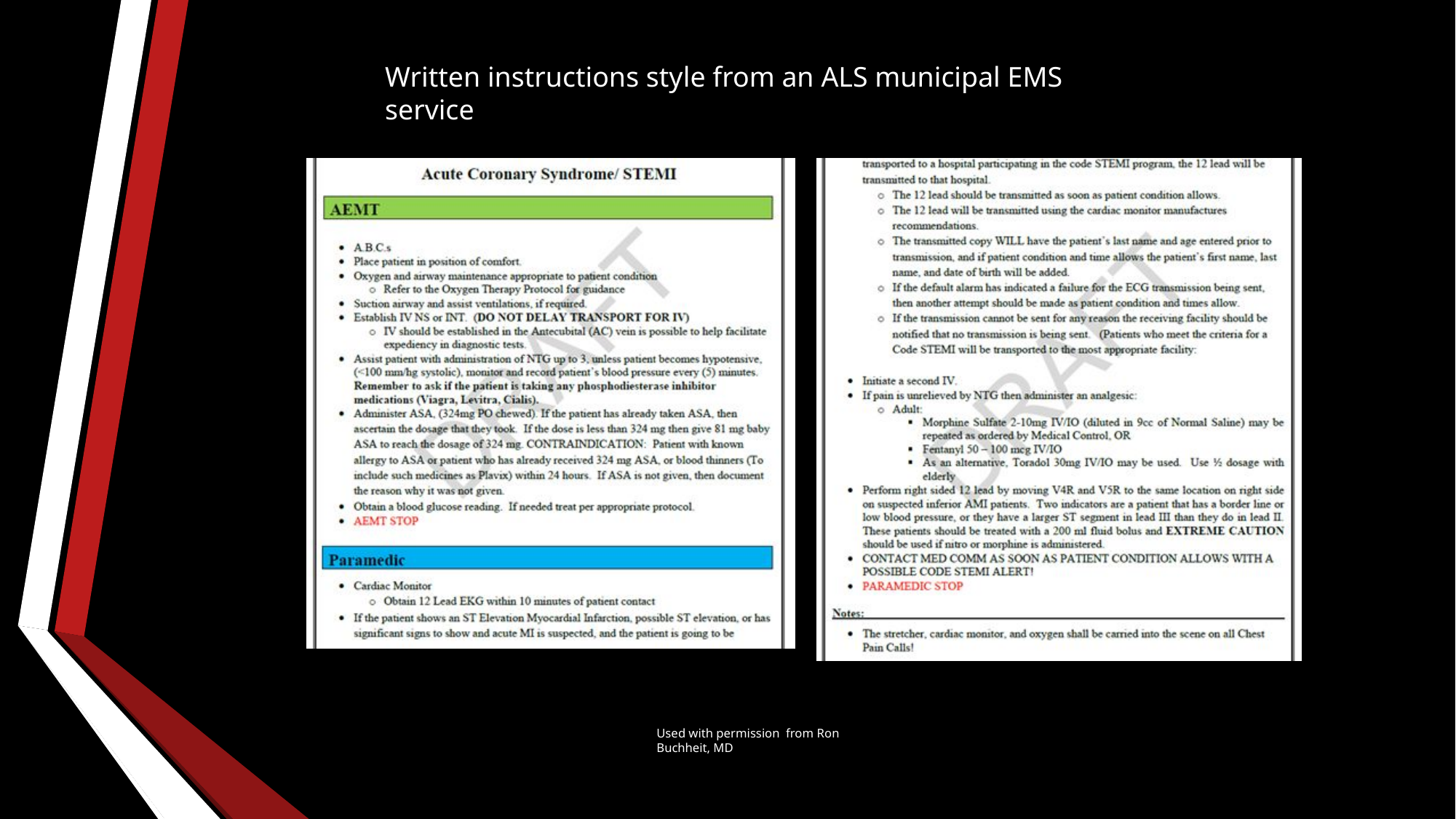

Written instructions style from an ALS municipal EMS service
Used with permission from Ron Buchheit, MD

## Slide 8
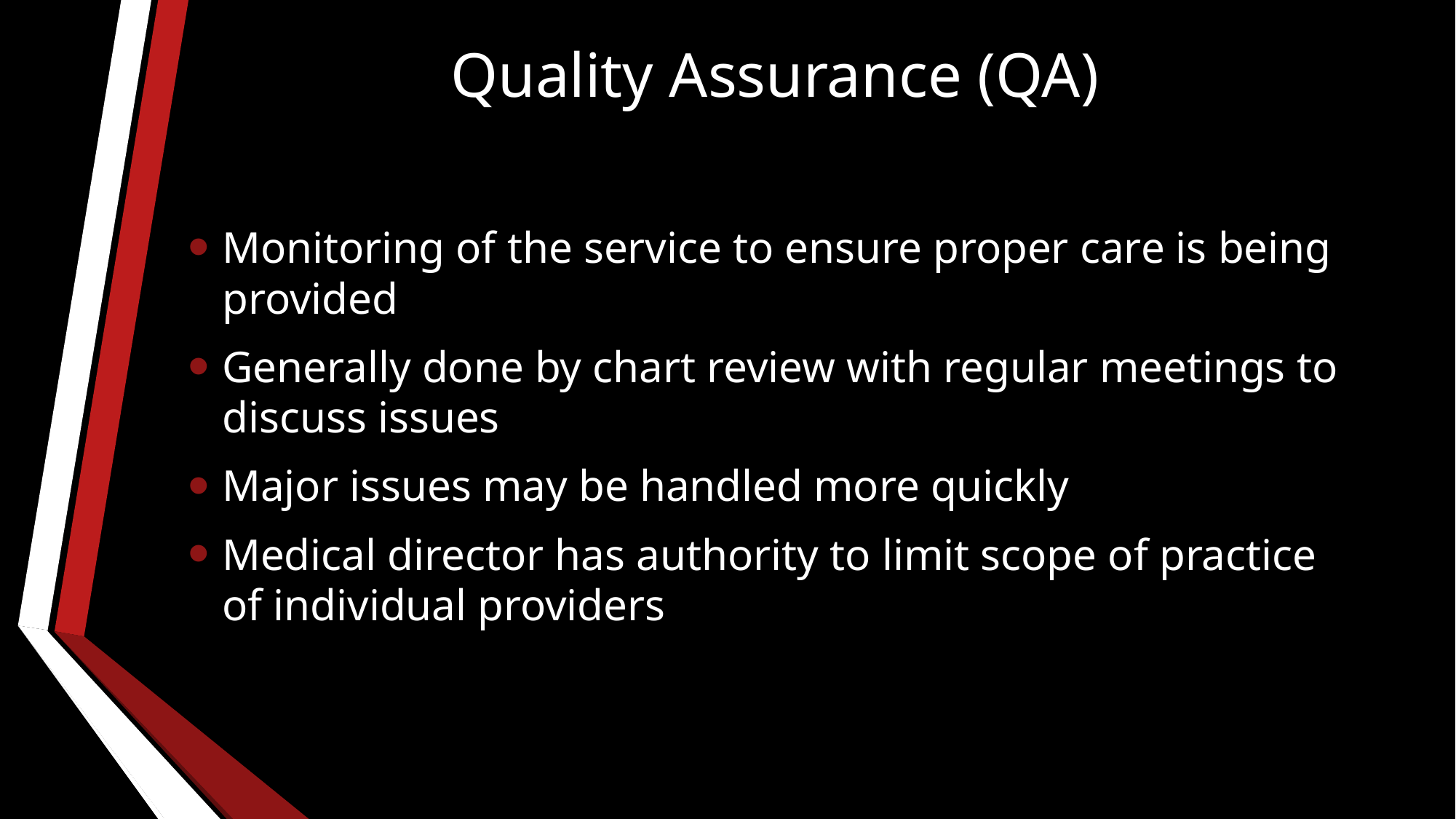

# Quality Assurance (QA)
Monitoring of the service to ensure proper care is being provided
Generally done by chart review with regular meetings to discuss issues
Major issues may be handled more quickly
Medical director has authority to limit scope of practice of individual providers

## Slide 9
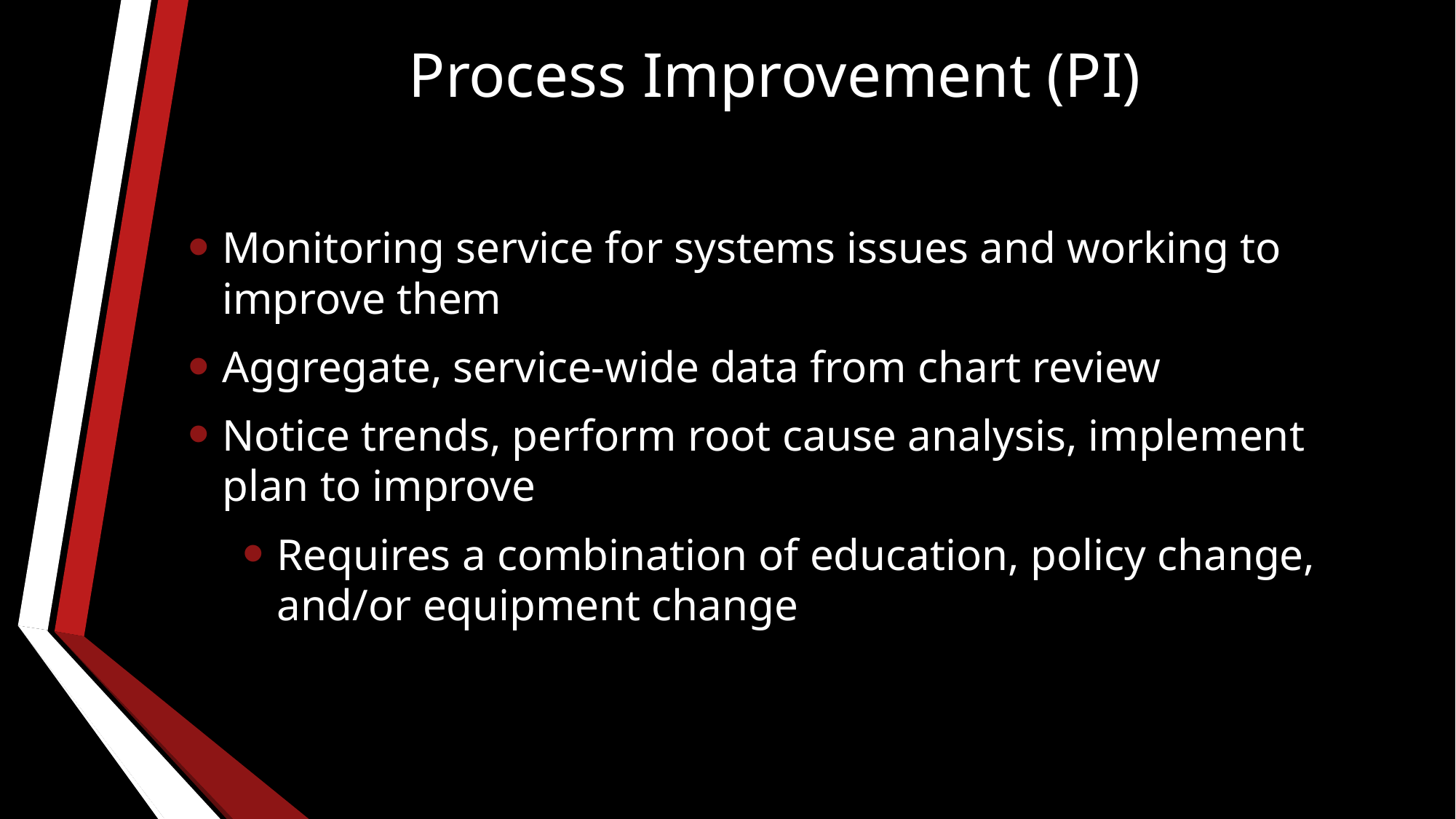

# Process Improvement (PI)
Monitoring service for systems issues and working to improve them
Aggregate, service-wide data from chart review
Notice trends, perform root cause analysis, implement plan to improve
Requires a combination of education, policy change, and/or equipment change

## Slide 10
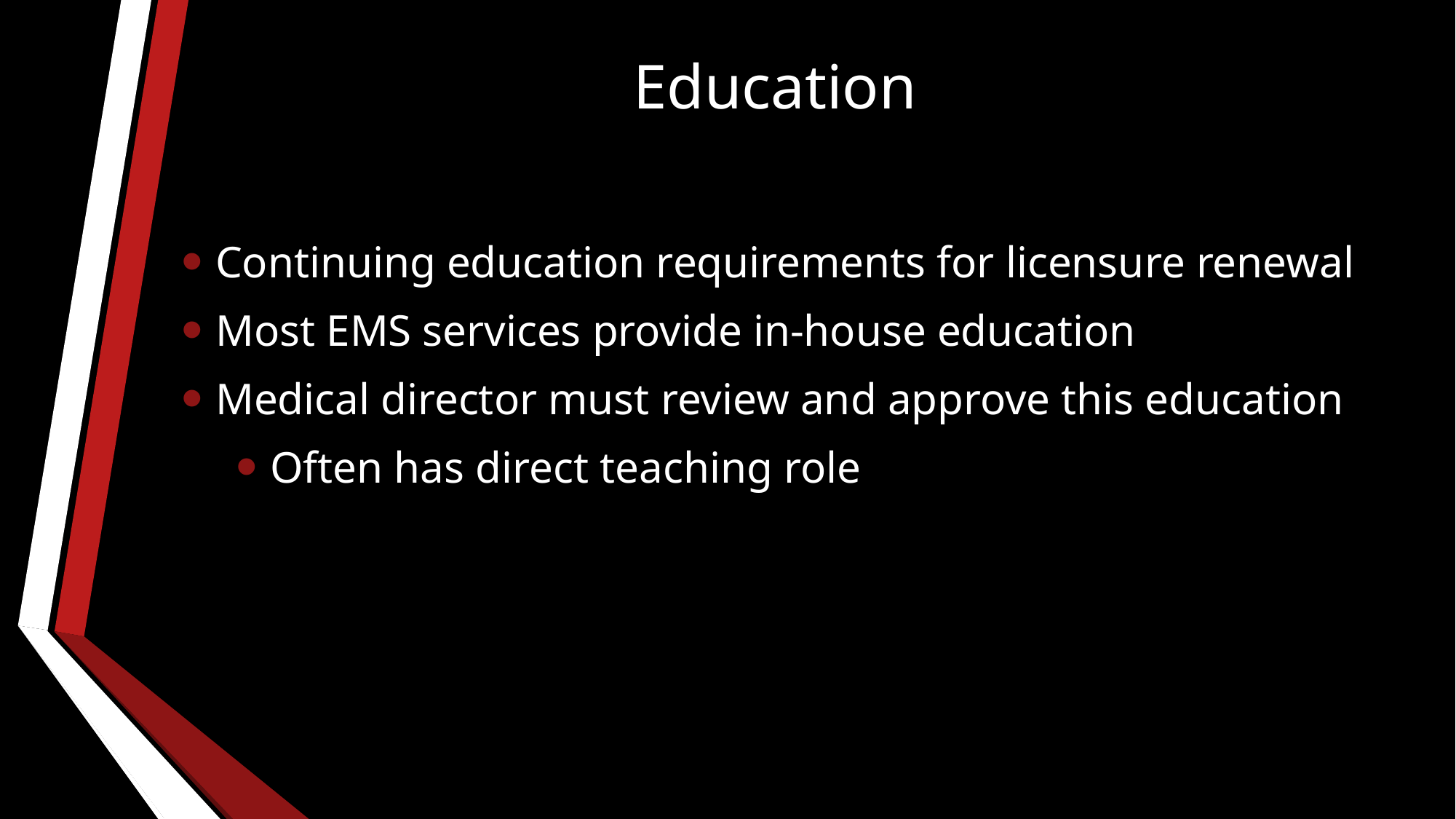

# Education
Continuing education requirements for licensure renewal
Most EMS services provide in-house education
Medical director must review and approve this education
Often has direct teaching role

## Slide 11
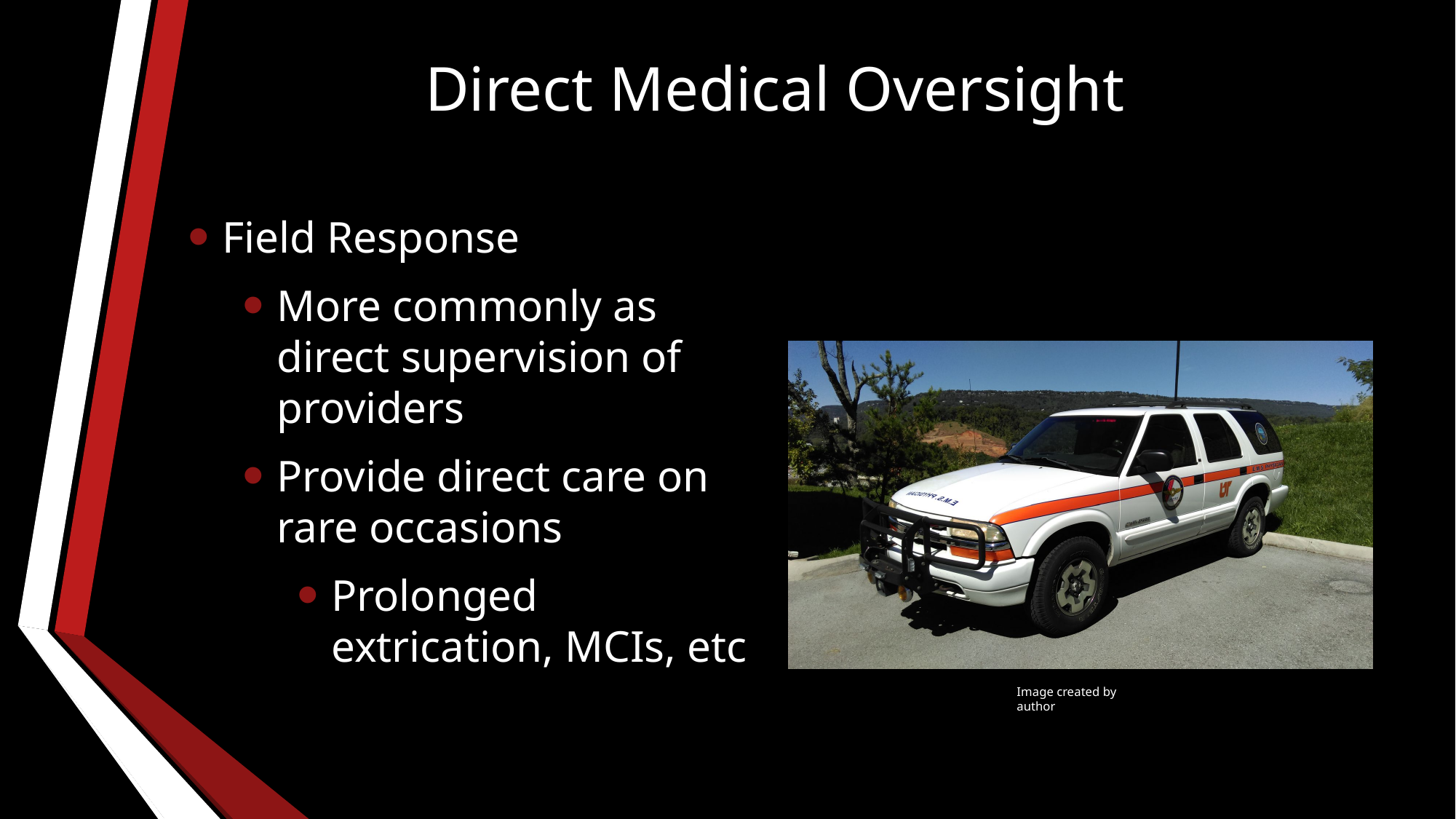

# Direct Medical Oversight
Field Response
More commonly as direct supervision of providers
Provide direct care on rare occasions
Prolonged extrication, MCIs, etc
Image created by author

## Slide 12
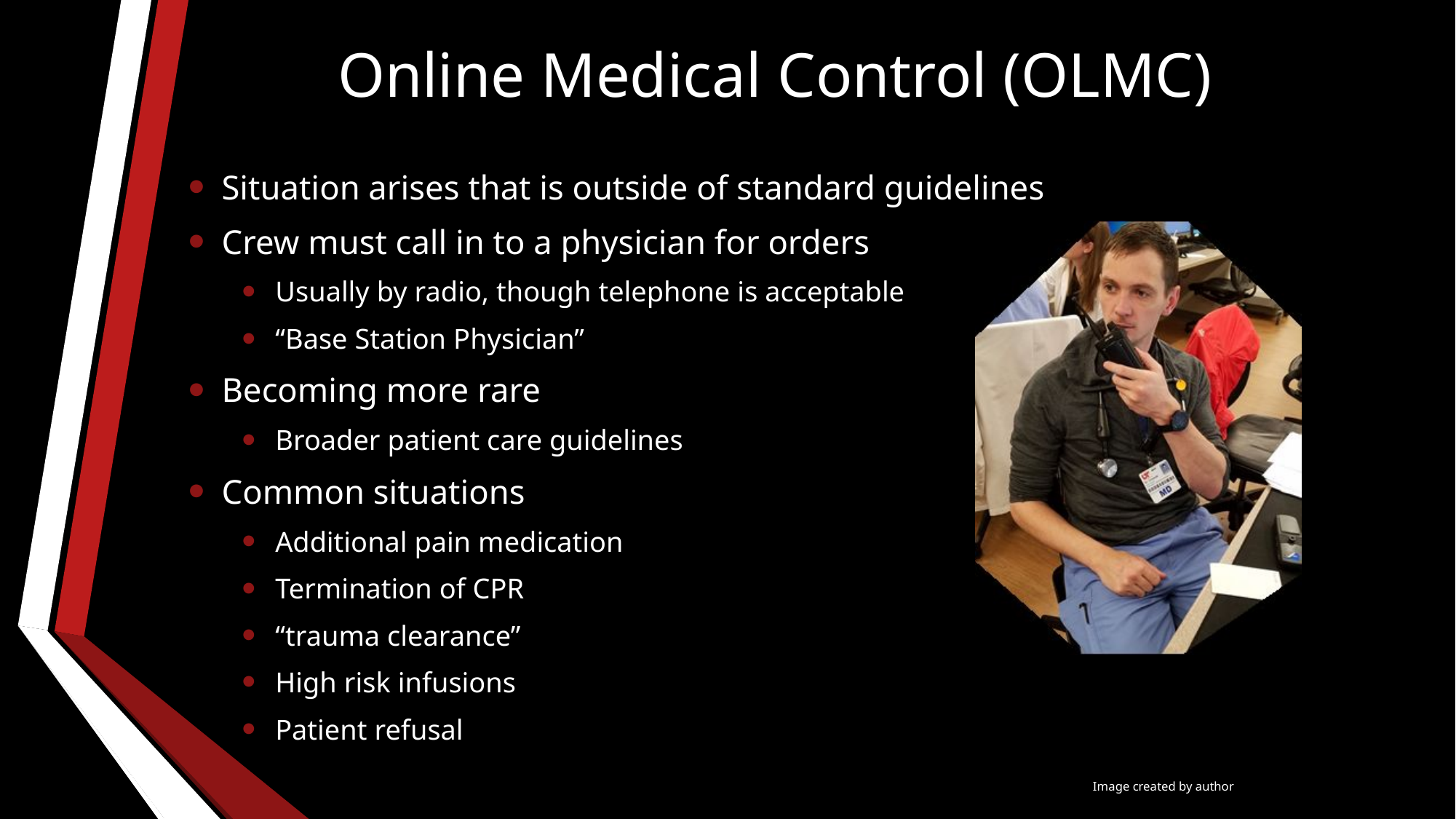

# Online Medical Control (OLMC)
Situation arises that is outside of standard guidelines
Crew must call in to a physician for orders
Usually by radio, though telephone is acceptable
“Base Station Physician”
Becoming more rare
Broader patient care guidelines
Common situations
Additional pain medication
Termination of CPR
“trauma clearance”
High risk infusions
Patient refusal
Image created by author

## Slide 13
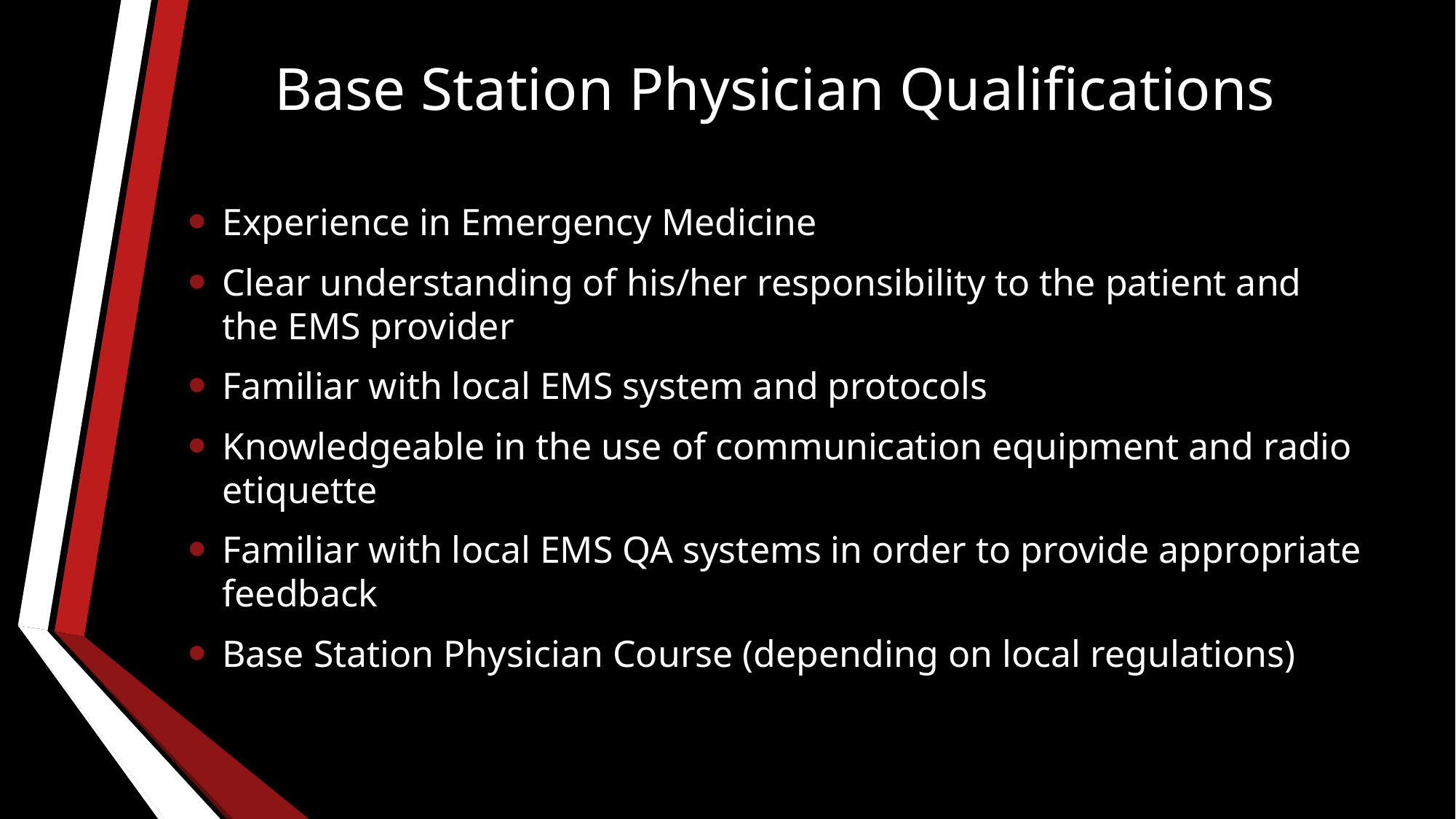

# Base Station Physician Qualifications
Experience in Emergency Medicine
Clear understanding of his/her responsibility to the patient and the EMS provider
Familiar with local EMS system and protocols
Knowledgeable in the use of communication equipment and radio etiquette
Familiar with local EMS QA systems in order to provide appropriate feedback
Base Station Physician Course (depending on local regulations)

## Slide 14
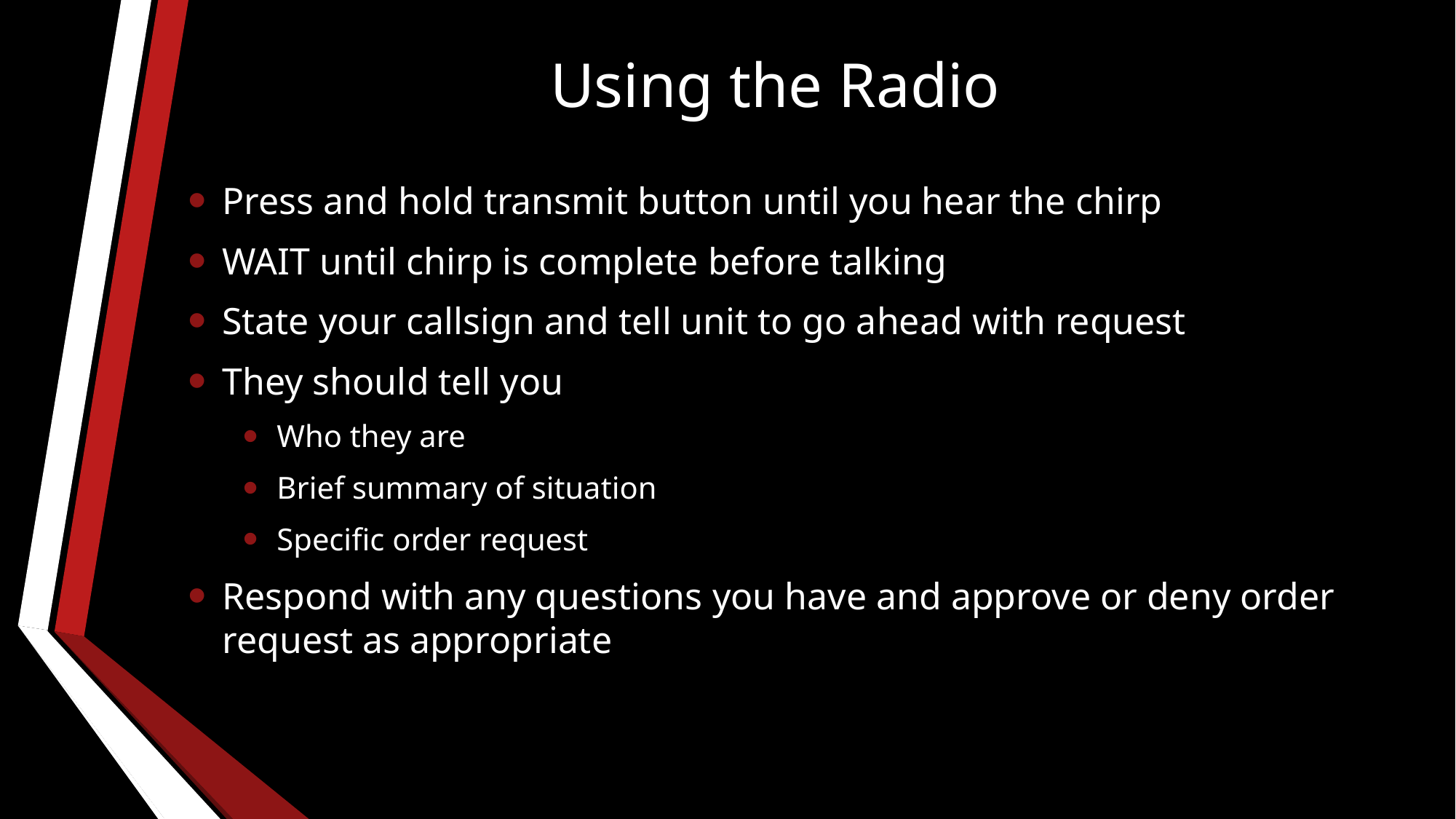

# Using the Radio
Press and hold transmit button until you hear the chirp
WAIT until chirp is complete before talking
State your callsign and tell unit to go ahead with request
They should tell you
Who they are
Brief summary of situation
Specific order request
Respond with any questions you have and approve or deny order request as appropriate

## Slide 15
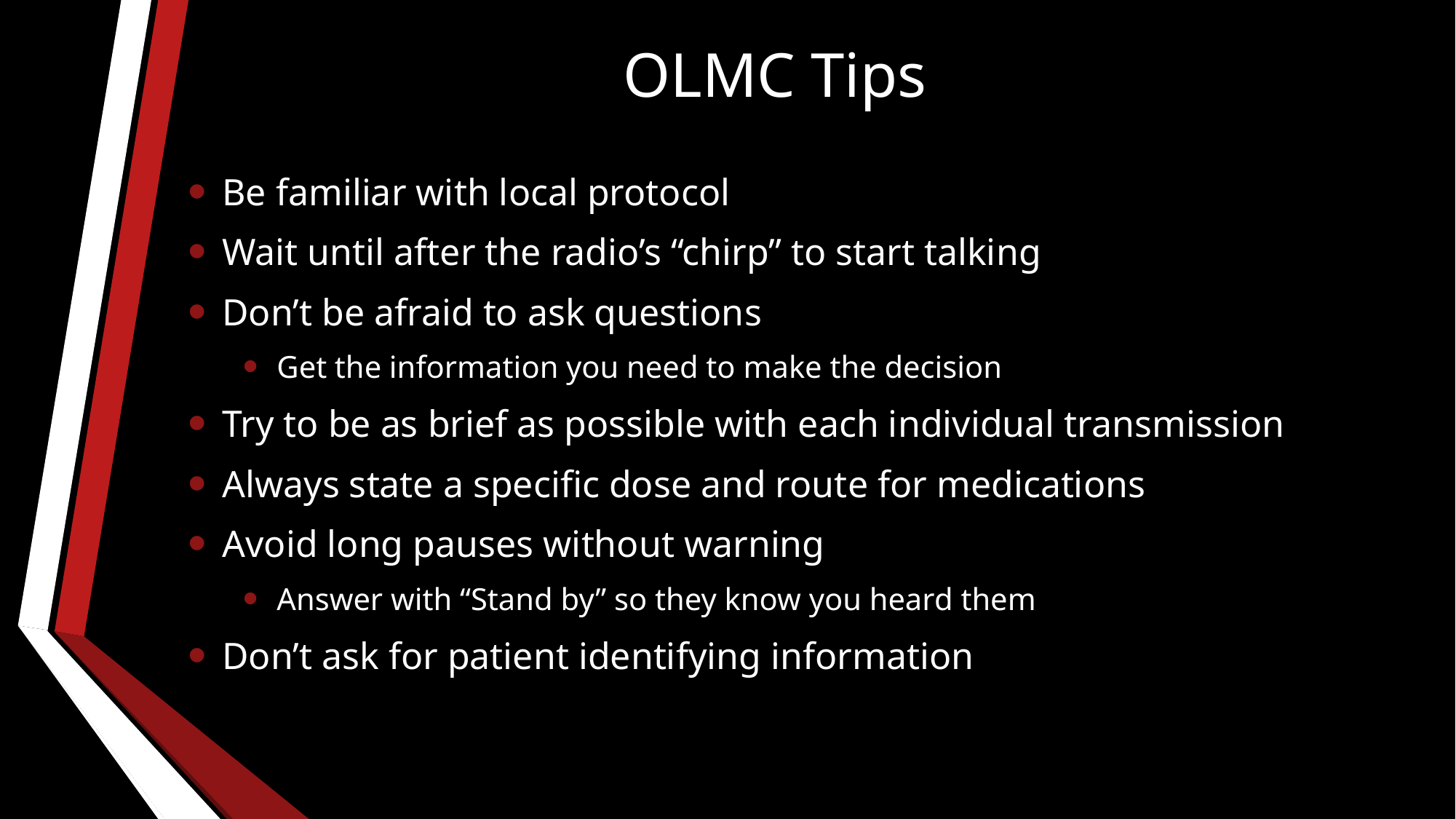

# OLMC Tips
Be familiar with local protocol
Wait until after the radio’s “chirp” to start talking
Don’t be afraid to ask questions
Get the information you need to make the decision
Try to be as brief as possible with each individual transmission
Always state a specific dose and route for medications
Avoid long pauses without warning
Answer with “Stand by” so they know you heard them
Don’t ask for patient identifying information

## Slide 16
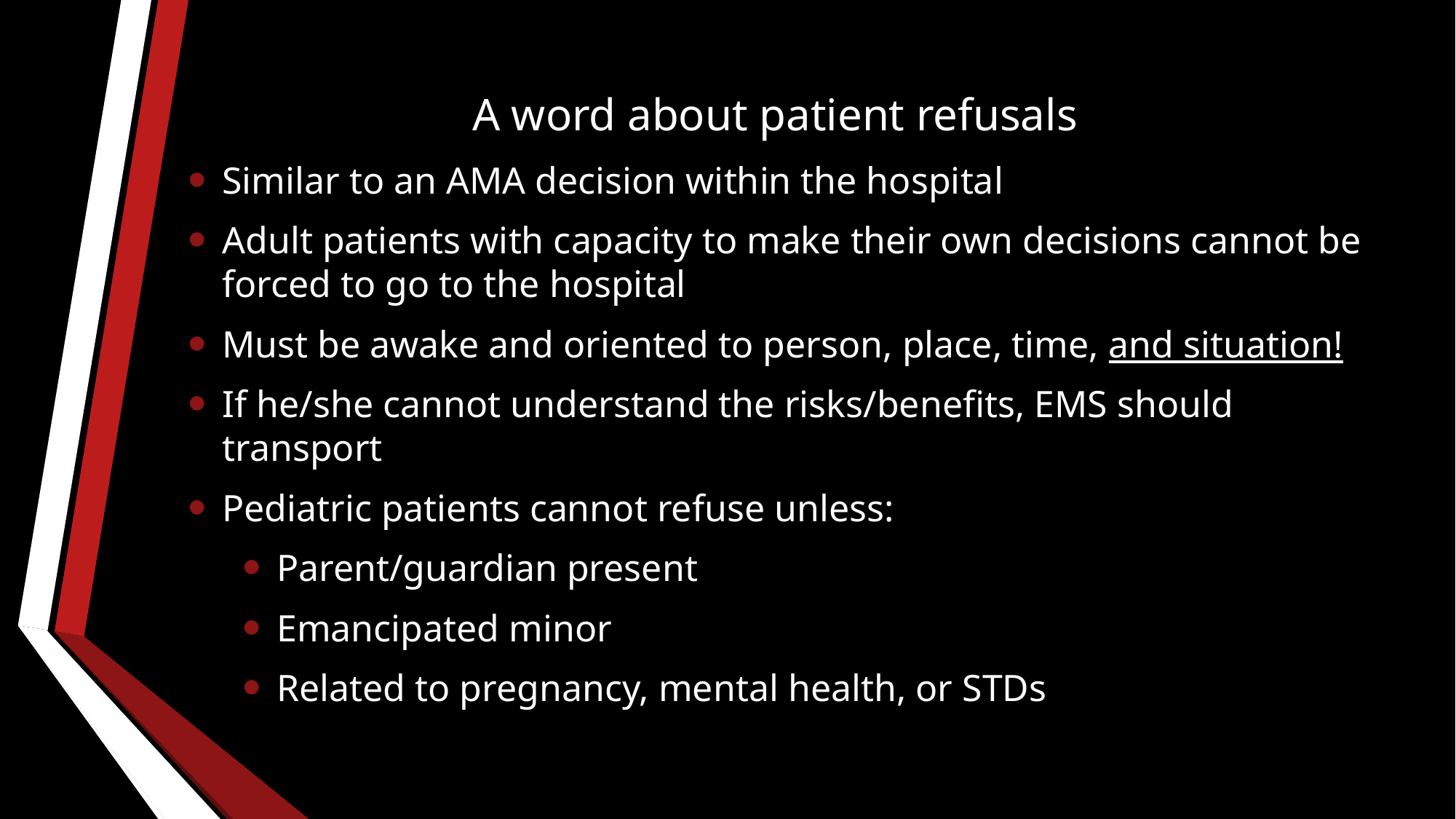

# A word about patient refusals
Similar to an AMA decision within the hospital
Adult patients with capacity to make their own decisions cannot be forced to go to the hospital
Must be awake and oriented to person, place, time, and situation!
If he/she cannot understand the risks/benefits, EMS should transport
Pediatric patients cannot refuse unless:
Parent/guardian present
Emancipated minor
Related to pregnancy, mental health, or STDs

## Slide 17
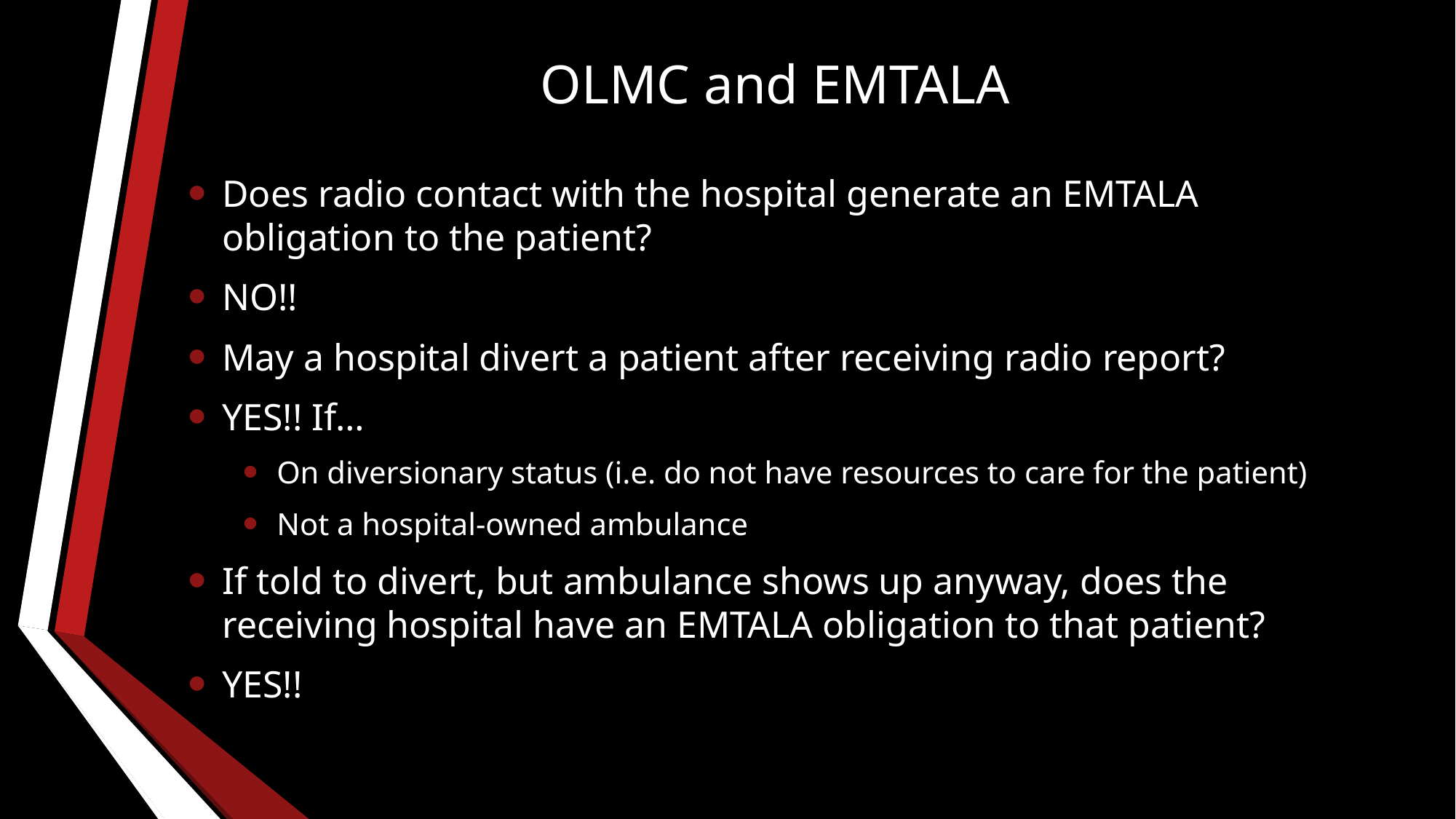

# OLMC and EMTALA
Does radio contact with the hospital generate an EMTALA obligation to the patient?
NO!!
May a hospital divert a patient after receiving radio report?
YES!! If…
On diversionary status (i.e. do not have resources to care for the patient)
Not a hospital-owned ambulance
If told to divert, but ambulance shows up anyway, does the receiving hospital have an EMTALA obligation to that patient?
YES!!

## Slide 18
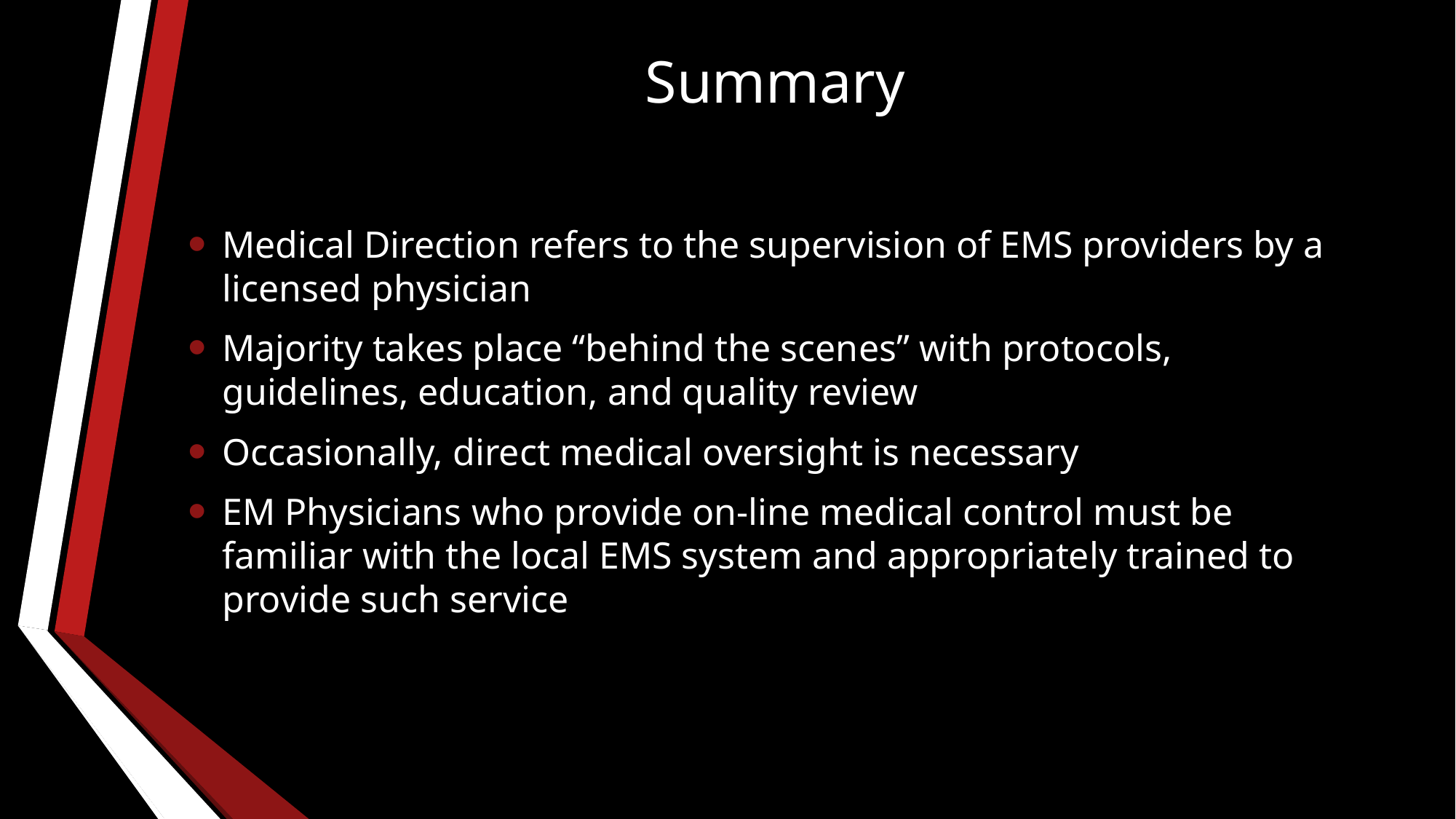

# Summary
Medical Direction refers to the supervision of EMS providers by a licensed physician
Majority takes place “behind the scenes” with protocols, guidelines, education, and quality review
Occasionally, direct medical oversight is necessary
EM Physicians who provide on-line medical control must be familiar with the local EMS system and appropriately trained to provide such service

## Slide 19
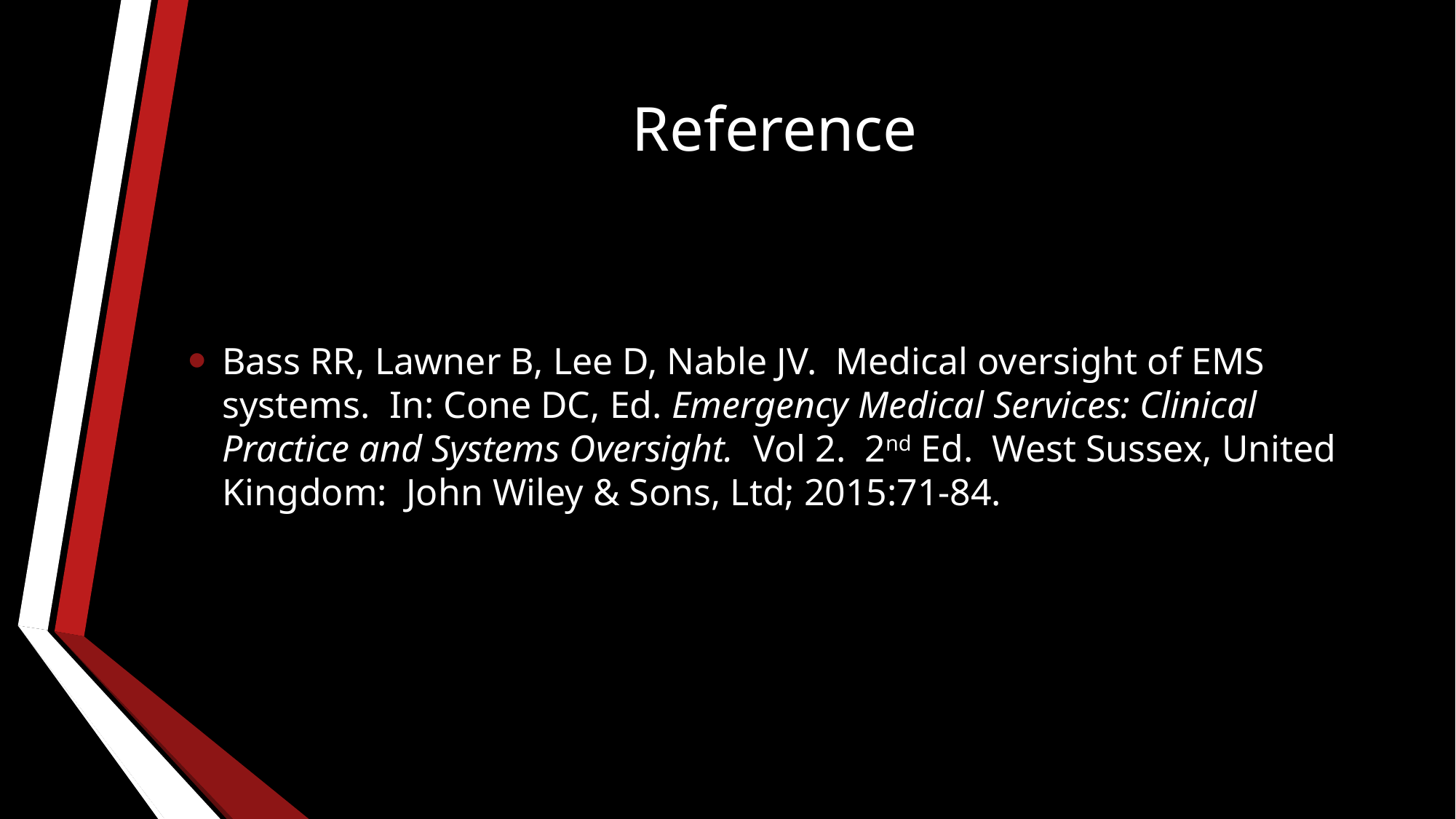

# Reference
Bass RR, Lawner B, Lee D, Nable JV. Medical oversight of EMS systems. In: Cone DC, Ed. Emergency Medical Services: Clinical Practice and Systems Oversight. Vol 2. 2nd Ed. West Sussex, United Kingdom: John Wiley & Sons, Ltd; 2015:71-84.
