# Supplementary material for: Online Medical Control for EMS: A Lecture and Case-Based Teaching Module
Source: MedEdPORTAL. 2020 May 15;16:10902. doi: 10.15766/mep_2374-8265.10902 (PMC7331954; doi:10.15766/mep_2374-8265.10902)
Supplement: Supplementary file 1 — OLMC Scenarios.docxIntro to EMS.pptxMedical Oversight of EMS.pptxSurvey.docxTest and Key.docxLecture Outlines.docx [file mep_2374-8265.10902-s001.zip › D. Survey.docx]

**Appendix D:**

**Basic EMS and Online Medical Command (OLMC) Survey**

Please answer the following questions on a scale from 1 to 5

Strongly Disagree Neutral Strongly Agree

1 2 3 4 5

I understand the different levels of certification of prehospital providers

1 2 3 4 5

I understand the difference between a basic life support (BLS) and an advanced life support (ALS) ambulance

1 2 3 4 5

I have a good working knowledge of the medical capabilities of a standard ALS ambulance

1 2 3 4 5

I have a good working knowledge of the different types of EMS services

1 2 3 4 5

I feel comfortable providing online medical command (orders over radio/telephone) to prehospital providers

1 2 3 4 5

What is your training level?

Medical Student PGY-1 PGY-2 PGY-3 Attending
