# Supplementary material for: Online Medical Control for EMS: A Lecture and Case-Based Teaching Module
Source: MedEdPORTAL. 2020 May 15;16:10902. doi: 10.15766/mep_2374-8265.10902 (PMC7331954; doi:10.15766/mep_2374-8265.10902)
Supplement: Supplementary file 1 — OLMC Scenarios.docxIntro to EMS.pptxMedical Oversight of EMS.pptxSurvey.docxTest and Key.docxLecture Outlines.docx [file mep_2374-8265.10902-s001.zip › E. Test and Key.docx]

**Appendix E:**

Name __________________________ Training Level ____________________

**Basic EMS and Online Medical Command (OLMC) Knowledge Test**

1. What is the minimum certification level required to start an IV? C

1. Emergency Medical Responder (EMR)
2. Emergency Medical Technician (EMT)
3. Advanced Emergency Medical Technician (AEMT)
4. Paramedic

2. An ambulance with a crew consisting of two Advanced EMTs is considered an Advanced Life Support (ALS) truck: F
 True or False? (Circle one)

3. An Emergency Department who has received radio communications from an EMS unit has generated an EMTALA obligation to the patient being transported: F
 True or False? (Circle one)

4. What is the minimum certification level required to transport a patient in an ambulance? B

1. Emergency Medical Responder (EMR)
2. Emergency Medical Technician (EMT)
3. Advanced Emergency Medical Technician (AEMT)
4. Paramedic

5. What model of EMS service is fully funded and operated by the local government as its own department? D

1. Fire-based Model
2. Public Utility Model
3. Private Model
4. Third Service Model

6. An AEMT may give a patient morphine: F
 True or False? (Circle one)

7. Which of the following is an example of proper use of the radio: C

1. Press and release the transmit button, then start talking
2. Ask all questions you have within a single, long transmission
3. Wait until the “chirp” has stopped before talking
4. Ask for the patient’s name and birth date so you can check their chart

8. Which of the following is TRUE regarding a Private Model EMS service: D

1. The local government completely funds the service, while the private company provides employees, equipment, and runs day-to-day operations
2. They are often more financially responsible
3. They often complete non-emergency transports in addition to 911 calls
4. Both B & C
5. None of the above

9. Fire departments often provide first responder services even if they are not the primary EMS service: T
 True or False? (Circle one)

10. What is the minimum level of certification required to intubate a patient? D

1. Emergency Medical Responder (EMR)
2. Emergency Medical Technician (EMT)
3. Advanced Emergency Medical Technician (AEMT)
4. Paramedic

11. A BLS ambulance bringing a patient with shortness of breath to the hospital will be able to provide a 12-lead ECG: F
 True or False? (Circle one)

12. If after receiving an EMS radio report, you determine that your facility cannot adequately care for the patient, you may ask the transporting unit to divert to a different facility: T
 True or False? (Circle one)

13. The qualifications of a base station physician include which of the following: E

1. Experience in Emergency Medicine
2. Knowledge of the local EMS system and protocols
3. Familiarity with the proper use of communications equipment
4. Clear understanding of his/her role and responsibility to the patient and the EMS provider
5. All of the above

14. Which of the following are TRUE about a patient’s refusal of EMS services and transport in the field: E

1. An adult patient with capacity to make decisions cannot be forced to go to the hospital
2. A patient who doesn’t seem to understand the situation, yet who is awake and oriented to person, place, and time, may refuse care
3. A parent or guardian is required for pediatric refusal in most situations
4. All of the above
5. A & C only

15. In an EMS system which utilizes “tiered response”, patients may be transported to the Emergency Department by a BLS ambulance: T
 True or False? (Circle one)

16. Which of the following are benefits of a fire-based EMS system? E

1. Infrastructure is already in place
2. Government services gain no revenue from patient transports
3. The needs of the EMS division are often given higher priority than the needs of the remainder of the fire department
4. EMS is more easily integrated into a disaster response
5. A & D only

17. The base station physician should state a specific dose when authorizing the use of a medication: T
 True or False? (Circle one)

18. Which of the following are circumstances in which it is appropriate to provide orders to discontinue CPR? E

1. Unknown downtime, initial rhythm of VT with subsequent defibrillation, now in asystole for 5 minutes
2. Female of child-bearing age who appears to be pregnant and is in asystole
3. Patient in continued PEA after 3 rounds of CPR and drugs who is on the fifth floor of a building under construction with no elevator
4. All of the above
5. None of the above

19. Whom should you contact at an EMS service with a complaint about a prehospital provider’s care? D

1. The medical director
2. The QA coordinator
3. The training coordinator
4. A or B

20. The base station physician should not hesitate to ask for more information from a crew requesting orders if needed for appropriate medical decision making: T
 True or False? (Circle one)
