# Supplementary material for: Online Medical Control for EMS: A Lecture and Case-Based Teaching Module
Source: MedEdPORTAL. 2020 May 15;16:10902. doi: 10.15766/mep_2374-8265.10902 (PMC7331954; doi:10.15766/mep_2374-8265.10902)
Supplement: Supplementary file 1 — OLMC Scenarios.docxIntro to EMS.pptxMedical Oversight of EMS.pptxSurvey.docxTest and Key.docxLecture Outlines.docx [file mep_2374-8265.10902-s001.zip › F. Lecture Outlines.docx]

**Intro to EMS**
Lecture Outline

- Introduction
- EMS Personnel Types
  - There are 4 levels of training in the National Scope of Practice Model
    - Emergency Medical Responder (EMR)
    - Emergency Medical Technician (EMT)
    - Advanced Emergency Medical Technician (AEMT)
    - Paramedic
  - Some states also recognize Critical Care Paramedic as a training level above paramedic
  - Emergency Medical Responder (EMR)
    - Education: short course
    - Scope: basic first aid and AEDs
    - Not allowed to transport patients
  - Emergency Medical Technician (EMT)
    - Education: one semester with clinical shifts
    - Scope: EMR + aspirin, glucose, and assist patients with their own meds
  - Advanced Emergency Medical Technician (AEMT)
    - Education: additional semester with clinical shifts
    - Scope: EMT + supraglottic airways, IV/IO, IVF, low risk meds
  - Paramedic
    - Education: additional 3 semesters past AEMT with large amount of clinical shifts
    - Scope: AEMT + intubation, needle cricothyrotomy, needle thoracostomy, manual cardiac rhythm/ECG and defib, most meds
- EMS Systems
  - 911 system
    - PSAP = public safety access point.
    - Call taker and dispatcher usually in same location or same person
    - Can give pre-arrival instructions
  - Tiered Response versus “All ALS” response
    - ALS = paramedic or higher; BLS = AEMT or lower
    - Tiered = based on call information, may send BLS unit to lower acuity calls
    - All ALS = send an ALS unit to every call
    - May also use call information to determine if/when lights and sirens are necessary
  - Most common EMS System types:
    - Fire-based EMS
    - Municipal EMS / Third Service Model
    - Private EMS
  - Fire-based EMS
    - Fire department runs EMS response as well as fire response
    - Pros: infrastructure already in place, more efficient for local government
    - Cons: EMS division often gets fewer resources/attention than Fire, crews may not like to cover medical calls
  - Municipal EMS / Third Service Model
    - Government runs EMS as a separate department
    - Pros: more focus on EMS, higher quality
    - Cons: more expensive, budget cuts, strain between departments
  - Private EMS
    - Government contracts with private company to provide EMS
    - Often run non-emergent transport calls in addition to 911 calls
    - Pros: least expensive for government, more financially responsible
    - Cons: government relinquishes control over operations and quality issues, some crews do not like having to do non-emergent transports
  - Fire First Response
    - Often compliments non fire-based EMS models
    - Fire department maintains non-transport, quick response vehicles and goes to calls along with EMS
    - Pros: often able to get to call quicker and initiate care, crews go back into service for fire response when EMS leaves the scene, less costly for government
    - Cons: often not reimbursed, continued issues with fire crews not wanting to run medical calls, strain between departments
- EMS Medicine Subspecialty
  - New subspecialty of Emergency Medicine
  - Approved in 2010, first test in 2013, test offered every 2 years
  - Core Content Areas:
    - Clinical Aspects
    - Medical Oversight
    - Special Operations
    - Quality Management and Research
  - Life of an EMS Doc
    - Admin
      - Medical oversight
      - QA/PI
    - Teaching
    - Field Response
    - Clinical time (ED)
- Summary

**Medical Oversight of EMS**
Lecture Outline

- Introduction
- Types of Oversight
  - Indirect Medical Oversight
    - Credentialing
    - Protocol / Guideline development
    - Quality Assurance / Process Improvement
    - Education
  - Direct Medical Oversight
    - Field Response
    - Online Medical Control (OLMC)
- Indirect Medical Oversight
  - Credentialing
    - EMS providers go through a very similar credentialing process to physicians
    - Medical director must confirm potential new hires have completed proper training, have adequate knowledge base, and can perform important skills
  - Protocol / Guideline development
    - EMS providers are expected to follow their service’s protocols / guidelines
      - Chief complaint / situation-based documents with instructions for care
    - Medical director must approve these guidelines and update them annually
  - Quality Assurance / Process Improvement
    - Quality Assurance = retrospective review of calls to ensure crews are following guidelines.
      - Remediation/reeducation/discipline if issues found
    - Process Improvement = proactive measuring of system-wide data and trends and implementing improvement plans based on that data
      - Policy change, new equipment, company-wide education initiatives
  - Education
    - EMS providers need continuing education for license renewal, which is often provided by the EMS service
    - Medical director must review and approve this education
    - Outreach education also common
    - Direct medical director involvement in teaching is well received
- Direct Medical Oversight
  - Field Response
    - Acts as a form of supervision and a chance to witness how EMS crews truly function in the field
    - Occasionally physician-level skills are needed (MCIs, prolonged care of entrapped patient, advanced procedures)
  - Online Medical Control (OLMC)
    - EMS crew calls into hospital and speaks directly with a physician regarding how to proceed in a certain situation
    - Base Station = centralized location for OLMC
- Base Station Physician Qualifications
  - Experience in Emergency Medicine
  - Clear understanding of his/her responsibility to the patient and the EMS provider
  - Familiar with local EMS system and protocols
  - Knowledgeable in the use of communication equipment and radio etiquette
  - Familiar with local EMS QA system to provide feedback
  - Base Station Physician Course (optional—based on state rules)
- Providing OLMC
  - Using the radio
    - Press and hold transmit button
    - Wait until the “chirp” finishes before talking
    - State your callsign and tell unit to go ahead with their request
    - Keep each transmission brief
    - Ask whatever questions you need to get the information to make the decision
  - OLMC Tips
    - Be familiar with local protocol
    - Don’t be afraid to ask all the questions you need
    - Always state a specific dose and route for medications
    - State “Stand by” if you need time to think
      - Avoid long pauses without acknowledging that you heard them
    - Don’t ask for patient identifying info over the radio
  - Patient refusals
    - Need to be fully oriented AND understand risks of refusing
    - If determined that they can’t refuse and the situation gets dangerous, get help from law enforcement
    - Pediatric patients (minors) can’t refuse unless parent/guardian agrees, emancipated minor, or related to pregnancy/STDs or mental health
  - OLMC and EMTALA
    - Radio contact with the hospital does NOT generate an EMTALA obligation
    - May divert an ambulance after radio contact if resources not available
- Summary
